# A decline in skeletal muscle NOX4 abrogates exercise-induced adaptive homeostasis and exacerbates biological aging

**DOI:** 10.1126/sciadv.adz1953

**Published:** 2026-06-10

**Authors:** Chrysovalantou E. Xirouchaki, Esther García-Domínguez, Eamon Coughlan, Meagan J. McGrath, Saveen Giri, Shuwei Liang, Florian Wiede, Anne Bigot, Junichi Sadoshima, Maria C. Gomez-Cabrera, Andrew Philp, Marcus Moberg, William Apró, William Roman, Christina A. Mitchell, Tony Tiganis

**Affiliations:** ^1^ Monash Biomedicine Discovery Institute, Monash University, Clayton, VIC 3800, Australia.; ^2^ Department of Biochemistry and Molecular Biology, Monash University, Clayton, VIC 3800, Australia.; ^3^ Australian Regenerative Medicine Institute, Monash University, Clayton, VIC 3800, Australia.; ^4^ Victoria Node, EMBL Australia, Clayton, Australia.; ^5^ Sorbonne Université, Inserm, Institut de Myologie, Centre de Recherche en Myologie, Paris, France.; ^6^ Department of Cell Biology and Molecular Medicine, Cardiovascular Research Institute, Rutgers New Jersey Medical School, Newark, NJ, USA.; ^7^ Freshage Research Group, Department of Physiology, University of Valencia, CIBERFES, Fundación Investigación Hospital Clínico Universitario/INCLIVA, Valencia, Spain.; ^8^ Centre for Healthy Ageing, Centenary Institute, Sydney, NSW, Australia.; ^9^ School of Sport, Exercise and Rehabilitation Sciences, Faculty of Health, University of Technology Sydney, Sydney, NSW, Australia.; ^10^ Department of Physiology, Nutrition and Biomechanics, The Swedish School of Sport and Health Sciences, Stockholm, Sweden.; ^11^ Department of Physiology and Pharmacology, Karolinska Institutet, Stockholm, Sweden.; ^12^ School of Sport, Exercise and Rehabilitation Sciences, University of Birmingham, Birmingham, UK.; ^13^ Department of Clinical Science, Intervention and Technology, Karolinska Institutet, Stockholm, Sweden.

## Abstract

A decline in nuclear factor erythroid 2–related factor 2 (NFE2L2)–orchestrated adaptive homeostasis and oxidative distress are thought to be key features of aging. In contracting skeletal muscle, the reactive oxygen species–producing enzyme NADPH oxidase 4 (NOX4) is a potent inducer of NFE2L2 adaptive homeostasis. Here, we report that skeletal muscle NOX4 levels decline in aged mice and humans, resulting in abrogated NFE2L2 adaptive homeostasis, increased protein oxidative damage, and decreased muscle function. We show that deleting NOX4 in skeletal muscle exacerbates the physiological decline associated with aging, resulting in overt sarcopenia and frailty, characterized by physical inactivity, increased adiposity, systemic inflammation, whole-body insulin resistance, and advanced liver disease in aged chow-fed mice. The systems-wide physiological decline in aged skeletal muscle NOX4-deficient mice could be corrected by restoring NOX4 using viral approaches or activating NFE2L2 downstream with sulforaphane and reinstating adaptive homeostatic responses otherwise induced by exercise. Our findings provide important insights into the basis for the decline in NFE2L2-orchestrated adaptive homeostasis that accompanies physical inactivity with age and identify key mechanisms by which exercise may promote healthy aging.

## Teaser

A decline in physical activity and NOX4/NFE2L2-orchestrated adaptive homeostasis in skeletal muscle accelerates biological aging.

## INTRODUCTION

All organisms are constantly exposed to varied internal and environmental stressors, including heat or cold shock, osmotic stress, nutrition, caloric restriction/starvation, hypoxia, and exercise training (*1*, *2*). Adaptations to stressors can increase resilience and allow organisms to manage damaging insults. The ability of organisms to transiently adapt to otherwise harmful stressors is known as adaptive homeostasis (*1*, *2*). A quintessential example of adaptive homeostasis is the response to oxidants, namely, reactive oxygen species (ROS). ROS are highly reactive chemicals generated in response to stressors and as biproducts of life in an aerobic environment (*3*, *4*). While evolution has harnessed specific ROS, for example, H_2_O_2_ for physiological roles such as cellular signaling, stressors resulting in redox imbalance and excess ROS can damage essential macromolecules including proteins, lipids, and DNA to promote cell death and inflammation and contribute to disease (*3*, *4*). The damaging effects of ROS or the inability to cope with ROS-mediated macromolecular damage have long been thought to be important contributors to aging (*1*, *2*).

One way by which organisms adapt to ROS is by activating an evolutionary conserved defense system orchestrated by the transcription factor nuclear factor erythroid 2–related factor 2 (NFE2L2) (*4*, *5*). NFE2L2 activation increases the abundance of hundreds of protective enzymes that limit ROS, ameliorate oxidative damage, eliminate damaged macromolecules and elicit long-lasting protection from subsequent exposures to ROS-producing stressors (*4*, *5*). NFE2L2 is normally targeted for degradation by the KEAP-1 (Kelch-like ECH-associated protein-1)/Cullin-3 E3 ligase complex. However, ROS can oxidize Cys residues (Cys^151^, Cys^273^, and Cys^288^) on KEAP-1 to facilitate NFE2L2’s release and translocation to the nucleus where, together with small MAF (musculoaponeurotic fibrosarcoma) subfamily transcription factor, it binds to antioxidant response elements (AREs) in the promoter regions of genes (*4*, *5*). NFE2L2 can drive its own expression as well as the expression of >200 ARE-containing antioxidant/detoxifying and cytoprotective enzymes (*4*–*6*). These include enzymes involved in NADPH production necessary for the reduction of oxidized glutathione, including NAD(P)H dehydrogenase (quinone 1) (NQO1) (*7*) as well as the expression of ROS detoxification enzymes, including superoxide dismutase 1 (SOD1) and mitochondrial SOD2 that dismutate superoxide (O2•^−^) into H_2_O_2_, and peroxiredoxins (PRDXs), glutathione peroxidase 1 (GPX1), and catalase that eliminate H_2_O_2_ (*4*, *5*). NFE2L2 also drives the expression of components of the 20*S* proteosome to facilitate the degradation of damaged proteins (*8*, *9*). It regulates the cargo recognition protein p62/sequestosome 1 that facilitates the autophagic degradation of protein aggregates and organelles (*10*). In addition, NFE2L2 directly and indirectly inhibits inflammation (*11*) and supports mitochondrial function, in part by driving the expression of mitochondrial biogenesis genes, including *Pcg1a* (which encodes peroxisome proliferator-activated receptor gamma coactivator 1) (*12*–*14*). Growing evidence indicates that aging is associated with a decline in NFE2L2 activity, oxidative distress, and the damage of macromolecules (*15*–*19*). There is a growing appreciation that NFE2L2-orchestrated adaptive homeostasis is compromised during aging, contributing to the accumulation of damaged proteins, inflammation, and the deterioration of cellular function (*1*, *2*, *19*).

In mammals, skeletal muscle constitutes 30 to 50% of body weight and contracting skeletal muscle is a major producer of ROS and a potent inducer of NFE2L2-orchestrated adaptive homeostasis (*20*–*22*). Such adaptive responses include the promotion of antioxidant defense, mitochondrial biogenesis, glucose uptake/glycogen synthesis, and insulin sensitivity (*20*–*24*). Therefore, adaptive responses to muscle contraction during exercise serve to bolster respiratory capacity and endurance, afford metabolic flexibility, limit oxidative damage, and promote insulin sensitivity to deal with subsequent bouts of intense physical activity. While mitochondria generate the majority of steady state O2·^−^ in muscle cells, NADPH oxidase 2 (NOX2) and NOX4 are primarily responsible for ROS generation during muscle contraction and exercise (*4*, *21*). NOX2 and NOX4 are differentially localized in skeletal muscle; while NOX2 localizes to the plasma membrane of sarcolemma and transverse tubules, NOX4 localizes to the sarcoplasmic reticulum, transverse tubules, and the inner mitochondrial membrane (*21*, *25*–*27*). Although both NOXs can generate O2·^−^, NOX4 is unique in its ability to also directly generate H_2_O_2_ (*28*). The expression of NOX4 and, to a lesser extent, the catalytic subunit of NOX2 (encoded by *Cybb*) are induced when mice are exercised (*29*). In addition, elegant biosensor studies have shown that NOX2 is activated and generates ROS in contracting skeletal muscle (*30*). The activation of NOX2 has been reported to promote glucose uptake during moderate intensity exercise without overtly altering mitochondrial biogenesis or enhancing insulin sensitivity (*21*, *30*–*32*). Using a whole-body loss-of-function model of NOX2, Henriquez-Olguin *et al.* (*21*, *33*) additionally reported that NOX2 deficiency compromises the mitochondrial network remodeling and blunts the improvements in exercise capacity otherwise associated with 6 weeks of high-intensity interval training. By contrast, we and others have shown that NFE2L2 adaptive responses in both skeletal and cardiac muscle following acute moderate- or high-intensity exercise or 5-week exercise training are reliant on NOX4 and the generation of H_2_O_2_ (*21*, *29*, *34*). In particular, in skeletal muscle, the induction of NOX4 after exercise and the resultant increased H_2_O_2_ is essential for the degradation of KEAP-1 and the stabilization of NFE2L2 to drive mitochondrial biogenesis and enhance muscle function and exercise capacity (*29*). NOX4 also plays a critical role in promoting NFE2L2-driven antioxidant defense mechanisms that limit mitochondrial oxidative stress and prevent the oxidative damage of proteins and lipids, which otherwise impairs insulin signaling and promotes insulin resistance (*29*).

We have shown previously that skeletal muscle NOX4 but not NOX2 levels decline in aged mice, or high-fat diet–fed obese mice, to promote insulin resistance (*29*). In this study, we have examined the importance of the decline in NOX4 on NFE2L2 adaptive responses in aged mice and determined the extent to which this might contribute to oxidative distress and the systemic physiological decline associated with aging.

## RESULTS

### Human skeletal muscle NOX4 and NFE2L2 adaptive homeostasis decline during aging

**Fig. 1.**
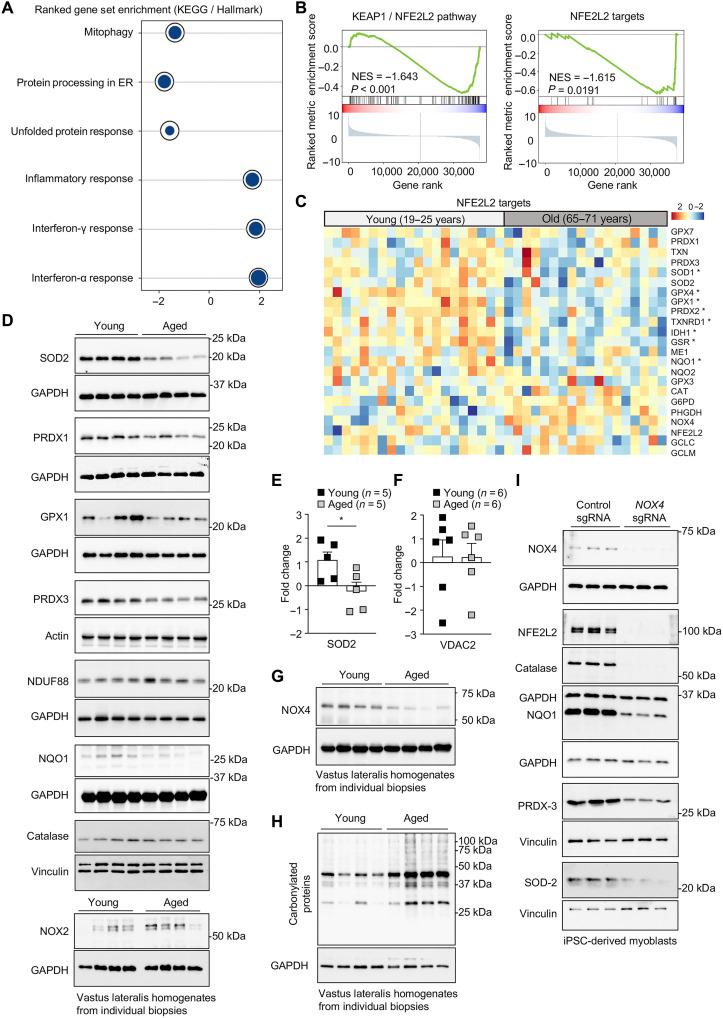
Skeletal muscle NOX4 abundance and NFE2L2 adaptive homeostasis decline during aging in humans. (**A** to **C**) RNA-seq data from GSE159217 was analyzed for differential gene expression in human male vastus lateralis muscle from aged (65 to 71 years old, *n* = 18) and exercise-matched young (19 to 25 years old, *n* = 20) participants. (A) Dot plot highlighting significant (*P* < 0.05) negative enrichment of unfolded protein response (Hallmark gene sets) and ER processing and mitophagy (KEGG pathways) genes in aged males, and significant (*P* < 0.05) positive enrichment of interferon-α and -γ response and inflammatory response (Hallmark gene sets) genes in aged males. Circle diameters within rings indicate proportions of the reference gene sets present in the dataset. (B) Barcode plots demonstrating negative enrichment of Reactome KEAP1/NFE2L2 Pathway (left) and NFE2L2 curated target set (right) in aged males. (C) Heatmap of expression levels of the curated set of NFE2L2 targets across the cohort. Genes with significantly down-regulated expression (log fold change < 0, *P* < 0.05) are marked with an asterisk. (**D**) Skeletal muscle vastus lateralis homogenates from young (27.6 ± 6.6 years old) and aged (69.9 ± 3.1 years old) men that were matched for BMI were analyzed by immunoblotting. (**E** and **F**) Mass spectrometry–based proteomic analyses of mitochondria-enriched fractions from hip skeletal muscle biopsies of BMI-matched aged, physically inactive (86.7 ± 9.7 years old) versus young, physically fit (37.3 ± 10.6 years old) individuals to assess (E) SOD2 and (F) VDAC protein levels. (**G** and **H**) Skeletal muscle vastus lateralis homogenates from BMI-matched young versus aged men were assessed by immunoblotting for (G) NOX4 protein, and (H) protein carbonylation (Oxyblot). (**I**) Human iPSCs were differentiated into myoblasts and *NOX4* deleted by CRISPR RNP gene editing. Cells were processed for immunoblotting. Representative and quantified results are shown (means ± SEM); significance was determined using a Student’s *t* test (D to I) (see section “Quantification and statistical analysis”).

To explore the extent to which perturbations in skeletal muscle adaptive homeostasis may contribute to aging and the decline in physiological integrity in humans, we took advantage of a publicly available RNA sequencing (RNA-seq) dataset (GSE159217) assessing the effects of aging on vastus lateralis skeletal muscle gene expression in young (19 to 25 years old, *n* = 20) versus old (65 to 71 years old, *n* = 18) male participants (*35*). Previous studies using this dataset identified oxidative metabolism as the principal pathway down-regulated in the muscle of aged individuals (*35*); this was accompanied by the down-regulation of mitochondrial biogenesis genes and mitochondrial respiratory proteins for complexes I and IV (*35*). As NFE2L2 is considered essential for skeletal muscle mitochondrial biogenesis in response to exercise (*12*, *14*, *36*), we focused on pathways regulated by NFE2L2, including the unfolded protein response, ER processing, and mitophagy pathways that are induced by NFE2L2 and the inflammatory pathway that is repressed by NFE2L2 (*9*–*11*, *37*). We found that aging was associated with an overt repression in NFE2L2 responses, as reflected by the decline in unfolded protein response, ER processing, and mitophagy pathways and the induction of interferon-α (IFN-α)/IFN-γ and inflammatory response pathways (Fig. 1A and fig. S1A). Consistent with this, we found that the Reactome KEAP1/NFE2L2 Pathway gene set was significantly down-regulated in the skeletal muscle of aged individuals [normalized enrichment score (NES) = −1.643, *P* < 0.001] (Fig. 1B). Furthermore, an analysis of a curated NFE2L2 target gene set focused on antioxidant defense (table S1) revealed that aging was associated with a decline in skeletal muscle antioxidant defense (NES = −1.615, *P* = 0.019) (Fig. 1C). In particular, genes encoding key antioxidant defense enzymes, including mitochondrial-targeted SOD2 that eliminates mitochondrial O2·^−^ by converting it to H_2_O_2_ (*38*, *39*), SOD1 that similarly eliminates O2·^−^ in the cytosol, GPX1 and PRDX2 that eliminate H_2_O_2_ in the cytosol, PRDX-3 that eliminates H_2_O_2_ in mitochondria, and NQO1 that is exclusively regulated by NFE2L2 in muscle (*7*), were decreased in the skeletal muscle of aged participants (Fig. 1C and fig. S1B); other *NFE2L2* target genes including the gene for NOX4 (*NOX4*) that generates H_2_O_2_ as part of a feed forward loop to drive NFE2L2 adaptive responses (*29*, *40*, *41*) and catalase (*CAT*) that eliminates H_2_O_2_ at peroxisomes and within the cytosol were not decreased (Fig. 1C). By contrast, *CYBB* that encodes the catalytic subunit of NOX2 that generates O2•^−^ was elevated in the skeletal muscle of aged participants (fig. S1B). Unlike skeletal muscle, an analysis of another publicly available RNA-seq dataset (GSE141910) revealed no significant differences in NFE2L2 pathway genes in otherwise healthy hearts from old (71 to 83 years old; 15 females and 8 males) versus young (15 to 26 years; 4 females and 7 males) participants, suggesting that the decline in NFE2L2 antioxidant defense gene expression is unlikely to be a primary immutable outcome of aging (fig. S1C).

To ascertain whether reductions in antioxidant gene expression in the skeletal muscle of aged participants may be associated with decreased antioxidant defense and increased oxidative damage, we took advantage of skeletal muscle vastus lateralis biopsies from physically active young (27.6 ± 6.6 years old) and aged (69.9 ± 3.1 years old) men that were matched for body mass index (BMI_young_ = 24.3 ± 0.2; BMI_aged_ = 22.9 ± 0.1) (fig. S2A) and monitored for changes in the abundance of antioxidant enzymes as well as the oxidative damage of proteins (protein carbonylation) by immunoblotting (Fig. 1, D and H). Consistent with our gene expression analyses, we found that SOD2, PRDX1, PRDX3, GPX1, and NQO1 were down-regulated in skeletal muscle lysates of aged men, whereas catalase levels were not altered (Fig. 1D and fig. S2B). Moreover, the decreased abundance of mitochondrial SOD2 was independently substantiated using mass spectrometry-based proteomic analyses of mitochondria-enriched fractions from hip skeletal muscle biopsies of BMI-matched aged, physically inactive (86.7 ± 9.7 years old, *n* = 11, 4 males and 7 females; BMI = 26.9 ± 3 kg/m^2^) versus young, physically fit (37.3 ± 10.6 years old, *n* = 8, 5 males and 3 females; BMI = 27 ± 4.6 kg/m^2^) individuals (Fig. 1E and fig. S3A). As a control for mitochondrial content, we also monitored for the abundance of the voltage-dependent anion channel 2 (VDAC2) protein that is localized in the outer mitochondrial membrane; VDAC2 levels were similar between young and aged samples (Fig. 1F). By contrast, mitochondrial complex proteins that are known to decline with aging were reduced (fig. S3B). In addition, despite *NOX4* mRNA (GSE159217) not being reduced in vastus lateralis skeletal muscle from aged versus young men (Fig. 1C), an analysis of NOX4 protein levels [assessed using a validated antibody (*29*)] in whole skeletal muscle lysates from BMI-matched aged versus young men revealed that this enzyme that is required for H_2_O_2_ production and the induction of NFE2L2 adaptive responses in muscle (including mitochondrial biogenesis and antioxidant defense) after exercise (*29*) was reduced (Fig. 1G and fig. S2C); by contrast, protein levels for the NOX2 catalytic subunit were not reduced (Fig. 1G). The decreased abundance in skeletal muscle NOX4 and enzymes involved in antioxidant defense was, in turn, accompanied by increased oxidative damage, as assessed by monitoring for protein carbonylation in the skeletal muscle lysates of aged versus young men (Fig. 1H). Together, these results point toward NOX4 and NFE2L2 adaptive responses, including antioxidant defense, declining in human skeletal muscle during aging.

Skeletal muscle is a complex tissue consisting of not only multinucleated muscle fibers, but also immune cells, endothelial cells, muscle stem cells, and other mononuclear cells important for muscle homeostasis and exercise responses (*42*–*46*) that change with aging. Therefore, we sought to determine whether the decline in antioxidant defense may be cell autonomous and attributed to the decline in NOX4 in human muscle cells. To this end, we first took advantage of human induced pluripotent stem cells (hiPSCs) and differentiated these into myoblasts as defined by the nuclear expression of the myogenic transcription factor MyoD (fig. S4A) and deleted *NOX4* by CRISPR-Cas9 ribonucleoprotein (RNP) gene editing; myoblasts were cultured in the presence of 5% O_2_ to limit oxidative stress (Fig. 1I and fig. S4B). The deletion of *NOX4* in myoblasts reduced NOX4 protein by >90% and was accompanied by an overt decline not only in NFE2L2 protein, but also SOD2, PRDX3, GPX1, and catalase protein levels (Fig. 1I and fig. S4C); mitochondrial content as reflected by the abundance electron transport chain (ETC) complex I protein NDUF88 was not altered (fig. S4D). The deletion of *NOX4* and decline in NFE2L2 protein were accompanied by a decline in NFE2L2’s abundance in the nucleus where it mediates its transcriptional responses; this was evident in myoblasts (fig. S5A) and differentiated myotubes (fig. S5B). Moreover, the deletion of *NOX4* and the decline in NFE2L2 antioxidant defense were associated with increased mitochondrial O2•^−^ levels, as detected by the mitochondrial O2•^−^ probe MitoSOX Red in myoblasts (fig. S5C) and increased protein carbonylation in myotubes (fig. S4E). The reduced NFE2L2 protein (Fig. 1I and fig. S4F) and NFE2L2 nuclear localization (fig. S5, A and B), the reduced abundance of antioxidant defense enzymes (Fig. 1I and fig. S4F), and ensuing increased mitochondrial O2•^−^ (fig. S5C) and protein carbonylation in NOX4-deficient cells (fig. S4, E and G) were corrected by the concomitant deletion of *KEAP1* using CRISPR-Cas9 RNP gene editing (figs. S4, E to G, and S5, A to C). This is consistent with our previous studies showing that NOX4-derived H_2_O_2_ stabilizes NFE2L2 to drive antioxidant defense and temper mitochondrial distress and the oxidative damage of macromolecules (*29*, *41*). Furthermore, our findings in induced pluripotent stem cell (iPSC)–derived muscle cells were recapitulated in an immortalized myoblast cell line (AB1190) from a healthy human donor (fig. S4, H to J), wherein the deletion of *NOX4* reduced NFE2L2, SOD2, and NQO1 (fig. S4I) and increased protein carbonylation (fig. S4J); both were rescued by the concomitant deletion of *KEAP1* (fig. S4, H to J). Together, our results are consistent with the apparent decline in NFE2L2 adaptive homeostasis and oxidative distress in the skeletal muscle of aged humans being attributed to the decline in NOX4 in muscle cells.

### Skeletal muscle NOX4 and NFE2L2 adaptive homeostasis decline during aging in mice

**Fig. 2.**
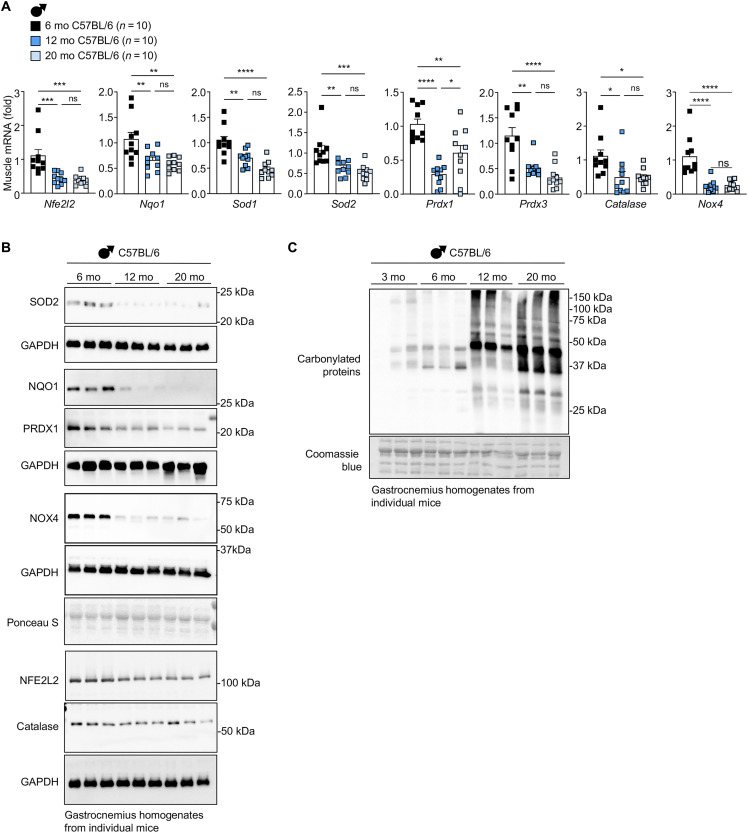
Skeletal muscle NOX4 and antioxidant defense decline during aging in mice. Male C57BL/6 mice were fed a standard chow diet (4.8% fat) for 3, 6, 12, or 20 months as indicated. Gastrocnemius muscle was extracted and analyzed by (**A**) quantitative real-time PCR (qPCR) or (**B**) immunoblotting. (**C**) Alternatively, muscles were processed for analysis of protein carbonylation (Oxyblot) or Coomassie blue staining. Representative and quantified results are shown (means ± SEM) for the indicated number of mice; significance was determined using a one-way ANOVA (see section “Quantification and statistical analysis”).

To determine whether NFE2L2-orchestrated antioxidant defense may also decline in the skeletal muscle of aging mice, we monitored for the expression of NFE2L2 target genes by quantitative real-time PCR (qPCR) in the gastrocnemius muscle of 6-, 12-, and 20-month-old male versus female C57BL/6 mice fed a standard chow diet (4.8% fat). Key antioxidant defense genes including those encoding *Nfe2l2*, *Nqo1*, *Sod1*, *Sod2*, *Prdx1*, *Prdx3*, and *Cat* started to decline in both male and female mice by 12 months of age (Fig. 2A and fig. S6A); this persisted at 20 months of age for all antioxidant defense genes, with the exception of *Cat* in female mice and *Prdx1* in male mice, which increased but was nonetheless lower than that in 6-month-old mice (Fig. 2A and fig. S6A). The decline in antioxidant defense gene expression was accompanied by a decline in the abundance of NFE2L2, SOD2, PRDX1, and NQO1 proteins as well as a modest albeit nonsignificant decline in catalase levels as assessed by immunoblot analysis in the gastrocnemius muscle of male mice (Fig. 2B and fig. S6B). Furthermore, we found that skeletal muscle NOX4 protein and/or *Nox4* mRNA also declined by 12 months of age in male and female chow-fed mice and persisted at 20 months of age (Fig. 2, A and B, and fig. S6, A and B). The decline in NOX4 and NFE2L2 antioxidant defense was, in turn, accompanied by increasing oxidative damage as reflected by the increasing carbonylation of proteins with age (Fig. 2C). Together, these results point toward the decline in NOX4 with age abrogating NFE2L2-orchestrated antioxidant defense to promote progressively worsening macromolecular oxidative damage.

### NOX4 deficiency abrogates NFE2L2 adaptive responses and promotes sarcopenia with age

**Fig. 3.**
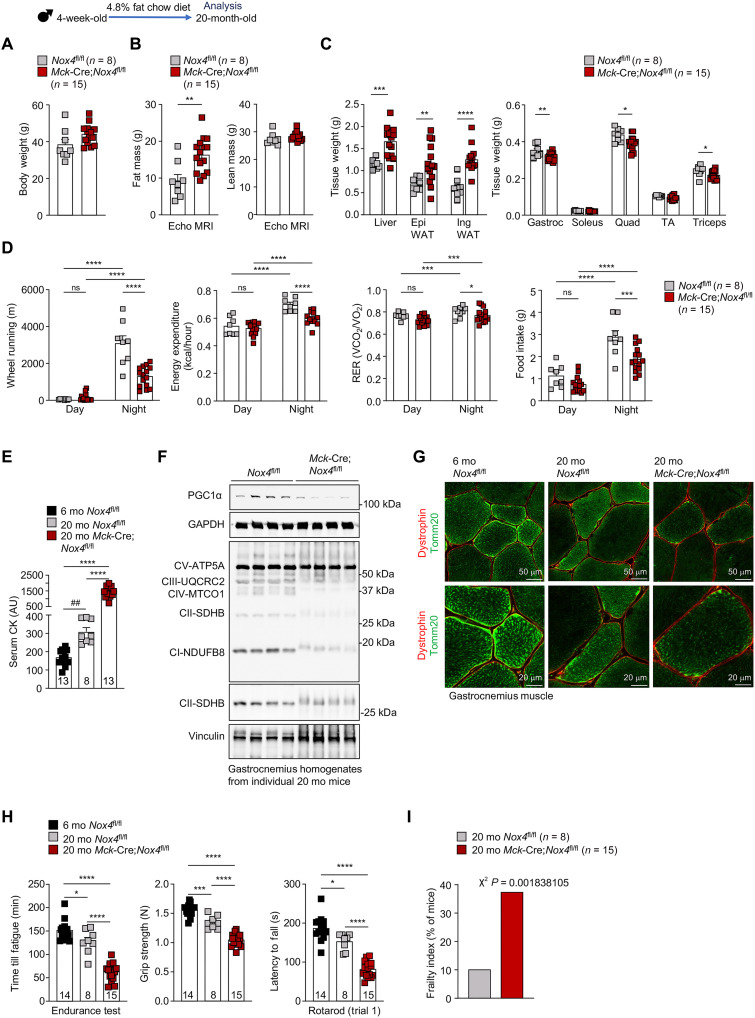
NOX4 deficiency promotes sarcopenia and exacerbates the decline in muscle function with aging. *Nox4*^fl/fl^ control and *Mck*-Cre;*Nox4*^fl/fl^ muscle-specific NOX4-deficient male mice were fed a standard chow diet (4.8% fat) for 20 months (mo). (**A**) Body weights, (**B**) body composition (EchoMRI) and (**C**) tissue weights [liver, epididymal (Epi), and inguinal (Ing) white adipose tissue (WAT) and gastrocnemius (Gastroc), soleus, quadriceps (Quad), and tibialis anterior (TA) skeletal muscles] were analyzed. (**D**) Ambulatory activity (wheel running), energy expenditure, RERs, and food intake were analyzed in metabolic cages (Promethion). (**E** to **G**) Six- or 20-month-old *Nox4*^fl/fl^ and Mck-Cre;*Nox4*^fl/fl^ male mice as indicated were fed a standard chow diet (4.8% fat) and (E) serum isolated to assess creatine kinase (CK) levels, or gastrocnemius muscles dissected and processed for (F) immunoblotting to monitor PGC1α protein levels and OXPHOS protein complexes, or (G) immunostaining monitoring for Tomm20 and dystrophin to define mitochondria within individual muscle fibers. (**H**) Six- or 20-month-old *Nox4*^fl/fl^ and Mck-Cre;*Nox4*^fl/fl^ male mice as indicated were fed a standard chow diet (4.8% fat) and subjected to endurance tests, grip strength measurements, and motor coordination tests (rotarod). (**I**) Twenty-month-old male *Nox4*^fl/fl^ and *Mck*-Cre;*Nox4*^fl/fl^ mice fed a standard chow diet were subjected to frailty score assessments according to the Valencia system. Representative and quantified results are shown (means ± SEM) for the indicated number of mice; significance determined by Student’s *t* test (A to C), two-way ANOVA (D), one-way ANOVA (E and H), or a chi-square test (I); where indicated (#), significance was determined by Student’s *t* test (see section “Quantification and statistical analysis”).

To explore the extent to which the decline in NOX4 may contribute to the physiological decline associated with aging, we sought to ablate NOX4 in skeletal muscle. To this end, we crossed *Nox4^fl/fl^* mice (*47*) with those expressing Cre under the control of muscle creatinine kinase (*Mck*) promoter (*Mck*-Cre) where Cre is expressed predominantly in skeletal muscle and, to a lesser extent, in cardiac muscle (*48*). We have previously shown that *Nox4* is effectively deleted in skeletal muscle in *Mck*-Cre;*Nox4*^fl/fl^ mice, and this is accompanied by decreased resting and exercised-induced skeletal muscle H_2_O_2_ production, decreased exercise-induced oxidation of glutathione, and decreased exercise capacity (*29*); *Nox4* mRNA is only moderately reduced in heart, but resting or exercise-induced cardiac H_2_O_2_ levels, or cardiac function, are not altered in *Mck*-Cre;*Nox4*^fl/fl^ mice (*29*). *Nox4*^fl/fl^ control and *Mck*-Cre;*Nox4*^fl/fl^ muscle-specific NOX4-deficient male and female mice were fed a 4.8% fat standard chow diet for 6 or 20 months, and effects on aging were assessed; body weights in *Nox4*^fl/fl^ control mice fed a 4.8% fat chow diet for 20 months averaged <40 g. We have reported previously that *Nox4*^fl/fl^ control and *Mck*-Cre;*Nox4*^fl/fl^ fed a standard breeding diet (8.5% fat) for 20 months, or even a high-fat diet (23.5% fat; 46% energy from fat) for 20 weeks, so that body weights in each case averaged ~50 g, do not exhibit any difference in body weights between genotypes (*29*). Consistent with this, body weights and body composition were not altered in 6-month-old *Mck*-Cre;*Nox4*^fl/fl^ versus *Nox4*^fl/fl^ male or female mice fed a 4.8% fat chow diet (fig. S7, A and B). By contrast, by 20 months of age, *Nox4*^fl/fl^ male or female mice fed a 4.8% fat chow diet gained less weight than those fed the 8.5% fat diet and male NOX4-deficient mice fed the 4.8% fat chow diet tended to gain more weight, whereas the corresponding female mice gained significantly more weight than *Nox4*^fl/fl^ controls. In both male and female mice, this was attributed to increased adiposity (Fig. 3, A and B, and fig. S8, A and B), as reflected by body composition analyses (EchoMRI) and white adipose tissue (WAT) weights (inguinal and gonadal) (Fig. 3, B and C); no differences were evident in lean mass (Fig. 3B and fig. S8A) as assessed by EchoMRI (measures smooth, cardiac, and skeletal muscles plus all organs). The increased adiposity at 20 months of age was accompanied by decreased voluntary wheel running and energy expenditure (Fig. 3D and fig. S8C); respiratory exchange ratios (RERs; indicative of carbohydrate versus fat oxidation) were modestly reduced in male but not female mice during the night phase (Fig. 3D and fig. S8C). Paradoxically, in male mice, NOX4 deficiency was also accompanied by decreased food intake at 6 and 20 months of age (Fig. 3D and fig. S9A). The decreased food intake coincided with the activation of the integrated stress response (ISR) as early as 6 months of age (fig. S9B). The ISR is a conserved cellular stress response that down-regulates protein synthesis and up-regulates the expression of specific metabolism-correcting genes in response to stressors, including impairments in mitochondrial function, ER stress, and macromolecular oxidative damage (*49*). Central to the ISR is the phosphorylation of eukaryotic translation initiation factor 2 α (eIF2α) on Ser^51^ that allows for eIF2α to decrease overall protein synthesis while driving the translation of select genes, such as activating transcription factor 4 (ATF4) (*49*). ATF4 can drive the expression of the transcription factor C/EBP homologous protein (CHOP), DNA damage-inducible protein (GADD34), and secreted factors such as fibroblast growth factor 21 (FGF21) and growth differentiation factor 15 (GDF15) that alter systemic metabolism (*49*–*52*) to increase energy expenditure and repress feeding respectively (*49*, *53*–*56*). We found that eIF2α Ser^51^ phosphorylation and CHOP-1, GADD34, and FGF21 protein levels were increased in *Mck*-Cre;*Nox4^fl/fl^* gastrocnemius skeletal muscle (fig. S9B) and accompanied by increased FGF21 (fig. S9C) and GDF15 in serum (fig. S9D). The increased GDF15 in blood in *Mck*-Cre;*Nox4^fl/fl^* mice is consistent with the observed decreased feeding.

Irrespective, we found that 20-month-old male and especially female NOX4-deficient mice were characterized by decreased skeletal muscle weights; this was evident for different skeletal muscles, including those composed of oxidative and glycolytic (gastrocnemius and quadriceps) or predominantly glycolytic [tibialis anterior (TA) and triceps] fiber types (Fig. 3C and fig. S8B); NOX4 deficiency did not decrease gastrocnemius, quadriceps, TA, or triceps muscle weights in 3- to 12-month-old mice. The decreased skeletal muscle weights in aged mice were accompanied by increased circulating levels of creatinine kinase (CK) (Fig. 3E); CK is abundant in skeletal muscle, but normally low in circulation, and its presence can be indicative of muscle damage (*57*). Nonetheless, there were no gross signs of muscular pathological abnormalities as reflected by histology [hematoxylin and eosin (H&E)] and dystrophin staining (marks muscle fiber perimeters) (fig. S10A). Also, no alterations were evident in gastrocnemius myofiber size or the abundance of oxidative (expressing type I fiber) and glycolytic (expressing type IIa, type IIb, and type IIx fibers) muscle fibers (fig. S10, A and B), consistent with previous studies showing that NOX4 deficiency does not alter fiber-type abundance in skeletal muscle (*29*, *58*). However, NOX4 deficiency was accompanied by an exacerbated decline in mitochondrial biogenesis and content, as reflected by the decline in PGC1α (peroxisome proliferator-activated receptor gamma coactivator 1-alpha) protein that otherwise promotes mitochondrial biogenesis (*24*, *59*) and the decreased abundance of mitochondrial complex proteins as assessed by immunoblotting (Fig. 3F and fig. S11, A and B). Consistent with this, histological/immunohistochemical analyses revealed that NOX4 deficiency decreased both subsarcolemmal and intermyofibrillar succinate dehydrogenase (SDH; serves as complex II of the ETC) and Tomm20 (a mitochondrial import receptor protein) staining in gastrocnemius muscle (Fig. 3G and fig. S10C). Although NOX4 deficiency decreased skeletal muscle SDH and Tomm20 staining as early as 6 months of age, this was exacerbated by 20 months of age (fig. S10, C and D); differences in skeletal muscle PCG1α, mitochondrial complex proteins, and SDH and Tomm20 staining were not evident at 3 months of age (fig. S12), consistent with the decline in mitochondrial content being an age-related phenomenon. Together, the progressive decline in muscle mass accompanied by the increased serum CK, along with the exacerbated decline in mitochondrial content, point toward NOX4 deficiency contributing to muscle wasting or the development of sarcopenia, a hallmark of aging.

Next, we sought to assess the impact of NOX4 deficiency on muscle function (Fig. 3H and fig. S13), which, along with the decreased muscle mass and mitochondrial function/content, is known to worsen with age. We first assessed the impact of NOX4 deficiency on exercise capacity, by subjecting mice to endurance tests till exhaustion. Although NOX4 deficiency was accompanied by a decline in exercise capacity at 6 months of age, this was exacerbated by 20 months of age in both male and female mice (Fig. 3H and fig. S13, A and B). Similarly, NOX4 deficiency exacerbated the decline in muscle strength (as assessed by measuring forelimb grip strength) by 20 months of age, at least in male mice (Fig. 3H and fig. S13, C and D). In addition, NOX4 deficiency exacerbated the decline in motor coordination and balance (as assessed in rotarod tests) in male mice (Fig. 3H and fig. S13, E and F), whereas in female mice, it was already markedly reduced by 6 months of age (fig. S13, E and F). In mice, the following criteria can be used to score frailty: unintentional weight loss, poor endurance (running time), slowness (running speed), muscle weakness (grip strength), and low activity levels (motor coordination) (*60*, *61*). Together these criteria constitute the Valencia score for frailty and can be used to predict premature aging (*60*, *61*). Although we did not observe a decline in total body weight in 20-month-old *Mck*-Cre;*Nox4^fl/fl^* mice, this was due to the increased adiposity accompanying the decline in locomotor activity and energy expenditure (Fig. 3, A to D, and fig. S8). Using the change in lean mass between 6- and 20-month-old *Nox4^fl/fl^* versus *Mck*-Cre;*Nox4^fl/fl^* male mice rather than total body weight, along with our endurance tests, grip strength tests, and rotarod neuromuscular coordination tests in 20-month-old mice, we were able to calculate a surrogate frailty score that showed that *Mck*-Cre;*Nox4^fl/fl^* male mice exhibited a marked increase in frailty (Fig. 3I). Therefore, together our results point toward NOX4 deficiency exacerbating the decline in skeletal muscle function and the development of frailty.

**Fig. 4.**
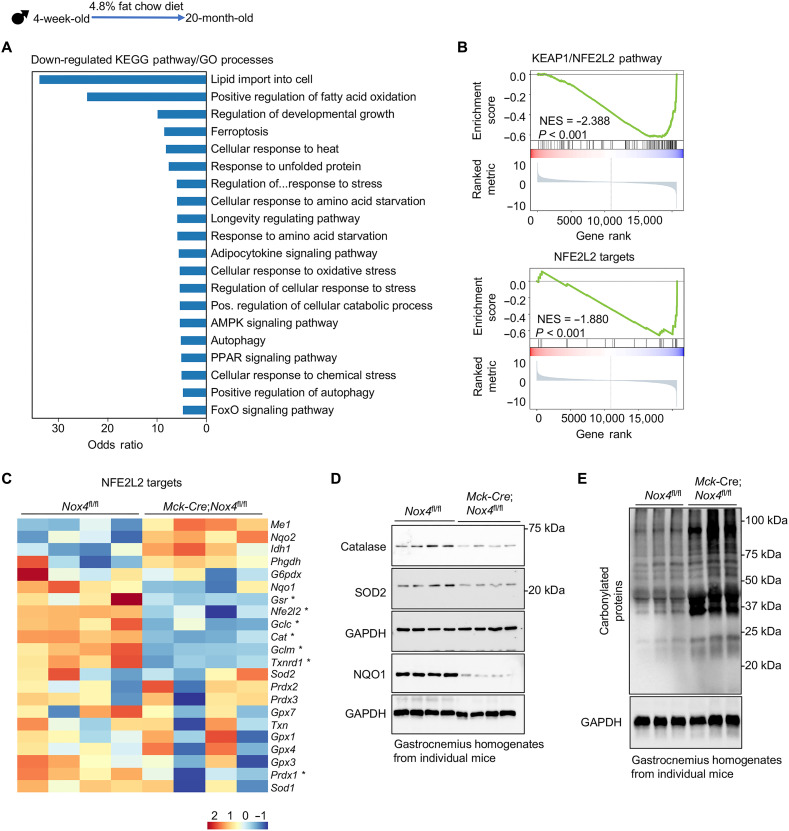
Muscle NOX4 deficiency abrogates NFE2L2-mediated antioxidant defense in aged mice. Gastrocnemius from 21-month-old *Nox4*^fl/fl^ and *Mck*-Cre;*Nox4*^fl/fl^ male chow (4.8% fat)–fed mice (*n* = 4 per genotype) was processed for bulk RNA-seq. (**A**) Bar plot of the top 20 KEGG pathways/GO biological process terms overrepresented among genes down-regulated in *Mck*-Cre;*Nox4*^fl/fl^ mice. Pathways significant with FDR <0.01 were ranked by odds ratio as determined by analysis with the enrichR function in gseapy. “Regulation of…response to stress” = Regulation of transcription from RNA polymerase II promoter in response to stress. (**B**) Negative enrichment of the Reactome KEAP1/NFE2L2 Pathway (top) and NFE2L2 gene set (bottom) in NOX4-deficient animals, demonstrating impairment of ROS response mimicking that observed in older humans. (**C**) Heatmap of NFE2L2 gene set expression. Genes with significantly down-regulated expression (log fold change <0, *P* < 0.05; analyzed with DESeq2) are marked with an asterisk (see section “Quantification and statistical analysis”). (**D**) Gastrocnemius muscle from 20-month-old *Nox4*^fl/fl^ and *Mck*-Cre;*Nox4*^fl/fl^ male chow (4.8% fat)–fed mice processed for immunoblotting to assess SOD2, NQO1, and catalase protein levels and (**E**) protein carbonylation (Oxyblot). Representative and quantified results are shown.

For an unbiased perspective of the impact of NOX4 deficiency on skeletal muscle adaptive responses in 20-month-old aged mice, we subjected gastrocnemius muscle from 20-month-old *Mck*-Cre;*Nox4*^fl/fl^ versus *Nox4*^fl/fl^ male mice fed the 4.8% fat chow diet to transcriptomics analysis by bulk RNA-seq (Fig. 4 and fig. S14A). Differential gene expression analysis revealed that 1023 genes were differentially expressed, including 580 genes down-regulated and 443 genes up-regulated (fig. S14A). Gene Ontology (GO) analysis revealed that genes associated with the response to stressors, including the cellular response to heat, amino acid starvation, starvation, and heat and oxidative stress, were significantly down-regulated [false discovery rate (FDR) < 0.01] and accompanied by the down-regulation of the response to unfolded protein, consistent with abrogated NFE2L2 adaptive homeostasis and exacerbated aging (Fig. 4A). In addition, fatty acid oxidation pathways and those involved with the response to insulin otherwise necessary for the provision of fuel during exercise and for replenishing glycogen reserves after exercise were also down-regulated (Fig. 4A). Similarly, Kyoto Encyclopedia of Genes and Genomes (KEGG) analysis revealed that numerous pathways normally induced as part of NFE2L2 adaptive homeostasis and down-regulated during aging were significantly down-regulated (FDR < 0.01) by NOX4 deficiency in 20-month-old mice; these included genes linked to autophagy, mitophagy, and protein processing as well as peroxisome proliferator-activated receptor (PPAR) and AMP-activated protein kinase (AMPK) signaling that are linked to fatty oxidation, exercise-induced mitochondrial biogenesis, and glucose uptake (Fig. 4A). Consistent with these analyses, we found that the KEAP1/NFE2L2 pathway (NES = −2.388, *P* < 0.001) and a curated NFE2L2 gene set (table S1) focused on antioxidant defense (NES = −1.88, *P* < 0.001) were significantly down-regulated (Fig. 4, B and C). By contrast, *Cybb* expression encoding the catalytic subunit of NOX2 was not significant altered by NOX4 deficiency (fig. S14B). Although we noted variability in select NFE2L2 target genes in aged control mice, this may have been linked to varying physical activity and NOX4/NFE2L2-induced responses, as *Nox4* mRNA levels varied and correlated with the extent of voluntary wheel running in 20-month-old *Nox4*^fl/fl^ mice (*R* = 0.994, *P* = 1.006) (fig. S14C). Irrespective, the overt decline in pathways linked to NFE2L2-orchestrated antioxidant defense in the gastrocnemius muscle of 20-month-old *Mck*-Cre;*Nox4*^fl/fl^ mice was reaffirmed at the protein level by immunoblotting for SOD2, NQO1, and catalase (Fig. 4D). Although skeletal muscle NFE2L2 targets including SOD2, NQO1, and catalase were already reduced in 6-month-old *Mck*-Cre;*Nox4*^fl/fl^ mice (fig. S14D) (*29*), they were reduced further by 20 months of age (Fig. 4D and fig. S14D). Furthermore, the decline in antioxidant defense accompanying NOX4 deficiency in skeletal muscle was, in turn, associated with the increased oxidative damage of proteins as reflected by protein carbonylation (Fig. 4E). Together, our findings are consistent with skeletal muscle NOX4 deficiency abrogating NFE2L2 adaptive homeostasis to exacerbate the age-associated oxidative damage of macromolecules, and the decline in muscle mass, mitochondrial content, and muscle function otherwise accompanying aging and the development of sarcopenia.

### NOX4 deficiency promotes systemic inflammation and metabolic disease

**Fig. 5.**
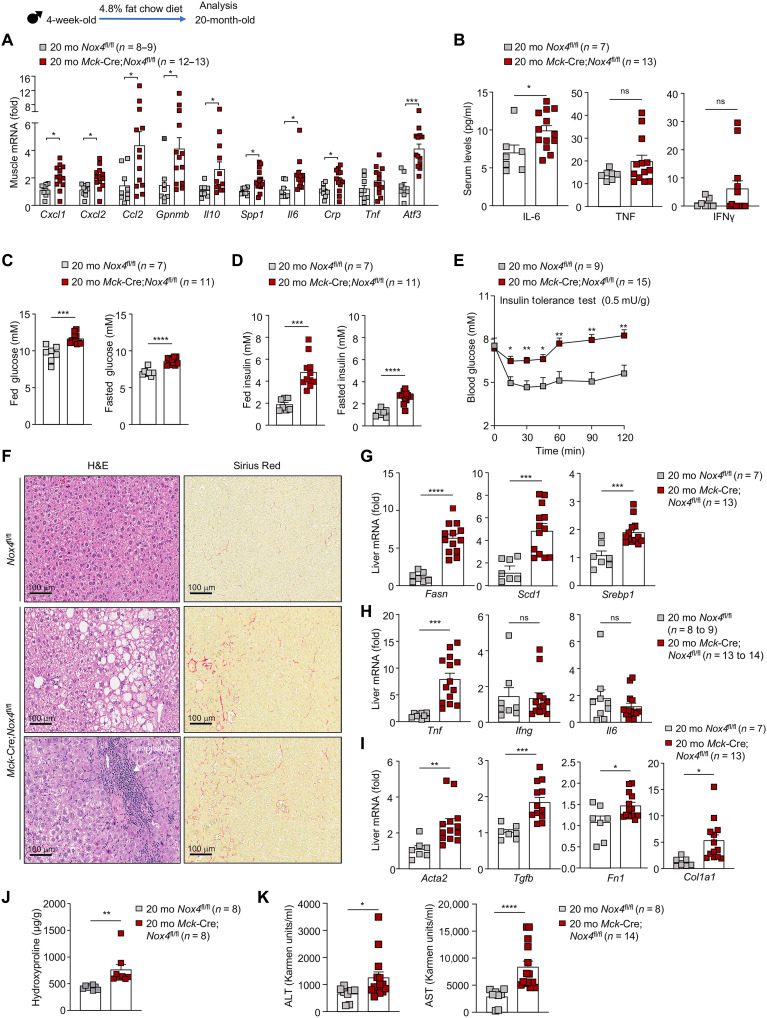
NOX4 deficiency promotes systemic inflammation and metabolic disease in aged mice. *Nox4*^fl/fl^ and *Mck*-Cre;*Nox4*^fl/fl^ male mice were fed a standard chow diet (4.8% fat) for 20 months. (**A**) Gastrocnemius muscle extracted and processed for qPCR or blood analyzed for (**B**) serum IL-6, TNF, and IFN-γ levels and fed and fasted (6 hours) (**C**) blood glucose levels and (**D**) plasma insulin levels. (**E**) Mice were subjected to insulin tolerance tests (0.5 mg insulin/g body weight). (**F**) Livers were processed for histology (H&E and Picrosirius red staining); (**G** to **I**) qPCR to monitor for (G) lipogenesis, (H) inflammation, and (I) fibrosis gene expression; or (**J**) measurement of liver hydroxyproline levels. (**K**) Serum levels of the liver enzymes ALT (alanine transaminase) and AST (aspartate transferase) were assessed. Representative and quantified results are shown (means ± SEM) for the indicated number of mice; significance was determined using a Student’s *t* test (A to D and H to K) or a two-way ANOVA (E) (see section “Quantification and statistical analysis”).

A consequence of diminished skeletal muscle NFE2L2 adaptive responses to stressors and ensuing oxidative distress during aging might be the development of senescence and inflammation (*62*–*67*). Previous studies have shown that senescence and inflammatory genes are enriched in the aged and sarcopenic muscles of mice and humans (*62*–*65*). Consistent with this, we found that the decreased muscle mass (Fig. 3C) and function (Fig. 3H) (sarcopenia) accompanying skeletal muscle NOX4 deficiency in 20-month-old mice was associated with the increased expression of senescence, inflammation, and frailty-associated genes (*Gpnmb*, *Spp1*, *Il6*, *Tnf*, *Cxcl1*, *Ccl2*, and *Crp*) (*21*, *63*, *65*, *67*) as assessed by qPCR (Fig. 5A). An outcome of decrease muscle function/sarcopenia and physical inactivity is increased adiposity, systemic inflammation, and metabolic dysfunction. Consistent with this, 20-month-old chow-fed *Mck*-Cre;*Nox4*^fl/fl^ mice had significantly increased circulating levels of the proinflammatory cytokine IL-6, with TNF and IFN-γ trending higher (Fig. 5B) and were overtly hyperglycemic (Fig. 5C), hyperinsulinemic (Fig. 5D), and insulin resistant (Fig. 5E), as reflected by increased fed and fasted blood glucose and plasma insulin levels and diminished insulin-induced glucose lowering in insulin tolerance tests (ITTs) (Fig. 5, C to E). Hyperinsulinemic-euglycemic clamps, a gold-standard measure of insulin sensitivity and glucose homeostasis, were applied to conscious and free-moving 12-month-old male wild-type and muscle NOX4-deficient mice before any difference in body weight/composition (fig. S15A) and when skeletal muscle NOX4 levels were reduced in wild-type mice. These analyses reaffirmed that NOX4-deficient mice were markedly insulin resistant (as reflected by the reduced glucose infusion rate necessary to maintain euglycemia during the insulin clamp), and this could be largely attributed to reduced glucose clearance (as reflected by the reduced rate of glucose disappearance, a measure of muscle and adipose tissue glucose uptake; fig. S15, B to D). Moreover, as might be expected from the increased adiposity and the development of whole-body insulin resistance, which would be predicted to result in both lipid flux from adipose tissue to the liver and increased liver lipogenesis, 20-month-old chow-fed *Mck*-Cre;*Nox4*^fl/fl^ mice developed metabolic dysfunction–associated fatty liver disease (MASLD) as assessed histologically by monitoring for hepatic lipid accumulation (steatosis) (Fig. 5F) and reflected by the increased liver weights in male mice (Fig. 3C). This was accompanied by the increased hepatic expression of genes involved in de novo lipogenesis, including *Fasn*, *Srebp1*, and *Scd1* (Fig. 5G). Beyond promoting MASLD, muscle NOX4 deficiency promoted the progression to the more advanced metabolic dysfunction–associated steatohepatitis (MASH), which does not otherwise occur in chow-fed mice or indeed obese C57BL/6 mice fed a high-fat diet (*41*, *68*). The progression to MASH in these mice was characterized by the presence of hepatic immune infiltrates, as assessed histologically (Fig. 5F) and the increased hepatic expression of *Tnf* (Fig. 5H); *Ifng* and *Il6* were not altered (Fig. 5H). An expected consequence of immune cell recruitment is liver inflammation, damage, and the induction of reparative processes that lead to fibrosis. Consistent with this, we found that 20-month-old chow-fed *Mck*-Cre;*Nox4*^fl/fl^ mice exhibited overt signs of liver fibrosis, as reflected by Picrosirius red staining (stains collagen; Fig. 5F), the increased hepatic expression of fibrosis-related genes, including *Acta2* and *Tgfb*, indicative of hepatic stellate cell activation, the extracellular matrix genes *Fn1* and *Col1a1* (Fig. 5I), and the significantly increased abundance of hydroxyproline (Fig. 5J), a measure of collagen degradation and the severity of fibrosis. Muscle NOX4 deficiency in 20-month-old chow-fed mice was accompanied by the development of overt liver damage, as reflected by the significantly increased presence of the liver enzymes AST (aspartate transferase) and ALT (alanine transaminase) in serum (Fig. 5K). Together, these results demonstrate that NOX4 deficiency in skeletal muscle can exacerbate the decline in muscle function and promote sarcopenia and the development of systemic metabolic disease accompanied by advanced liver disease, consistent with the onset of frailty.

### NOX4 is required for the adaptive responses to exercise that mitigate aging

**Fig. 6.**
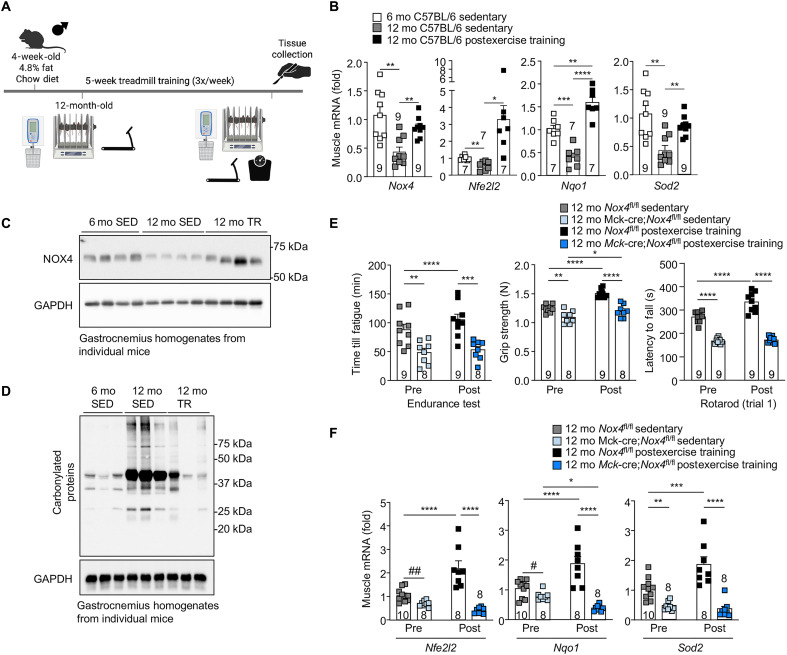
NOX4 is required for the adaptive responses to exercise. C57BL/6 mice were fed a standard chow diet (4.8% fat) for 6 or 12 months and were either exercise trained (TR) for 5 weeks (with progressively increasing running speeds each week) or left sedentary (SED). (**A**) Schematic representation of the exercise training and subsequent analyses. (**B** to **D**) Gastrocnemius muscles were extracted and processed for (B) qPCR or (C and D) immunoblotting to monitor for (C) NOX4 or (D) protein carbonylation. (**E** and **F**) *Nox4*^fl/fl^ and *Mck*-Cre;*Nox4*^fl/fl^ male mice were fed a standard chow diet (4.8% fat) for 12 months and subjected to basal and postexercise training (5 weeks) and measurements of endurance, grip strength, and motor coordination (rotarod; trial 1). (F) Alternatively, mice were either left sedentary or trained for 5 weeks and gastrocnemius muscle was extracted for qPCR. Representative and quantified results are shown (means ± SEM) for the indicated number of mice; significance was determined using a one-way ANOVA (B), two-way ANOVA (E and F), or where indicated (#) by Student’s *t* test (see section “Quantification and statistical analysis”).

Having established that skeletal muscle NOX4 and NFE2L2 adaptive responses decline with age and that NOX4 deletion in muscle exacerbates the development of sarcopenia and promotes the onset of frailty, we next sought to determine why NOX4 might decline with age. NOX4 mRNA and protein levels in skeletal muscle started to decline in C57BL/6 mice by 12 months of age (Fig. 2, A and B). At 12 months of age, the decline in NOX4 in skeletal muscle was accompanied by diminished ambulatory activity and voluntary wheel running when compared to 6-month-old mice (fig. S16). We have previously reported that NOX4 levels are induced by exercise in skeletal muscle and that this is required for the induction of NFE2L2-mediated antioxidant defense and mitochondrial biogenesis (*29*). Therefore, we determined whether the decline in NOX4 may be attributed to the decreased physical activity or otherwise represent an immutable aspect of aging. To this end, we assessed NOX4 levels and the induction of NFE2L2 antioxidant defense genes in the gastrocnemius muscle of 6-month-old versus 12-month-old sedentary or 12-month-old exercise-trained mice; trained mice were subjected to five consecutive weeks (3 days/week) of treadmill running progressively increasing intensity from 50 to 80% of their maximum pretraining exercise capacity in each successive week (Fig. 6A). We found that NOX4 mRNA and protein levels in gastrocnemius muscle in 12-month-old trained mice were reinstated to those in 6-month-old mice (Fig. 6, B and C). Moreover, we found that exercise training also increased the expression of *Nfe2l2*, *Sod2*, and *Nqo1* levels as assessed by qPCR so that they approximated or exceeded those in 6-month-old sedentary mice (Fig. 6B). In addition, exercise training ameliorated the increased oxidative damage of proteins otherwise associated with aging in gastrocnemius muscle (Fig. 6D). Therefore, the decline in NOX4 and NFE2L2 antioxidant defense in skeletal muscle can be attributed to the diminished physical activity that occurs with aging. Exercise training also enhanced muscle function and motor coordination, as reflected in treadmill endurance tests, grip strength measurements, and rotarod performance tests; however, muscle NOX4 deficiency completely abrogated such exercise-induced responses (Fig. 6E and figs. S17A and S18A). NOX4 deficiency also prevented the induction of NFE2L2 target genes (*Nfe2l2*, *Sod2* and *Nqo1*) (Fig. 6F) otherwise associated with exercise training without overtly affecting body weight or body composition (figs. S17, B and C, and S18, B and C). Therefore, these results ascribe the aging-associated decline in NOX4 to the decline in physical activity and establish that muscle NOX4 is essential for NFE2L2 adaptive responses to exercise. Moreover, our findings are consistent with the aging-associated decline in NOX4 being causal in the accompanying decline in physiological integrity.

### NFE2L2 activation tempers aging in muscle NOX4-deficient mice

**Fig. 7.**
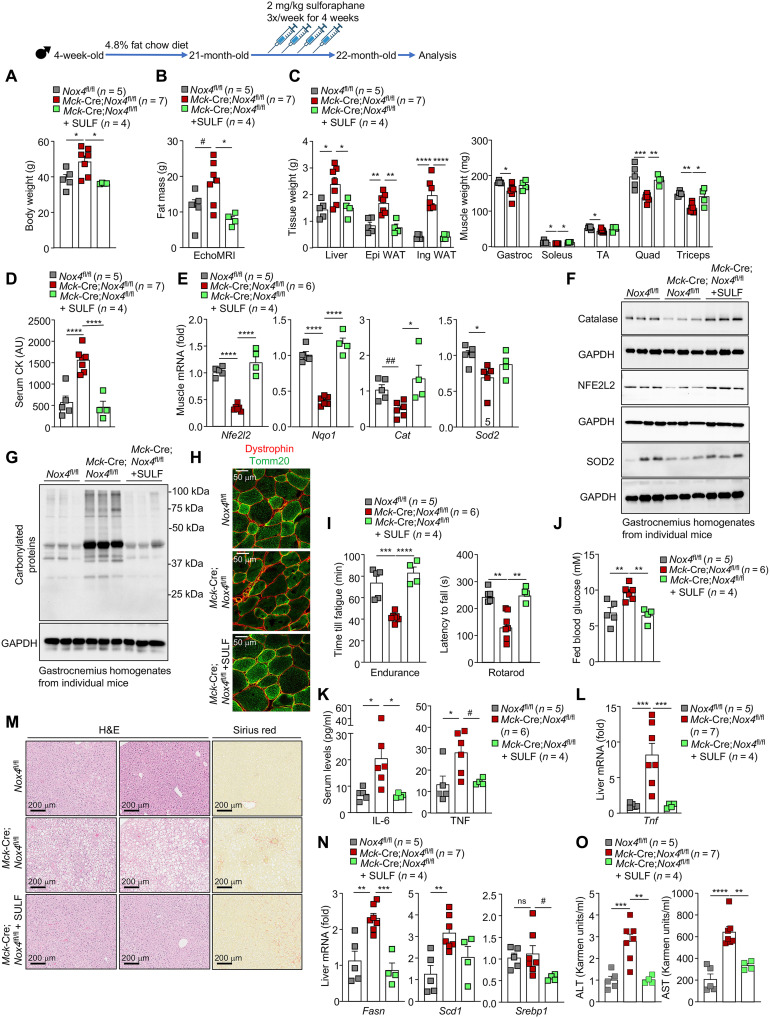
Sulforaphane treatment corrects the physiological decline and metabolic dysfunction in aged muscle NOX4-deficient mice. *Nox4*^fl/fl^ control and *Mck*-Cre;*Nox4*^fl/fl^ muscle-specific NOX4-deficient mice were fed a standard chow diet (4.8% fat) for 21 months and treated with vehicle (DMSO) or sulforaphane as indicated for 4 weeks. (**A**) Body weight, (**B**) fat mass (EchoMRI), and (**C**) tissue weights were assessed. (**D**) Serum CK levels were assessed and gastrocnemius muscles were extracted and processed for (**E**) qPCR or (**F** and **G**) immunoblotting monitoring for (F) NFE2L2, SOD2, and catalase levels or (G) protein carbonylation (Oxyblot), or (**H**) immunostaining monitoring for mitochondria (Tomm20) within muscle fibers (dystrophin). (**I**) Mice were subjected to tests for endurance or motor coordination tests (rotarod). (**J**) Fed blood glucose levels. (**K**) Serum IL-6 and TNF levels or (**L**) hepatic *Tnf* mRNA were assessed to monitor for systemic and hepatic inflammation, respectively. (**M**) Livers were processed for histology to monitor for steatosis (H&E) or fibrosis (Picrosirius red). (**N**) Livers were processed for qPCR to assess the expression of lipogenesis genes. (**O**) Serum ALT and AST levels were measured to monitor for ongoing liver damage. Representative and quantified results are shown (means ± SEM) for the indicated number of mice; significance was determined using one-way ANOVAs (A to O) or where indicated (#) by Student’s *t* test.

Physical activity and capacity progressively decline with age, and the implementation of regular exercise regimes in older people with increasing frailty and/or in people with diverse ethnic and socioeconomic backgrounds remains a significant challenge. An alternative approach may be to take advantage of NFE2L2 agonists to mimic the NFE2L2 adaptive homeostatic responses that are induced by exercise and NOX4 and decline with age. The isothiocyanate sulforaphane is a naturally occurring NFE2L2 agonist that is abundant in cruciferous vegetables and has been reported to protect skeletal muscle and other tissues from oxidative damage (*69*, *70*). Sulforaphane can directly modify Cys residues on KEAP-1 to release and stabilize NFE2L2 to promote its nuclear translocation and the expression of hundreds of NFE2L2 target genes including those driving antioxidant defense and mitochondrial biogenesis (*71*, *72*). Consistent with this, we found that, in muscle cells, the deletion of *Nfe2l2* using CRISPR-Cas9 RNP gene editing abrogated sulforaphane’s ability to not only increase NFE2L2 abundance, but also induce the expression of NQO1 that is exclusively regulated by NFE2L2 in muscle (*7*) and is important in antioxidant defense (fig. S19A), as well as the expression of other antioxidant defense genes (fig. S19B). Moreover, we have previously shown that sulforaphane can act downstream of NOX4 to promote PGC1α expression (drives mitochondrial biogenesis) and muscle function, and increase antioxidant defense and temper oxidative distress in both *Mck*-Cre;*Nox4*^fl/fl^ muscle and NOX4-deficient myoblasts (*29*). Accordingly, we determined whether sulforaphane could reverse the sarcopenia and frailty evident in our aged muscle-specific NOX4-deficient mice. Twenty-one–month-old 4.8% fat chow-fed *Mck*-Cre;*Nox4*^fl/fl^ mice were administered vehicle or sulforaphane (2 mg/kg, 3×/week, intraperitoneally) for 4 weeks and effects on body weight, muscle function, metabolic health, and NFE2L2 adaptive responses were assessed (Fig. 7). Sulforaphane reversed the increased body weight (Fig. 7A) and adiposity (Fig. 7, B and C) otherwise evident in 22-month-old vehicle-treated *Mck*-Cre;*Nox4*^fl/fl^ mice and conversely increased muscle mass to that in 22-month-old *Nox4*^fl/fl^ control mice (Fig. 7C). The improved muscle mass was accompanied by the correction of serum CK otherwise at pathological levels in 22-month-old vehicle-treated *Mck*-Cre;*Nox4*^fl/fl^ mice, to those in 22-month-old *Nox4*^fl/fl^ control mice (Fig. 7D). This was accompanied by the reinstatement of NFE2L2 orchestrated antioxidant defense, as reflected by expression of NFE2L2 target genes (*Nfe2l2*, *Nqo1*, *Cat*, and *Sod2*) (Fig. 7E) and the corresponding NFE2L2, SOD2, and catalase protein (Fig. 7F) and the attenuated oxidative damage of proteins (Fig. 7G) in gastrocnemius muscle. More importantly, sulforaphane corrected the otherwise decreased gastrocnemius muscle mitochondrial content (as assessed by histologically monitoring for Tomm20 staining) (Fig. 7H) and decreased exercise capacity and motor coordination (measured by rotarod tests) (Fig. 7I) and the overt metabolic dysfunction associated with muscle NOX4 deficiency in aged mice (Fig. 7, J to O). In particular, sulforaphane corrected the hyperglycemia (Fig. 7J), the increased hepatic (*Tnf*) and systemic inflammation (as reflected by the elevated serum TNF and IL-6 levels; Fig. 7, K and L), and the increased liver weights (Fig. 7C) and steatosis, as assessed histologically (Fig. 7M) and reflected by the repressed expression of the lipid synthesis genes *Fasn* and *Scd1* and the trending repression of *Srebp1* (Fig. 7N). Sulforaphane treatment also corrected the ongoing liver damage as reflected by the attenuated serum levels of AST and ALT (Fig. 7O) but had no impact on the extent of fibrosis (Picrosirius red staining) (Fig. 7M) that is known to be progressively irreversible. Together, these results suggest that NFE2L2 agonists can reinstate NOX4/NFE2L2 adaptive homeostatic responses in skeletal muscle to temper the physiological decline otherwise associated with physical inactivity in the aged.

### The restoration of NOX4 tempers the physiological decline with aging

**Fig. 8.**
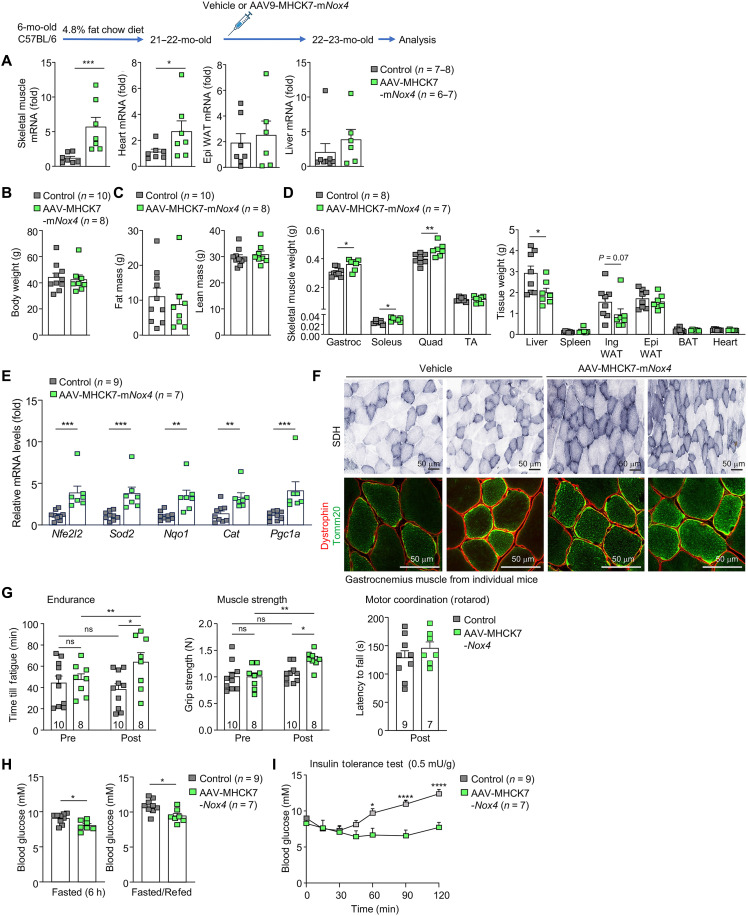
Muscle Nox4 reconstitution tempers the physiological decline in aged C57BL/6 mice. Six-month-old C57BL/6 mice were fed a standard chow diet (4.8% fat) for 15 to 16 months and then injected with vehicle or AAV9-MCHK7-m*Nox4* and analyzed after 5 weeks. (**A**) *Nox4* expression in gastrocnemius skeletal muscle, heart, liver, or Epi WAT assessed by qPCR. (**B**) Body weights, (**C**) body composition (EchoMRI), and (**D**) tissue weights [gastrocnemius (Gastroc), soleus, quadriceps (Quad), and tibialis anterior (TA) skeletal muscles; liver; spleen; inguinal (Ing) and epididymal (Epi) WAT] were analyzed. (**E** and **F**) Gastrocnemius muscle extracted and processed for (E) qPCR or (F) immunostaining to monitor for SDH to define mitochondria or Tomm20 and dystrophin to define mitochondria within individual muscle fibers. (**G**) Mice were subjected to tests for endurance, grip strength, or motor coordination (rotarod) pre- and postvehicle or AAV9-MHCK7-*mNox4* administration as indicated. (**H**) Fasted (6 hours) or overnight fasted and refed (4 hours) blood glucose levels. (**I**) Mice subjected to insulin tolerance tests (0.5 mg insulin/g body weight). Representative and quantified results are shown (means ± SEM) for the indicated number of mice; significance was determined using 2-tailed Mann-Whitney *U* test (A to E, H, and I) and two-way ANOVA (G).

In addition to stabilizing NFE2L2, sulforaphane has been reported to also affect other signaling pathways, including inhibiting inflammatory nuclear factor κB (NF-κB) signaling (*71*). Therefore, we sought to specifically restore NOX4 and the NOX4/NFE2L2 axis in the muscles of aging mice. To this end, we took advantage of adeno-associated viruses (AAVs) bearing the cardiac and muscle-specific chimeric promoter (MHCK7) that has been used previously in the context Duchene muscular dystrophy (*73*). First, we determined whether the restoration of *Nox4* in aged C57BL/6 mice might temper the physiological decline associated with aging. We administered 21- to 22-month-old chow-fed C57BL/6 male mice vehicle or AAV9-MCHK7-m*Nox4* and analyzed mice after 5 weeks (Fig. 8). In aged C57BL/6 mice, where skeletal muscle NOX4 levels otherwise decline (Fig. 2), gastrocnemius muscle *Nox4* mRNA in AAV9-MCHK7-m*Nox4* treated mice increased by 4.73-fold relative to controls (Fig. 8A) whereas *Nox4* expression in the heart increased by 2.76-fold (Fig. 8A); *Nox4* mRNA was not significantly increased in liver or adipose tissue (Fig. 8A). Although body weights (Fig. 8B) and composition (EchoMRI) were not significantly altered (Fig. 8C), skeletal muscle weights, including gastrocnemius, soleus, and quadriceps, were significantly increased in AAV9-MCHK7-m*Nox4* treated mice (Fig. 8D) and this was accompanied by decreased liver weights and a trend for reduced inguinal WAT weights (Fig. 8D). Notably, the expression of antioxidant defense and mitochondrial biogenesis genes (*Nfe2l2*, *Sod2*, *Nqo1*, *Catalase*, and *Pgc1a*; Fig. 8E) in gastrocnemius muscle was increased in AAV9-MCHK7-m*Nox4*–treated mice. This was accompanied by increased mitochondrial content (as assessed by Tomm20 staining; Fig. 8F), as well as improvements in muscle function, as reflected as by exercise capacity and grip strength (motor coordination was not corrected) (Fig. 8G) and glucose homeostasis, as reflected in the decreased hyperglycemia (Fig. 8H) and the marked improvement in insulin sensitivity in ITTs (Fig. 8I). Therefore, the functional and metabolic deficits accompanying the decline in NOX4 in aging C57BL/6 mice can be readily reversed by the reconstitution or overexpression of NOX4 in muscle.

**Fig. 9.**
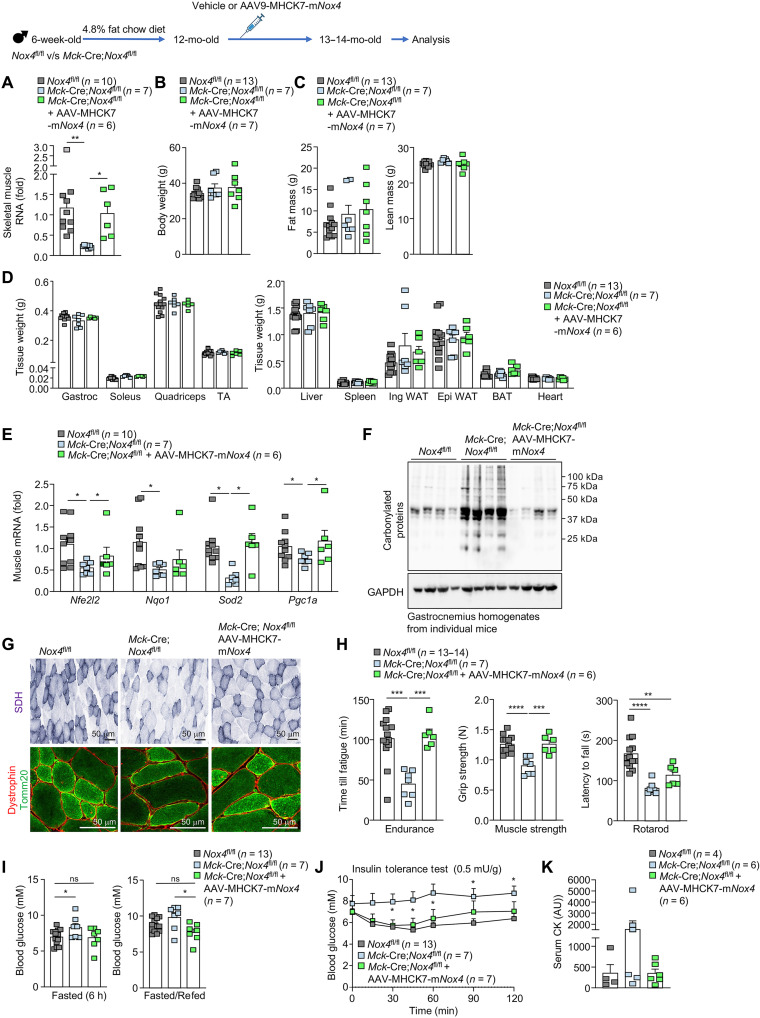
Muscle Nox4 reconstitution corrects the decreased antioxidant defense, decline in muscle function, and metabolic dysfunction in NOX4-deficient mice. *Nox4*^fl/fl^ control and *Mck*-Cre;*Nox4*^fl/fl^ muscle-specific NOX4-deficient mice were fed a standard chow diet (4.8% fat) up to 12 months of age and *Mck*-Cre;*Nox4*^fl/fl^ mice administered (intravenous) vehicle or AAV9-MCHK7-m*Nox4* and analyzed after 5 weeks. (**A**) *Nox4* expression in gastrocnemius skeletal muscle assessed by qPCR. (**B**) Body weights, (**C**) body composition (EchoMRI), and (**D**) tissue weights [gastrocnemius (Gastroc), soleus, quadriceps (Quad), and tibialis anterior (TA) skeletal muscles; liver; spleen; inguinal (Ing) and epididymal (Epi) WAT] were analyzed. Gastrocnemius muscles were extracted and processed for (**E**) qPCR, (**F**) immunoblotting to assess protein carbonylation, or (**G**) immunostaining to monitor for SDH to define mitochondria or Tomm20 and dystrophin to define mitochondria within individual muscle fibers. (**H**) Mice were subjected to tests for endurance, grip strength, or motor coordination (rotarod). (**I**) Fasted (6 hours) or overnight fasted and refed (4 hours) blood glucose levels. (**J**) Mice were subjected to insulin tolerance tests (0.5 mg insulin/g body weight). (**K**) Serum CK levels. Representative and quantified results are shown (means ± SEM) for the indicated number of mice; significance was determined using one-way ANOVA (A to E and H) or Student’s *t* test (I and J).

Next, we also sought to re-express *Nox4* in the skeletal muscles of 12-month-old *Mck*-Cre;*Nox4*^fl/fl^ mice (Fig. 9). We administered 12-month-old *Nox4*^fl/fl^ and *Mck*-Cre;*Nox4*^fl/fl^ mice fed a 4.8% fat chow diet vehicle or AAV9-MCHK7-m*Nox4* and analyzed mice after 5 weeks. Skeletal muscle *Nox4* levels in *Mck*-Cre;*Nox4*^fl/fl^ versus *Nox4*^fl/fl^ mice were largely ablated (>80% reduction) (Fig. 9A). In *Mck*-Cre;*Nox4*^fl/fl^ mice administered AAV9-MCHK7-m*Nox4*, gastrocnemius muscle *Nox4* levels were reconstituted to those in *Nox4*^fl/fl^ controls (Fig. 9A). In 12-month-old *Nox4*^fl/fl^ and *Mck*-Cre;*Nox4*^fl/fl^ mice, no differences were evident in body weights (Fig. 9B), body composition (Fig. 9C), or tissue weights (Fig. 9D), and this was not altered in mice administered AAV9-MCHK7-m*Nox4*. However, the expression of antioxidant defense and mitochondrial biogenesis genes (*Nfe2l2*, *Sod2*, *Nqo1*, and *Pgc1a*) was reduced in *Mck*-Cre;*Nox4*^fl/fl^ mice (Fig. 9E) and accompanied by increased oxidative damage (protein carbonylation; Fig. 9F), a reduction in mitochondrial content (as reflected by subsarcolemmal and intermyofibrillar SDH and Tomm20 staining; Fig. 9G), decreased muscle function (as assessed by measuring exercise capacity, grip strength, and motor coordination in rotarod tests; Fig. 9H), metabolic dysfunction (hyperglycemia and insulin resistance as assessed in ITTs; Fig. 9, I and J), and signs of overt muscle damage as reflected in increased serum CK levels (Fig. 9K). Notably, the muscle-specific reconstitution/overexpression of *Nox4* largely reversed the diminished skeletal muscle antioxidant defense gene expression (Fig. 9E) and increased protein carbonylation (Fig. 9F). *Nox4* reconstitution/overexpression also corrected the reduced mitochondrial content (Fig. 9G) and muscle function (as reflected by the corrected exercise capacity and grip strength; motor coordination trended higher; Fig. 9H) as well as the overt metabolic dysfunction (Fig. 9, I and J) and muscle damage (Fig. 9K). Together, our studies indicate that abrogated NOX4/NFE2L2-orchestrated adaptive homeostatic responses accompanying physical inactivity during aging can be corrected by the muscle reconstitution/overexpression of NOX4 to temper the physiological decline during aging.

## DISCUSSION

The evidence supporting the importance of physical activity in promoting resilience and healthy aging is irrefutable (*23*). Lifelong physical inactivity in mammals can accelerate the loss in bone density, skeletal muscle mass, muscle strength, and power, otherwise associated with aging, whereas physical activity builds resilience and fitness (*74*, *75*). The results of this study are consistent with the benefits of physical activity/exercise in promoting healthy aging being at least in part attributed to NOX4-dependent skeletal muscle ROS generation for the induction of NFE2L2-orchestrated adaptive homeostatic responses that among other things mitigate macromolecular oxidative damage and perturbations in mitochondrial function. Our studies indicate that a diminution in physical activity with age results in a decline in skeletal muscle NOX4 levels, abrogating NFE2L2 adaptive homeostasis, thereby resulting in compromised muscle function, increased oxidative macromolecular damage, and the progressive development of sarcopenia and frailty. Our studies suggest that the physiological decline associated with physical inactivity and abrogated NOX4/NFE2L2 adaptive homeostasis can be corrected by the overexpression/reconstitution of NOX4 in muscle, or the administration of NFE2L2 agonists.

Previous studies have shown that both cardiac and skeletal muscle NOX4 levels are induced in response to exercise training and/or acute exercise in mice (*29*, *34*). Skeletal muscle *Nox4* mRNA is also induced after acute high-intensity interval training in humans (*29*). NOX4 is a transcriptional target of NFE2L2 and generates H_2_O_2_ as part of a feed forward loop to drive NFE2L2 adaptive responses (*29*, *40*, *41*). Although the precise mechanisms governing NOX4 regulation in contracting skeletal muscle remain unclear, we speculate that the activation of NFE2L2 by alternate means, possibly involving p62/SQSTM (*7*, *10*), may be required for the initiation of this feed forward loop. Irrespective, the induction of NOX4 and the resultant ROS/H_2_O_2_ generation activate/stabilize NFE2L2 to promote antioxidant defense and mitochondrial biogenesis to (i) enhance cardiac or muscle function and exercise capacity; (ii) mitigate the oxidative damage of macromolecules, including proteins and lipids; and (iii) temper the mitochondrial oxidative distress and the decline in insulin sensitivity that otherwise occurs with aging (*29*, *34*). Yet, other studies suggest that NOX4 in the vasculature may also facilitate exercise capacity by promoting glucose and fatty acid oxidation (*76*) or by increasing capillary density in response to exercise (*77*, *78*) and that NOX4 may also protect the vasculature from ischemic or inflammatory stress (*77*). Recently, we have also shown that heightened hepatocyte NOX4 levels in MASLD similarly induce NFE2L2 adaptive responses in hepatocytes to limit oxidative damage, metabolic dysfunction, and the progression from simple steatosis to the more advanced MASH with fibrosis (*41*). Herein, we have shown that skeletal muscle NOX4, NFE2L2, and downstream transcriptional targets that mediate antioxidant defense decline in abundance in aged mice and humans and that this is accompanied by the oxidative damage of proteins. By contrast, we noted that cardiac *NOX4* levels and NFE2L2 target genes are unaltered during aging, which is not unexpected, because the decline in the NOX4/NFE2L2 axis in skeletal muscle was linked to physical inactivity. In mice, we have shown that skeletal muscle NOX4 mRNA and protein levels start to decline by ~12 months of age when locomotor activity and voluntary wheel running decline, and this coincides with diminished NFE2L2 antioxidant defense and conversely a marked increase in oxidative protein damage that progressively worsens with age. By contrast, we have previously reported that the NOX2 catalytic subunit is not reduced in skeletal muscle in aged mice (*29*), whereas in this study we have shown that NOX2 protein levels do not decline in the skeletal muscles of aged men. We show that the decline in NOX4 is causally linked to the decline in NFE2L2-orchestrated adaptive homeostasis in several ways. First, the deletion of NOX4 in a human immortalized myoblast cell line and/or in hiPSC-derived myoblasts is accompanied by the reduction in NFE2L2 abundance and nuclear localization; a decline in the abundance of key NFE2L2 targets and antioxidant defense enzymes, including SOD2, PRDX3, NQO1, and catalase; increased mitochondrial distress (as reflected by MitoSOX detection of O2•^−^); and a marked increase in the oxidative damage of proteins (protein carbonylation). Second, we show that the additional deletion of *KEAP1* in NOX4-deficient muscle cells increases the abundance and nuclear localization of NFE2L2 and corrects the decline in antioxidant defense and oxidative distress. Third, we show that whereas skeletal muscle NOX4, NFE2L2, and key NFE2L2 downstream targets decline by 12 months of age, these can be rescued by exercise training in wild-type mice, but not in muscle NOX4-deficient mice. Fourth, we show that adaptive homeostatic responses induced by exercise, including enhanced running capacity, skeletal muscle strength (grip strength), and motor coordination (rotarod test), are completely abrogated by muscle NOX4 deficiency. Although we previously reported an ~30% reduction in heart *Nox4* mRNA in 3-month-old *Mck*-Cre;*Nox4*^fl/fl^ mice, this was not accompanied by changes in cardiac H_2_O_2_ levels or heart function (*29*). Moreover, cardiac *Nox4* mRNA levels are not reduced in 12-month-old *Mck*-Cre;*Nox4*^fl/fl^ mice, making it unlikely that the decline in exercise-induced NOX4/NFE2L2 adaptive homeostasis and skeletal muscle function in aging mice could be attributed to NOX4 deficiency in the heart. Last, we show that whereas muscle NOX4 deficiency exacerbates the age-associated decline in skeletal muscle NFE2L2 antioxidant defense, oxidative damage of proteins, and the development of sarcopenia/frailty, these can be largely, if not completely corrected by the muscle-specific reconstitution/overexpression of NOX4 with AAVs, or by the administration of the NFE2L2 agonist sulforaphane. Notably, we show that the reconstitution of NOX4 in skeletal muscle and overexpression in the heart using AAVs was sufficient to not only correct the decline in NFE2L2 adaptive responses and physiological integrity in 12-month-old NOX4-deficient mice, but also increase skeletal muscle mass, mitochondrial content, and muscle function, as well as improve glucose homeostasis in 22- to 23-month-old aged C57BL/6 mice. Because previous studies have shown that, beyond its role in skeletal muscle (*29*), the induction of NOX4 in cardiac muscle after acute exercise also enhances exercise capacity (*34*), we cannot exclude that the NOX4 overexpression in the heart may have also contributed to the improved muscle function and glucose metabolism in mice treated with *Nox4*-expressing AAVs. Nonetheless, together, our studies ascribe the increasing oxidative damage of macromolecules in skeletal muscle, worsening skeletal muscle function, and the overall decline in physiological integrity that accompany physical inactivity during aging to the decline in NOX4-instigated and NFE2L2-orchestrated adaptive homeostasis.

Although we have focused on NFE2L2-orchestrated antioxidant defense in this study, the decline in physical activity and NOX4 undoubtedly affects numerous other NFE2L2-orchestrated adaptive responses during aging. Our previous studies have shown that skeletal NOX4 deficiency reduces mitochondrial biogenesis and content and compromises exercise capacity and endurance (*29*). Consistent with this, others have shown that global NOX4 deficiency attenuates the induction of PGC1α and mitochondrial biogenesis induced by voluntary wheel running (*79*). Similarly, in this study, we show that muscle NOX4 deficiency in aged mice exacerbates the reduction in PGC1α and mitochondrial content associated with physical inactivity during aging, consistent with a decline in mitochondrial biogenesis. Conversely, muscle NOX4 reconstitution/overexpression in aged C57BL/6 mice increased skeletal muscle mitochondrial content. The induction of mitochondrial biogenesis is a fundamental response to exercise and is considered essential for the promotion of respiratory capacity and endurance (*24*, *59*). NFE2L2 is required for the induction of mitochondrial biogenesis after exercise and for optimal exercise performance (*12*, *59*, *69*, *70*). Previous studies have shown that the global deletion of NFE2L2 results not only in the oxidative damage of macromolecules in the muscles of aged mice (*36*) but also in the decline of mitochondrial content and the onset of sarcopenia and frailty (*14*). Several studies have shown that skeletal muscle mitochondrial capacity and/or mitochondrial biogenesis and ETC complex genes decline in aged individuals; however, this can be attenuated at least in part by increasing or maintaining physical activity during aging (*35*, *66*, *80*–*83*). Consistent with this, we found that even in aged C57BL/6 mice, or aged NOX4-deficient mice with overt sarcopenia and frailty, the muscle-specific restoration/overexpression of NOX4 or the administration of the NFE2L2 agonist sulforaphane to bypass NOX4 deficiency increased muscle mass, mitochondrial content, and physical activity to mitigate sarcopenia and frailty.

Beyond the promotion of oxidative distress and reduced mitochondrial content, NOX4 deficiency in aged mice additionally promoted local inflammation, as reflected by the increased expression of both senescence and inflammatory genes in skeletal muscle. This may be an outcome of mitochondrial dysfunction and oxidative distress or otherwise the reduced PGC1α that normally inhibits the expression of proinflammatory genes such as TNF and IL-6 (*11*, *37*). In addition, the decline in NFE2L2-may also contribute, because NFE2L2 can directly repress NF-κB expression as well as the expression of proinflammatory cytokines such as IL-6 and IL-1β in a cell context–dependent manner (*11*, *37*). Alternatively, it is possible that the decline in NOX4/NFE2L2-orchestrated adaptive responses and resultant oxidative distress in multinucleated muscle cells may affect resident mononuclear cells, including muscle stem cells, mesenchymal progenitors, T cells, and macrophages that are important for muscle homeostasis, repair, and exercise responses (*42*–*44*, *84*, *85*) and whose activation and relative abundance in skeletal muscle changes in aged mice to increase the expression of proinflammatory and senescence-related markers (*63*, *65*). Irrespective, we propose that the local skeletal muscle inflammation likely contributes to the systemic inflammation and the associated development of both muscle wasting and insulin resistance evident in our NOX4-deficient mice (*85*, *86*).

Previous studies have reported that skeletal muscle aging is associated with markers of oxidative damage in muscle tissue and in isolated muscle fibers (*87*–*89*). Yet, other studies suggest that skeletal muscle aging in mice is associated with a fundamental remodeling of the redox landscape that involves the increased oxidation of proteins linked to muscular dystrophies (*90*). A fundamental question that arises from our studies is how can the decline in NOX4-derived ROS contribute to oxidative distress and the increased oxidation of specific redox networks? One possibility is that this may be attributed to the compartmentalization of ROS generation during aging. Studies have shown that mitochondrial, but not cytosolic H_2_O_2_ may be increased in skeletal muscle fibers and in isolated mitochondria from old mice (*16*, *91*, *92*). By contrast, NOXs, particularly NOX2 and NOX4, rather than mitochondria, are responsible for the beneficial exercise-induced H_2_O_2_ generation required for adaptive responses (*26*, *29*, *30*, *93*). Consistent with this, we have previously shown that the deletion of NOX4 in myoblasts and the resultant reduction in H_2_O_2_ are accompanied by increased mitochondrial ROS generation and the oxidative damage of proteins that can be corrected by activating or stabilizing NFE2L2, or by administering mitochondrial-targeted antioxidants (*29*). Moreover, we have previously shown that skeletal muscle NOX4 deficiency in 6-month-old mice was accompanied by marked reductions in both SOD2 and PRDX3 in mitochondria and increased macromolecular damage (*29*). Our current studies indicate that SOD2 is similarly reduced in the skeletal muscles of aged mice and that this is reinstated by exercise training or exacerbated by the deletion of NOX4. Moreover, we show that as in murine myoblasts, the deletion of NOX4 in human muscle cells is accompanied by reductions in SOD2, mitochondrial oxidative distress, and the marked oxidative damage of proteins. Numerous studies have shown that mitochondrial oxidative stress and the ensuing oxidative damage contribute to the development of insulin resistance (*36*, *38*, *94*–*97*) and that *Sod2* heterozygosity in mice is sufficient to promote mitochondrial oxidative stress and insulin resistance whereas SOD2 overexpression attenuates insulin resistance (*38*). Accordingly, we propose that the decline in NOX4-instigated and NFE2L2-orchestrated antioxidant defense with age, which is exacerbated by the deletion of NOX4, results in the decline in SOD2 and PRDX3 levels to promote oxidative distress and damage and the ensuing insulin resistance. The decreased mitochondrial content, physical activity, and the resultant increased adiposity associated with the decline in NOX4 that are also exacerbated by NOX4 deficiency undoubtedly also contribute to the accompanying systemic disease. However, the mechanisms that may contribute to the unexpected progression from simple steatosis to MASH and fibrosis in our aged muscle NOX4-deficient mice remain unclear. Although this may be ascribed to the accompanying systemic inflammation, other factors, including, for example, changes in myokine/exerkine secretion (*98*), may also contribute, but this requires further investigation.

Our studies suggest that the promotion of NOX4 expression in skeletal muscle in the aged may help mitigate sarcopenia and frailty. However, it is important to recognize that the overexpression or induction of NOX4 in the context of disease may paradoxically also contribute to oxidative distress and exacerbate pathology. For example, although NOX4 drives adaptive responses in the liver to attenuate MASLD progression (*41*) and its overexpression can temper disease progression in mice fed a MASH/fibrosis promoting diet (*41*), its up-regulation in the context of MASH has also been reported to exacerbate disease progression (*99*). Similarly, NOX4 is induced in the failing heart and NOX4 deletion attenuates whereas transgenic NOX4 overexpression exacerbates cardiac hypertrophy, fibrosis, and cardiomyocyte apoptosis under conditions of pressure overload induced hypertrophy (*47*, *100*). Despite this, our studies showed that the reconstitution or overexpression of NOX4 in muscle in aging muscle-specific NOX4-deficient mice, or in aged C57BL/6 mice, mitigated the decline in muscle function, the development of sarcopenia (muscle wasting), and signs of frailty. Nonetheless, given the potential for deleterious effects in advanced diseased states, we also sought to activate NFE2L2 downstream of NOX4 to reinstate the beneficial exercise-induced NFE2L2 adaptive responses that otherwise decline with aging. Our studies show that the naturally occurring NFE2L2 agonist sulforaphane can bolster antioxidant defense and completely correct muscle wasting, increased adiposity, and diminished muscle function, as well as temper the metabolic perturbations including MASLD, in aged muscle NOX4-deficient mice. Consistent with this, previous studies have shown that the administration of sulforaphane to 21- to 22-month-old C57BL/6 mice over 3 months enhances muscle function, increases antioxidant defense, and improves glucose homeostasis (*101*), whereas the administration of sulforaphane as a concentrated broccoli sprout extract once daily for 12 weeks to patients with obesity with dysregulated type 2 diabetes (HbA1c 57.1 ± 6.6 mmol/mol) on metformin lowers HbA1c levels without adverse effects (*102*). Beyond stabilizing NFE2L2, sulforaphane has been proposed to also elicit effects on many other signaling mediators, including NF-κB (*71*). However, the extent to which such effects are reliant on NFE2L2, which can directly or indirectly affect the expression of hundreds and potentially thousands of genes, remains to be determined (*71*, *72*, *103*). We showed that the deletion of *Nfe2l2* abrogated the ability of sulforaphane to increase antioxidant defense in muscle cells. Therefore, the ability of sulforaphane to at least mitigate oxidative damage in NOX4-deficient mice may be attributed to the specific activation of NFE2L2. Consistent with this, we found that the reconstitution of NOX4 that acts upstream of NFE2L2 recapitulated many of the beneficial effects of sulforaphane. Irrespective, together, our studies are consistent with the activation of NOX4/NFE2L2 axis being beneficial for tempering sarcopenia and frailty, especially in aged and physically inactive individuals, where exercise may not be readily feasible or practical.

Together, the results of this study reaffirm the fundamental importance of NOX4 in the beneficial adaptive responses induced by physical activity/exercise and demonstrate that physical inactivity and declining NOX4 levels with age are instrumental in abrogating NFE2L2-orchestrated adaptive homeostatic responses that otherwise mitigate the development of sarcopenia and frailty. Our studies provide insight into fundamental mechanisms contributing to the physiological decline with aging and define an approach for reinstating otherwise abrogated adaptive homeostatic responses that promote healthy aging.

## MATERIALS AND METHODS

### Materials

Mouse α-tubulin (catalog no. T5168, RRID:AB_477579), α-vinculin (catalog no. V9131, RRID:AB_4776298), and rabbit α-NFE2L2 (catalog no. AV38745, RRID:AB_1854419) used for immunoblotting were from Sigma-Aldrich (St. Louis, MO); mouse α-NQO1 (catalog no. NB200-209, RRID:AB_10002706) was from Novus Biologicals (Littleton, CO) and sheep α-NQO1 was from R&D Systems (Minneapolis, MN); α-catalase (catalog no. 1877, RRID:AB_302649), α-PRDX3 (catalog no. AB222807), α-NOX4 (catalog no. ab133303, RRID:AB_11155321), and rodent anti-OXPHOS cocktail (ab110413, RRID:AB_2629281) were from Abcam (San Francisco, CA); mouse α-GAPDH (catalog no. AM4300, RRID:AB_2536381) was from Thermo Fisher Scientific (Waltham, MA); rabbit α-catalase (catalog no. ab1877, RRID: AB_302649), α-NFE2L2 (catalog no. sc365949, RRID:AB_10917561) for immunoblotting, and α-NOX4 (catalog no. sc-30141, RRID:AB_2151703) were from Santa Cruz Biotechnology (Santa Cruz, CA); and α-SOD2 (catalog no. 13141, RRID:AB_2636921) and rabbit α-NFE2L2 (catalog no. 12721T, RRID:AB_2715528) for immunofluorescence microscopy were from Cell Signaling Technology (Beverly, MA). The human NOX2/gp91phox antibody (ab808897, Abcam, San Francisco, CA) was provided by S. Selemidis (RMIT University, Melbourne, Australia). Goat α-rabbit IgG (AQ132P) and goat α-mouse IgG (AQ502P) secondary antibodies for immunoblotting were purchased from Merck Millipore (Burlington, MA, USA) and donkey α-sheep IgG (HAF016) was purchased from BIotechne (Minneapolis, USA). Mouse α-dystrophin (ab7164), rabbit α-dystrophin (ab15277), and rabbit α-Tomm20 (ab78547) used for tissue immunofluorescence microscopy were from Abcam (San Francisco, CA); mouse α-myosin heavy chain I (BA-f8), IIa (SC-71), IIb (BF-F3), and IIx (6H1) were from Developmental Studies Hybridoma Bank (all fiber-typing antibodies were developed by S. Schiaffino (Universita degli Studi di Padova, Italy) or C. Lucas (University of Sydney, Australia) and obtained from the Developmental Studies Hybridoma Bank, created by the NICHD of the NIH and maintained at Department of Biology, The University of Iowa (Iowa City, IA 52242). The Mouse GDF-15 ELISA Kit (ab216947), Hydroxyproline Assay Kit (ab222941), Mouse ALT ELISA Kit (ab282882), Mouse AST ELISA Kit (ab263882), and OxyBlot Protein Oxidation Detection Kit (S7150) were from Abcam (Cambridge, UK); Rat/Mouse FGF-21 Quantikine ELISA Kit (EZRMFGF21-26K) and Citrate Synthase Assay Kit (MAK116-1KT) were from Merck Millipore (Burlington, MA, USA); and MitoSOX Red was from Invitrogen (Thermo Fisher Scientific, Waltham, MA). Legendplex assays [Mouse IL-6 Flex Set (B4) (558301), Mouse TNF Flex Set (C8) (558299), and Mouse IFN-γ Flex Set (558296)] were purchased from BD Biosciences (New Jersey, USA), and AAV-MHCK7-m*Nox4* (serotype 9; VB220718-1389zvp) was from VectorBuilder (Chicago, IL).

### Mice

Age-matched male and female murine models were used in all experimental conditions. Animals were housed under a controlled 12-hour light-dark cycle in a high-barrier, temperature-regulated facility (Monash Animal Research Laboratory) with unrestricted access to food and water. Mice were fed a standard chow diet (20% protein, 4.8% fat, and 4.8% crude fiber; Specialty Feeds). *Nox4*^fl/fl^ and *Mck*-Cre;*Nox4*^fl/fl^ have been described in previous studies (*29*, *47*). All mice were on a C57BL/6 background. C57BL/6J mice were purchased from Monash Animal Research Platform or Walter and Eliza Hall Institute of Medical Research (WEHI). Where indicated, 12-month-old *Mck*-Cre;*Nox4^fl/fl^* or 21- to 22-month-old C57BL/6J mice were injected with AAV-MHCK7-m*Nox4* (serotype 9; VB220718-1389zvp, VectorBuilder, Chicago, IL) to overexpress *Nox4* in muscle. Mice were injected intravenously with vehicle or 1 × 10^11^ genome copies of AAV-MHCK7-m*Nox4* (serotype 9) per mouse, and mice then subjected to exercise studies, muscle performance tests, and metabolic measurements after 5 weeks before tissues were extracted for analysis. Experiments were approved by the Monash University School of Biomedical Sciences Animal Ethics Committee (14368, 22138, and 36631) and were carried out in accordance with the NHMRC Australian Code of Practice for the Care and Use of Animals. All animals were aged to 3 to 24 months for the different aging timeline studies.

### Human participants and biopsies

For immunoblotting, vastus lateralis muscle from overnight fasted young (27.6 ± 6.6 years old, *n* = 10) and aged (69.9 ± 3.1 years old, *n* = 10) men that were matched for body mass index (BMI_young_ = 24.3 ± 0.2; BMI_aged_ = 22.9 ± 0.1) and had active lifestyles have been described previously (*104*, *105*) and conformed to the West Midlands—Black Country Research Ethics Committee (#17/WM/0068) and the Swedish Ethical Review Authority (#2017/1139-31/44; #2017/2107–31/2) and adhered to the principles set by the Declaration of Helsinki.

For the proteomic analyses of human mitochondrial fractions, 12 patients (6 young adults and 6 older adults) undergoing trauma or orthopedic surgery were included in this study. Their medical and functional status was evaluated using the modified five-item Frailty Index (*106*). Exclusion criteria encompassed conditions such as myopathy, hemiplegia or hemiparesis, rheumatoid arthritis or other autoimmune connective tissue disorders, inability to provide consent, hospital admission within the past month, or major surgery in the preceding 3 months. Each participant provided written informed consent following a comprehensive explanation of the study’s purpose and potential risks. This research adhered to the principles outlined in the Declaration of Helsinki and received ethical approval from the Ethical Committee for Drug Research of the Department of Health Arnau de Vilanova—Liria, Spain (license reference: CEIm 28/2019). Skeletal muscle samples were collected during surgery from a healthy muscle region, ensuring the absence of contusion or hematoma. A small portion of the muscle was excised using a scalpel, following the natural alignment of the muscle fibers while avoiding electrocautery. The samples were immediately frozen in liquid nitrogen for subsequent mitochondrial isolation and proteomic analyses.

### Rodent exercise studies

Mice were acclimatized to treadmill running for 3 days before the initiation of the experiments. Mice were placed on a multilane treadmill (Columbus Instruments, Columbus, OH) for 10 min and run for 5 min at 10 m/min and 1 min at 15 m/min at 0% slope. All animals were randomized before the initiation of exercise tests.

#### Exercise stress test

To assess peak oxygen consumption (VO_2 peak_) male mice were placed in an enclosed single lane treadmill connected to Oxymax O_2_ and CO_2_ sensors (Columbus Instruments, Columbus, OH). On the experimental day, before the initiation of the exercise stress test, mice were acclimated in the chamber at rest for 20 min (basal O_2_ consumption and CO_2_ generation assessed for the last 5 min). The mice were then subjected to running at 10 m/min at 0% incline and the running speed (*U*) was increased by 4 m/min every 3 min until the mice reached fatigue; fatigue was determined as the time point where the mice could not be prompted to continue running for at least 5 s. O_2_ consumption (VO_2_ max) was assessed at the maximal exercise speed (*U*_max_). Differences between the exercised and basal states were calculated and gas exchange data were used to determine energy expenditure, heat, and the RER (RER = VCO_2_/VO_2_).

#### Endurance test

Mice were exercised on a multilane treadmill (Columbus Instruments International) at 0% slope. The endurance was performed with a warmup period of 10 min at 10 m/min, followed by running at 60% of the maximal exercise speed (*U*_max_) until fatigue was reached. The time till exhaustion was considered the representative measure of endurance capacity.

#### Exercise training

Mice were allocated into sedentary and training groups. The training group underwent exercise training for 5 weeks, 3 days per week, while the sedentary group remained sedentary on the treadmill for the same period of time. Mice were subjected to a gradual overload protocol, in which running speed was increased so that by the end of training weeks 1, 2, 3, 4, and 5, mice were running for up to 45 min at 50, 60, 70, 80, and 90% of their maximum pretraining speed, respectively. The maximal exercise speed and VO_2_ max were assessed using exercise stress tests.

### Muscle performance tests

#### Grip strength measurement

Limb grip strength was assessed using a grip strength meter (Bioseb, EB Instruments, France). Mice were acclimatized for 3 days by placing all four limbs on the grip strength meter grid, three times per day. The acclimatization was separated by 3 days from the experimental period. On the experimental day, mice were placed over the meter so that all paws grasped the grid while mice were handled by their tails and tails were pulled horizontally until mice released their hold of the grid. Three independent measurements were undertaken and limb strength was measured in newtons.

#### Rotarod test

Mice were trained once per day for 4 days on a rotating rod with a lane width of 5 cm (Ugo Basile Rota-Rod 476000, France) spinning at 4 rpm for 5 min. After training, mice were subjected to an incremental protocol, where the speed was increased over 480 s from 4 to 60 rpm. All animals were subjected to four independent trials separated by 1 hour, and the latency to fall (length of time that the mice remained on the rod) was recorded and analyzed (the averaged results from four trials are shown).

### Metabolic and blood measurements

Mouse body weights were monitored weekly, and body composition was evaluated using EchoMRI (Echo Medical Systems, Houston, TX), as previously described (*29*). Insulin and glucose tolerance tests were carried out following established protocols (*29*). Blood samples were collected from conscious mice in both fed state (satiated, 11 p.m.) and following a 6-hour fasting period via submandibular bleeding. Plasma insulin concentrations were quantified using the Mouse Insulin ELISA (ALPCO, Salem, 80-INSMS- E01), while corresponding blood glucose levels were measured using an Accu-Chek glucometer.

Energy balance parameters, including food intake, voluntary wheel running activity, and energy expenditure, were continuously recorded over a 72-hour period following a 24-hour acclimatization phase. These assessments were performed using the Promethion Metabolic Screening System (Sable Systems International, NV), equipped with indirect open-circuit calorimetry, running wheels, and automated sensors for quantifying food consumption and locomotor activity.

Hyperinsulinemic-euglycemic clamp studies were conducted as previously described (*29*). Mice were anesthetized using 2% (v/v) isoflurane (250 ml/min O_2_), and surgical catheterization of the left common carotid artery and right jugular vein was performed to enable arterial blood sampling and intravenous infusions. On the day of the experiment, food was withdrawn, and following a 3.5-hour fasting period, a primed (2 min, 0.5 μCi/min) continuous infusion (0.05 μCi/min) of [3-^3^H]-glucose was administered to assess basal glucose turnover. After 5 hours of fasting, a continuous insulin infusion (4 mU kg^−1^ min^−1^) was initiated to achieve a hyperinsulinemic state, while blood glucose concentrations were maintained at euglycemic levels through a dynamic infusion of a 50% (w/v) glucose solution. Arterial blood samples were collected at baseline and at steady-state time points (80, 90, 100, 110, and 120 min). At the end of the experiment, mice were euthanized, and tissues were rapidly harvested, flash-frozen in liquid nitrogen, and stored for downstream analyses of gene expression and glucose uptake.

### Frailty assessment: Valencia score

The “Valencia score” was used to assess frailty in 20-month-old *Mck*-Cre;*Nox4*^fl/fl^ mice (*60*). This approach was adapted from the frailty criteria originally developed for humans by Fried *et al*. (*107*). The score includes the measurement of five key components: unintentional weight loss (for which we modified the original protocol and used lean mass loss as the criterion), reduced activity levels (motor coordination), weakness (grip strength), poor endurance (total running time in the incremental treadmill test), and slowness (maximum running speed in the incremental treadmill test). For running time, running speed, and grip strength parameters, we classified as frail the 20% of mice with the lowest performance. This allowed us to determine the percentage of mice considered frail for each parameter. For the body weight criterion, mice were classified as frail if they lost more than 5% of their lean mass compared to the previous measurement. In the case of motor coordination, mice that failed the test were considered frail for this parameter. To calculate the Valencia score of frailty, which provides an overall frailty assessment based on the five parameters, we divided the total number of failed tests by the total number of tests performed, resulting in the percentage of mice classified as frail. Statistical differences were assessed using the chi-square test.

### Cell culture

hiPSCs were obtained from the Gibco Human Episomal iPSC line (iPSC6.2, from WiCell Bank) (*108*) and differentiated into skeletal muscle cells following a modified, previously described protocol (*109*). Cells were maintained on a hiPSC-qualified Matrix (BD Matrigel, BD Biosciences, NJ) in the presence of mTeSR Plus medium (Stem Cell Technologies, Vancouver, Canada) in 10-cm Matrigel-Matrix Growth Factor Reduced (Corning, Bio-strategy catalog no. BDAA354230) coated dishes at 37°C with 5% O_2_ and 5% CO_2_. When the colony size reached >600 μm (0.6 mm) in diameter and the colony density on the plate was approximately 30 to 40%, differentiation was induced by switching the culture medium to a myogenic differentiation medium composed of a chemically defined, serum-free medium [Dulbecco’s modified Eagle’s medium (DMEM)/F-12] supplemented with 1% (v/v) Insulin-Transferrin-Selenium-Ethanolamine and 1% (v/v) penicillin-streptomycin-glutamine (Thermo Fisher Scientific, Waltham, MA).

Starting at day 0 of differentiation, hiPSCs were cultured in the presence of 3.5 μM CHIR 99021 (Cayman, Sapphire Bioscience, Redfern, NSW, Australia) for 5 days. The culture medium was then replaced with myogenic differentiation medium supplemented with FGF2 (20 ng/ml; PeproTech—Thermo Fisher Scientific, Waltham, MA) for 14 days. Following this, cells were cultured for an additional 16 days in myogenic differentiation medium (refreshed daily). After 35 days of differentiation, cells were purified by fluorescence-activated cell sorting. Cells were detached using 0.05% (v/v) TrypLe (Thermo Fisher Scientific, Waltham, MA), washed, and incubated with fluorochrome-labeled antibodies at a concentration of 10^6^ cells/ml for 30 min on ice (anti–HNK-1-FITC, 1:100, Aviva Systems Biology, San Diego, CA; anti–c-MET-APC, 1:50, R&D Systems, Minneapolis, MN; PE, 1 μl per 10^6^ cells). After staining, cells were washed, resuspended in myogenic progenitor proliferation medium [DMEM high glucose without pyruvate (Thermo Fisher Scientific, Waltham, MA), 10% (v/v) fetal bovine serum (FBS; Thermo Fisher Scientific, Waltham, MA), FGF (100 ng/ml; PeproTech—Thermo Fisher Scientific, Waltham, MA), and 1% (v/v) penicillin-streptomycin-glutamine (Thermo Fisher Scientific, Waltham, MA)], and filtered through a 70-μm strainer. Live/c-MET^+^ cells were sorted using a 100-μm nozzle and collected in ice-cold myogenic progenitor proliferation supplemented with 1× RevitaCell Supplement (Thermo Fisher Scientific, Waltham, MA). The sorted cells were plated on Matrigel-coated dishes in myogenic progenitor proliferation medium, which was refreshed daily, and maintained at 37°C with 5% O_2_ and 5% CO_2_ until they reached 90% confluence. The differentiation of hiPSCs into myoblasts was assessed by immunofluorescence microscopy staining for MyoD.

Where indicated, 80 to 90% confluent hiPSC-derived myoblasts were switched to myogenic differentiation medium composed of DMEM/F12 (Thermo Fisher Scientific, Waltham, MA; catalog no. 11320033) supplemented with 2% KnockOut Serum Replacement (Thermo Fisher Scientific, Waltham, MA; catalog no. 10828010), 1× ITS-X (Thermo Fisher Scientific, Waltham, MA; catalog no. 51500056), 1 μM CHIR99021 (Cayman Chemical, Ann Arbor, MI; catalog no. 13122), 10 μM SB431542 (Cayman Chemical, Ann Arbor, MI; #19136), 10 μM prednisolone (Sigma-Aldrich, St. Louis, MO; catalog no. P6004), and 0.2% (v/v) penicillin-streptomycin (Thermo Fisher Scientific, Waltham, MA; catalog no. 15140122) at 37°C with 5% O_2_ and 5% CO_2_. At the onset of differentiation (day 0), cells were additionally supplemented with 2.8 μM Ara-C (Sigma-Aldrich, St. Louis, MO) to eliminate proliferative nonmyogenic cells. After 48 hours (day 2), the medium was replaced with fresh differentiation medium lacking Ara-C. On day 4, medium was removed and cells were overlaid with 150 μl of Matrigel-Matrix Growth Factor Reduced (Corning, Bio-strategy catalog no. BDAA354230) diluted 1:1 in differentiation medium. After 40 min, 1 ml of differentiation medium was gently added on top of the Matrigel layer. Cultures were maintained at 37°C with 5% O_2_ and 5% CO_2_, and the medium was refreshed every other day. Terminal differentiation into multinucleated myotubes was achieved by day 8 postdifferentiation, at which point cultures were used for live imaging, fixed for immunostaining, or lysed for protein extraction.

The immortalized human myoblast cell line AB1190 was derived from human paravertebral muscle from a 16-year-old healthy male by Myobank-AFM (affiliated with EuroBioBank) in accordance with European recommendations and French legislation. Myoblasts were isolated by the explant culture method and were then immortalized by lentiviral transduction with cyclin-dependent kinase 4 and human telomerase reverse transcriptase expressing vectors as described previously (*110*). Cells were cultured in medium containing 0.4 volumes of DMEM–high glucose GlutaMAX (Thermo Fisher Scientific, San José, CA, USA) plus 0.1 volumes of medium 199 GlutaMAX supplement (Thermo Fisher Scientific, San José, CA, USA) supplemented with 20% (v/v) FBS (GIBCO Life Technologies, Carlsbad, CA), Fetuin (25 μg/ml; GIBCO Life Technologies, Carlsbad, CA), hEGF (5 ng/ml; GIBCO Life Technologies, Carlsbad, CA), hFGF (0.5 ng/ml; GIBCO Life Technologies, Carlsbad, CA), insulin (5 μg/ml; Sigma-Aldrich, St. Louis, MO, USA), dexamethasone (0.2 μg/ml; Sigma-Aldrich, St. Louis, MO, USA), and gentamycin (50 μg/ml) at 37°C, 5% O_2_, and 5% CO_2_.

Primary skeletal muscle myoblasts isolated from 3- to 4-week-old *Nox4^fl/fl^* mice have been described previously (*29*) and cultured in Entactin-Collagen-Laminin cell attachment matrix (Merck, Millipore, CA)–coated tissue culture dishes in DMEM–low glucose (Sigma-Aldrich, St. Louis, MO) buffered with Hepes, pH 7.4, supplemented with 20% (v/v) FBS (GIBCO Life Technologies, Carlsbad, CA), hFGFβ (25 ng/ml; Sigma-Aldrich, St. Louis, MO, USA), penicillin (50 U/ml), streptomycin (50 μg/ml), and 2 mM l-glutamine at 37°C, 5% O_2_, and 5% CO_2_.

### Immunofluorescence microscopy

hiPSC-derived myoblasts or myotubes were washed with phosphate-buffered saline (PBS) and fixed with 4% (w/v) paraformaldehyde for 10 min, washed thrice with PBS, permeabilized using 0.5% (w/v) Triton X-100 in PBS for 5 min, and blocked for 30 min in 10% (v/v) goat serum and 5% (w/v) bovine serum albumin in PBS. MyoD and NFE2L2 were stained with rabbit α-MyoD (1:100 Alfagene Bioscience, Fairfield, NJ; catalog no. PA539265, RRID:AB_2555857) or rabbit α-NFE2L2 (1:100; Cell Signaling Technology, Beverly, MA; catalog no. 12721T, RRID:AB_2715528) in blocking solution supplemented with 0.1% (w/v) saponin at 4°C overnight. Cells were then washed thrice with PBS and incubated with goat α-rabbit IgG [H+L] Alexa Fluor 647 (catalog no. A21245, Thermo Fisher Scientific, Waltham, MA), Phalloidin-iFluor (catalog no. ab176756, Abcam, San Francisco, CA), and 4′,6-diamidino-2-phenylindole (DAPI; Thermo Fisher Scientific, Waltham, MA) in blocking solution plus 0.1% (w/v) saponin for 1 hour, before washing and mounting in Fluoromount-G (catalog no. 00-4958-02, Thermo Fisher Scientific, Waltham, MA). Images were acquired using a Leica THUNDER Widefield Fluorescence Microscope using a 40× oil immersion objective. For each condition, three randomly selected regions were imaged, and each region was captured as a 5 × 5 tile scan.

Mitochondrial superoxide production was assessed using live-cell fluorescence imaging with MitoSOX Red Superoxide Indicator (excitation/emission ∼396/610 nm; Invitrogen, catalog no. M36008). Cells were incubated with 1 μM MitoSOX Red for 30 min at 37°C, 5% CO_2_, and 5% O_2_, followed by three washes with warm Hanks’ balanced salt solution. Live-cell imaging was performed immediately after staining using a Leica THUNDER Widefield Microscope equipped with a 40× oil immersion objective, maintaining the cells at 37°C and 5% CO_2_ throughout acquisition. Fluorescence Z-stacks were acquired every 5 min for a total of 1 hour. For each condition, three randomly selected regions were imaged, and each region was collected as a 5 × 5 tile scan. At the end of live imaging, cells were fixed with 4% (w/v) paraformaldehyde (PFA), washed twice with PBS, and permeabilized with 0.5% (v/v) Triton X-100 in PBS for 5 min. Cells were incubated with phalloidin-iFluor (Abcam, San Francisco) and DAPI (Thermo Fisher Scientific, Waltham, MA) in blocking solution containing 0.1% (w/v) saponin for 1 hour at room temperature and washed three times with PBS before mounting in Fluoromount-G (catalog no. 00-4958-02, Thermo Fisher Scientific, Waltham, MA). Final Z-stack fluorescence images were acquired on the Leica THUNDER Widefield Microscope using the same 40× objective, imaging three randomly selected regions per condition, each captured as a 5 × 5 tile scan.

### CRISPR-Cas9 gene editing

*NOX4* or *KEAP1* in hiPSC-derived myoblasts or in the human immortalized myoblast cell line AB1190 or *Nfe2l2* in primary murine skeletal muscle myoblasts was deleted using CRISPR-Cas9 ribonucleoprotein (RNP) gene editing. Cells were electroporated with recombinant Cas9 (74 pmol Alt-R S.p. Cas9 Nuclease V3; Integrated DNA Technologies, Coralville, IA) precomplexed with short guide RNAs (600 pmol) targeting *NOX4* (GAGGUUAAGAACAGAUGCUG; Synthego, Menio Park, CA), *KEAP1* (5′-CACGGACAACGCTGTCGATC-3′; Integrated DNA Technologies, Coralville, IA), or *Nfe2l2* (5′-AUUUGAUUGACAUCCUUUGG-3′; Synthego, Menio Park, CA) using the P3 Primary Cell 4D-Nucleofector X Kit (Lonza, Basel, Switzerland) according to the manufacturer’s instructions. After electroporation, cells were rested in supplemented DMEM cell media for recovery for 72 hours before expansion and downstream analyses.

### Proteomic analysis of human mitochondrial proteins

Mitochondria isolation: Mitochondria were isolated from frozen skeletal muscle (*111*). Samples were mechanically fragmented using a scalpel in a petri dish, then subjected to trypsin digestion for 30 min at 4°C (0.05% Trypsin and 10 mM EDTA in PBS, pH 7.4). The homogenization process was carried out in A medium (0.32 M sucrose, 1 mM EDTA, and 10 mM tris-HCl, pH 7.4) using a glass tissue grinder with a motor-driven Teflon pestle (Heidolph RZR 2041), applying 20 strokes at 600 rpm. Samples were initially centrifuged at 1000*g* for 5 min at 4°C, with the resulting supernatant subjected to a second centrifugation at 10,000*g* for 10 min at 4°C. The mitochondrial pellets were resuspended in A medium.

Mitochondria-enriched muscle samples underwent protein extraction using CS buffer (Pipes, pH 6.8, MgCl_2_, NaCl, EDTA, sucrose, SDS, and sodium orthovanadate; Biochain Institute Inc. #K3013010-5), freshly supplemented with protease and phosphatase inhibitors. Approximately 100 μg of protein was processed for in-filter reduction and alkylation with iodoacetamide, followed by overnight trypsin digestion (Nanosep Centrifugal Devices with Omega Membrane-10K, PALL). The resulting tryptic peptides were desalted using Oasis HLB cartridges (Waters). Peptide concentration was determined using the Direct Detect infrared-based quantification system (Merck KGaA, Darmstadt, Germany). Extracted proteins (~200 mg) underwent in-filter reduction and alkylation with iodoacetamide, followed by trypsin digestion (Nanosep Centrifugal Devices with Omega Membrane-10K, PALL). The resultant peptides were tandem mass tag (TMT)–labeled according to the manufacturer’s instructions.

#### Liquid chromatography–tandem mass spectrometry

TMT-labeled peptides were loaded onto Evotips and washed before chromatographic separation using an Evosep One HPLC system (Endurance Column 15 cm × 150 μm ID, 1.9 μm beads-EV1106, Evosep) with a 30 SPD preprogrammed gradient. The system was coupled to a stainless steel emitter (30 μm ID). Peptides were ionized in real time and analyzed using an Orbitrap Eclipse Tribrid mass spectrometer (Thermo Fisher Scientific, San José, CA, USA) with a 2-s TopSpeed method. MS spectra were acquired in the Orbitrap analyzer within a 390 to 1700 *m*/*z* (mass/charge ratio) range at a 60,000 FT resolution. HCD fragmentation was performed at 33 eV normalized collision energy, and MS/MS spectra were analyzed at 30,000 resolution in the Orbitrap. A dynamic exclusion of 20 s was applied.

#### Protein identification and quantification

For peptide identification, MS/MS spectra were analyzed using the SEQUEST HT algorithm within Proteome Discoverer 2.1 (*112*) (Thermo Fisher Scientific) against the Swiss-Prot database (*113*) containing mouse protein sequences (mouse_202105_sw.target-decoy.fasta, 185,234 sequences) concatenated with decoy sequences generated via DecoyPyrat (*114*). Trypsin digestion was set to allow a maximum of two missed cleavages. Fixed modifications included cysteine carbamidomethylation (57.021464 Da) and TMT labeling at the N-terminal and lysine residues (304.2071 Da), while methionine oxidation (15.994915 Da) was considered a dynamic modification. The precursor mass tolerance was set at 800 parts per million (ppm), fragment mass tolerance was set at 0.03 Da, and precursor charge states ranged from 2 to 4.

FDR was determined using the corrected Xcorr score (*115*) and the target/decoy competition strategy, applying the picked FDR method at the peptide level (*116*). An additional filter for a precursor mass tolerance of 15 ppm was used (*117*). A 1% FDR threshold was applied for peptide identification.

Quantitative data from TMT reporter intensities were integrated at the spectrum, peptide, and protein levels using the WSPP model (*118*) and the Generic Integration Algorithm (GIA) in the iSanXoT program (*119*). Protein quantitative values were expressed as the standardized variable Zq (normalized log2-ratios expressed in standard deviation units based on estimated variances).

### RNA sequencing

Total RNA was isolated from homogenized mouse gastrocnemius muscle tissue with RNeasy mini kits (Qiagen, Hilden, Germany) and processed for library preparation (PolyA, unstranded) and sequencing on an Illumina Novaseq PE150 by Azenta Life Sciences (Burlington, MA). Sequencing fastq files were processed using the nf-core-rnaseq (v3.10.1) pipeline as implemented in Laxy (*120*), and feature count matrices were used for further analysis. Count matrices for the human datasets were downloaded from GSE159217 (skeletal muscle) and 92rgnswtn8/1 (*121*). Differential expression was analyzed using the Python implementation of DESeq2 (v0.4.2) (*122*), with genes with absolute logFoldChange >0.5 and FDR <0.05 determined to be differentially expressed. Pathway overrepresentation was analyzed using the enrichR wrapper within gseapy (v1.1.0) (*123*). For ranked gene set enrichment analysis (GSEA), DESeq2 summary statistics for each gene were ranked by the Wald statistic, and pathway enrichment was assessed with gseapy after excluding outliers (*P* value = NA) and any duplicated gene names. Heatmaps were generated using the log of the normalized counts generated by DESeq2, and dot plots of specific genes were generated in GraphPad Prism using the normalized counts.

### Real-time PCR

Total RNA was extracted using RNAzol (Sigma-Aldrich, St. Louis, MO), and its quality and quantity were assessed using a NanoDrop 3300 spectrophotometer (Thermo Fisher Scientific, Waltham, MA). Complementary DNA (cDNA) was synthesized from mRNA using the High-Capacity cDNA Reverse Transcription Kit (Thermo Fisher Scientific, Waltham, MA). qPCR was performed using either the TaqMan Universal PCR Master Mix with TaqMan Gene Expression probes (Thermo Fisher Scientific, Waltham, MA) or the Quantinova SYBR Green Master Mix (Qiagen, Hilden, Germany) with either Bio-Rad Prime PCR primers, or validated oligonucleotide primers. Housekeeping genes *Gapdh* or *Rn18s* were used as internal controls for tissue samples, while *Rplp0* was used for cell samples. *Rn18s* was used for the aging studies. Thermal cycling and fluorescence signal detection were conducted using the QuantStudio 5 (Thermo Fisher Scientific, Waltham, MA). Relative gene expression levels were determined using the ΔΔCT method. Quantitative real-time PCRs were performed using PrimePCR SYBR Green Assay (Bio-Rad, Hercules, CA) primer sets for *Acta 2* (qMmuCIP0032840), *Atf3* (qMmuCID0040542), *Catalase* (qMmuCIP0031341), *Cxcl1* (qMmuCED0003898), *Cxcl2* (qMmuCED0003898), *Ccl2* (qMmuCED0003785), *Gpnmb* (qMmuCID0022452), *Col1a1* (qMmuCID0021007), *Eif2a* (qMmuCID0040098), *Fasn* (qMmuCID0039674), *Fn1* (qMmuCID0019534), *Gapdh* (qMmuCED0027497), *Gdf15* (qMmuCED0040369), *Il10* (qMmuCID0015452), *Il6* (qMmuCID0005613), *Ifng* (qMmuCID0006268), *Nfe2l2* (qMmuCID0021433), *Nqo1* (qMmuCID0017192), *Nox4* (qMmuCIP0035663), *Prdx1* (qMmuCED0047858), *Prdx1* (qMmuCED0047858), *Prdx3* (qMmuCID0017576), *Scd1* (qMmuCID0011348), *Sod1* (qMmuCIP0036673), *Sod2* (qMmuCIP0006109), *Spp1* (qMmuCED0061675), *Srebp1-c* (qMmuCID0009315), *Tgfb1* (qMmuCEP0053152), and *Tnf* (qMmuCED0004141); validated oligonucleotide primers for *Atf4* (TCCTGAACAGCGAAGTGTTG; ACCCATGAG GTTTCAAGTGC), *Chop1* (GCTGGAAGCCTGGTATGAG; ATGTGCGTGT GACCTCTGTT), *Gadd 34* (AGAGAAGACCAAGGGACGTG; CAGCAAGGAA TGGACTGTG), or *Xbp1* (AAGAACACGCTTGGGAATGG; ACTCCCCTTGGCCTCCAC) from Sigma-Aldrich (St. Louis, MO); *Rn18s* (QT02448075) from Qiagen; or TaqMan Gene Expression Assays (Thermo Fisher Scientific, Waltham, MA) for *Acta2* (Mm01546133_m1), *Catalase* (Mm00437992_m1), *Fasn* (Mm00662319_m1), *Fgf21* (Mm07297622_g1), *Gapdh* (Mm99999915_g1), *Nox4* (Mm00479246_m1), *Scd1* (Mm00772290_m1), *Sod1* (Mm01344233_g1), *Sod2* (Mm01313000_m1), *Srebp1* (Mm00550338_m1), and *Tgfb* (Mm01178820_m1).

### Immunoblotting

Murine or human tissues snap frozen in liquid nitrogen were homogenized using a bead homogenizer (Bead Ruptor 12, Omni International, GA) with 1-mm-diameter zirconia/silica beads (Biospec Products, OK) for 30 to 60 s in 10 to 20 volumes of ice-cold RIPA lysis buffer [50 mM Hepes (pH 7.4), 1% (v/v) Triton X-100, 1% (v/v) sodium deoxycholate, 0.1% (v/v) SDS, 150 mM NaCl, 10% (v/v) glycerol, 1.5 mM MgCl_2_, 1 mM EGTA, 50 mM NaF, leupeptin (5 μg/ml), pepstatin A (1 μg/ml), 1 mM benzamidine, 2 mM phenylmethylsulfonyl fluoride, and 1 mM sodium orthovanadate]. Homogenates were incubated on ice for 30 min and clarified by centrifugation at 16,000*g* for 30 min at 4°C. Clarified tissue lysates were resolved by SDS–polyacrylamide gel electrophoresis and transferred onto polyvinylidene difluoride (PVDF) membranes for immunoblotting. To assess protein carbonylation (OxyBlot), loading controls were from identical lysates resolved in parallel on separate gels. For all other immunoblots, loading controls were derived from the same gels.

### Skeletal muscle histology and immunostaining

Gastrocnemius muscles were dissected from 6-, 12-, and 20-month-old mice, snap frozen in liquid nitrogen–cooled isopentane, and stored at −80°C until required. Transverse muscle cryosections (10 μm) were prepared and used for all histology and immunostaining experiments as described previously (*29*).

H&E and SDH staining were viewed via slide scanning using either an Olympus DotSlide microscope where individual images were stitched together using the VS-ASW program (Olympus) to generate a composite image of the entire muscle section, or using an Aperio Scanscope AT Turbo slide scanner (Leica Biosystems). Tomm20 immunostaining of mitochondria or fiber-type staining in muscle sections were imaged using a Leica SP5 5-Channel confocal microscope. All microscopy was performed at Monash MicroImaging or the Monash Histology Platform, Monash University, Australia. Automated fiber-typing analysis was performed using the MuscleJ2 plugin installed in ImageJ 1.54e (National Institutes of Health, Bethesda, MD, USA).

### Quantification and statistical analysis

Data are presented as mean ± SEM. Statistical significance was determined using two-tailed Student’s *t* tests for pairwise comparisons or one-way/two-way analysis of variance (ANOVA) with multiple comparisons for group-based analyses. When data were not normally distributed, as assessed by Kolmogorov-Smirnov normality test, Mann-Whitney test was performed. A *P* value threshold of *P* < 0.05 was considered statistically significant (*P < 0.05, **P < 0.01, ***P < 0.001, ****P < 0.0001). Sample sizes and detailed statistical parameters for each experiment are indicated in figure legends.
