# Supplementary material for: A decline in skeletal muscle NOX4 abrogates exercise-induced adaptive homeostasis and exacerbates biological aging
Source: Sci Adv. 2026 Jun 10;12(24):eadz1953. doi: 10.1126/sciadv.adz1953 (PMC13251858; doi:10.1126/sciadv.adz1953)
Supplement: Supplementary file 1 — Figs. S1 to S19 Table S1 [file sciadv.adz1953_sm.pdf]

Science Advances
AAAS

Supplementary Materials for

A decline in skeletal muscle NOX4 abrogates exercise-induced adaptive homeostasis and exacerbates biological aging

Chrysovalantou E. Xirouchaki *et al.*

Corresponding author: Tony Tiganis, Tony.Tiganis@monash.edu

Sci. Adv. **12**, eadz1953 (2026)
DOI: 10.1126/sciadv.adz1953

This PDF file includes:

Figs. S1 to S19
Table S1

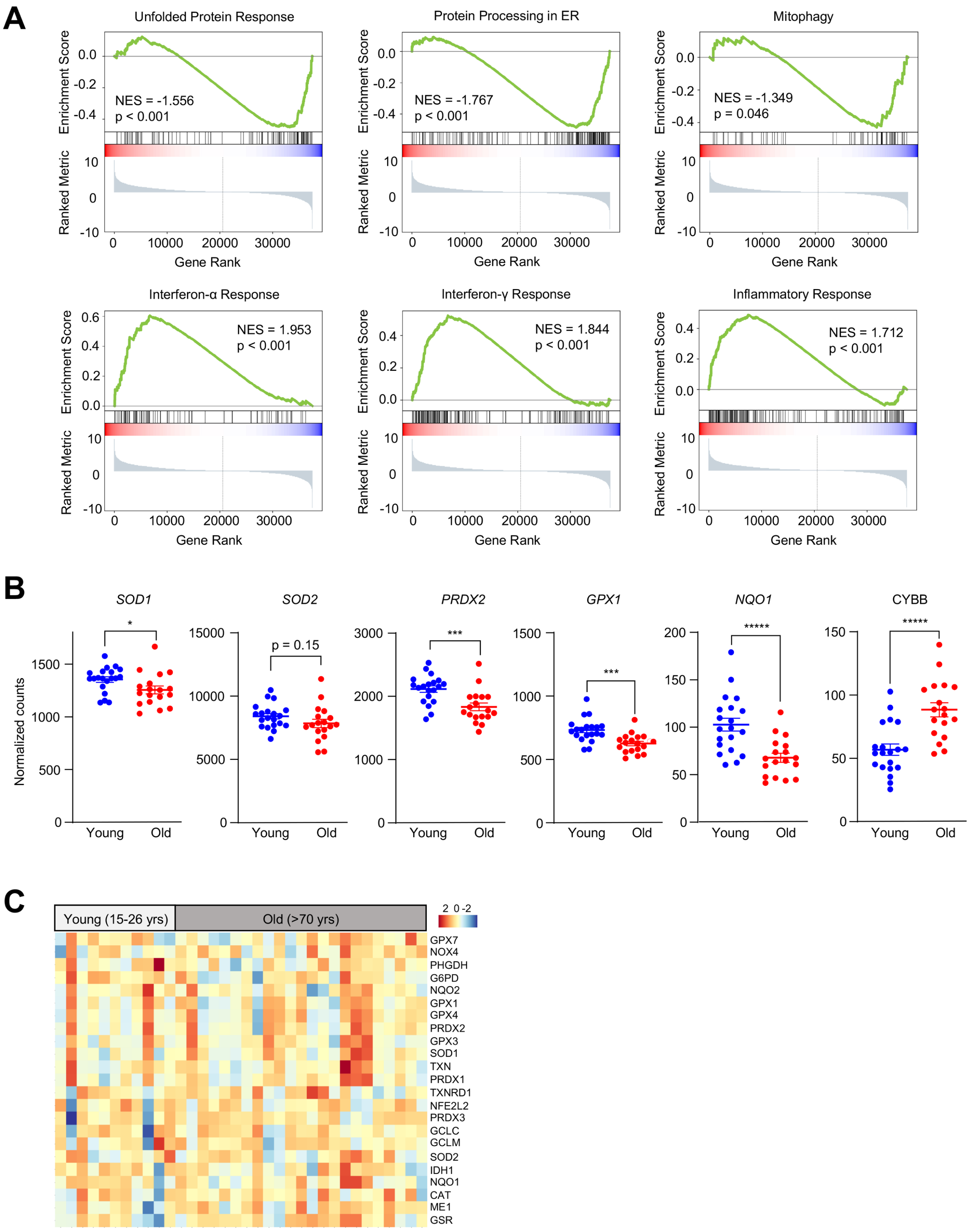

Figure S1. RNAseq analysis of vastus lateralis muscle and hearts in ageing humans - Related to Fig. 1 A-C. **A)** Barcode plots demonstrating negative enrichment of Unfolded Protein Response (Hallmark gene sets) and ER processing and Mitophagy (KEGG Pathways) in older males, and positive enrichment of Interferon α & γ Response and Inflammatory Response (Hallmark gene sets) **B**) Dotplots of DESeq2-normalised counts of key genes from the RNAseq analysis of human male *vastus lateralis* muscle from aged (65-71 years old, n=18) and exercise-matched young (19-25 years old, n=20) subjects. * = p<0.05, *** = p < 0.001, **** = p < 0.0001, ***** p < 0.00001; significance

determined by DEseq2 analysis. Error bars = mean +/- SEM **C**) Heatmap of NFE2L2 Pathway gene expression in RNAseq of heart tissue of 11 young (15-26 years; 7 male, 4 female) and 23 old (70+ years; 8 male, 15 female) humans (GSE141910) [121.]

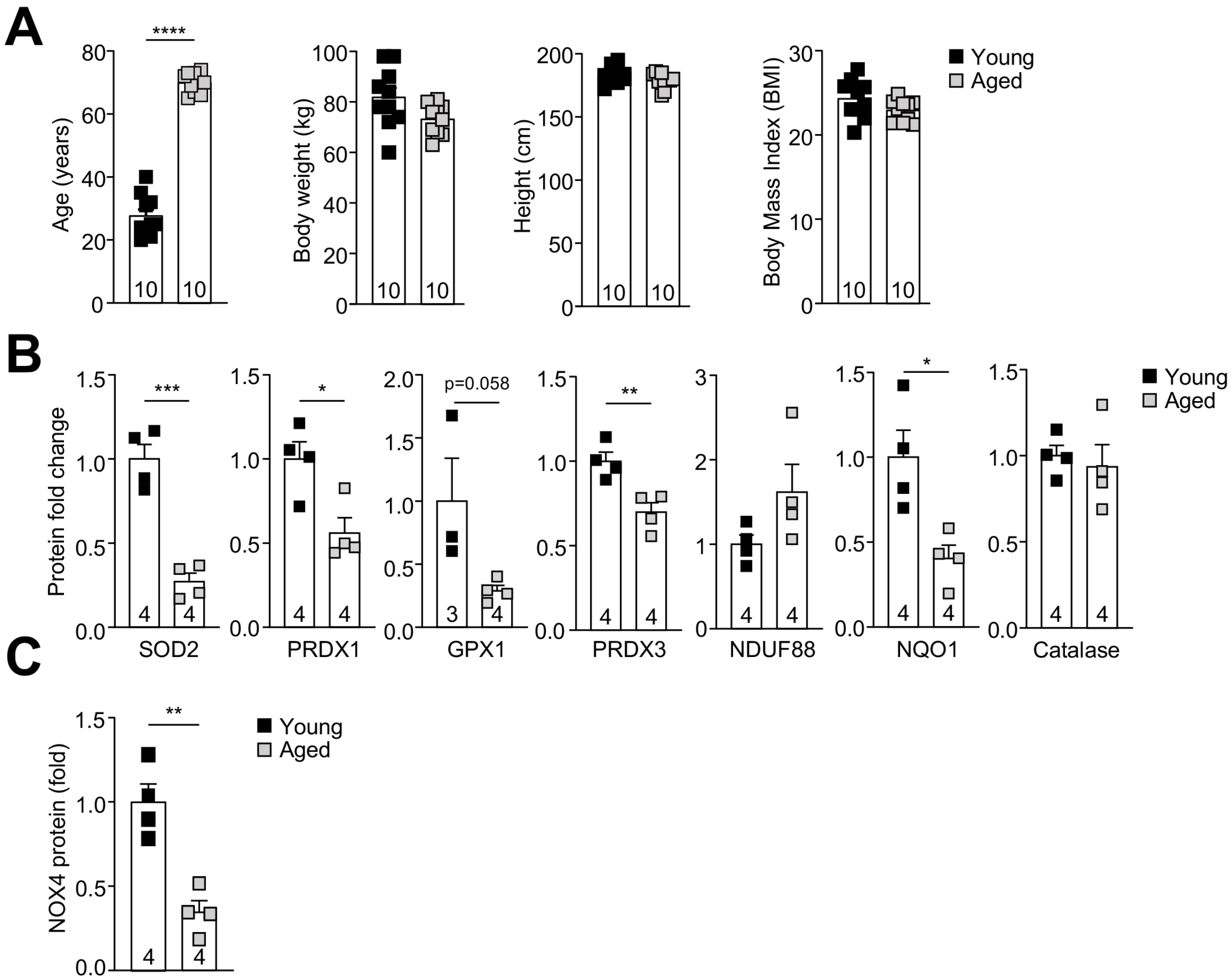

Figure S2. Antioxidant defence proteins decline in vastus lateralis muscles of aged men- Related to Fig. 1d, g-h. Skeletal muscle *vastus lateralis* biopsies from young (27.6 ± 6.6 years old) and aged (69.9 ± 3.1 years old) men were used for analysis of antioxidant defence protein abundance. Anthropometric characteristics of the participants **A**) including age, body weight, height and BMI. **B-C**) Biopsies were processed for immunoblotting to assess the abundance of antioxidant enzymes and NOX4; quantified results of results in b) Figure 1d and c) Figure 1h are shown. Results are mean ± SEM for the indicated number of male participants; significance determined by Student's t-test.

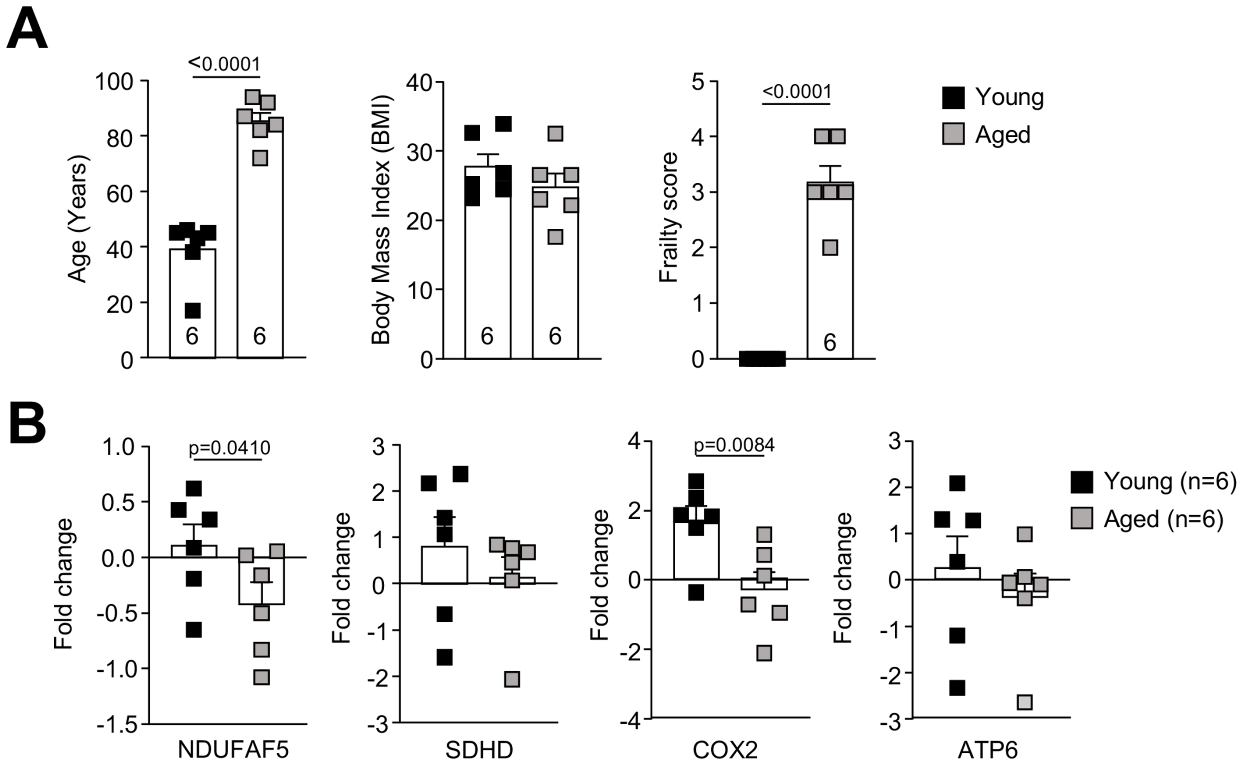

Figure S3. Proteomics analyses of human skeletal muscle mitochondria-enriched fractions - Related to Fig. 1e-f. Mass spectrometry-based proteomic analyses of mitochondria-enriched fractions from hip skeletal muscle biopsies of BMI-matched aged, physically inactive (86.7 ± 9.7-year-old) versus young, physically fit (37.3 ± 10.6 years old) individuals. **A**) Anthropometric characteristics of participants including age, BMI, Valencia Frailty score. **B**) Mitochondria-enriched fractions were analysed for the abundance of oxidative phosphorylation complexes. Representative and quantified results are shown (mean ± SEM) for the indicated number of human mitochondrial fractions; significance determined by Unpaired t test with Welch's correction.

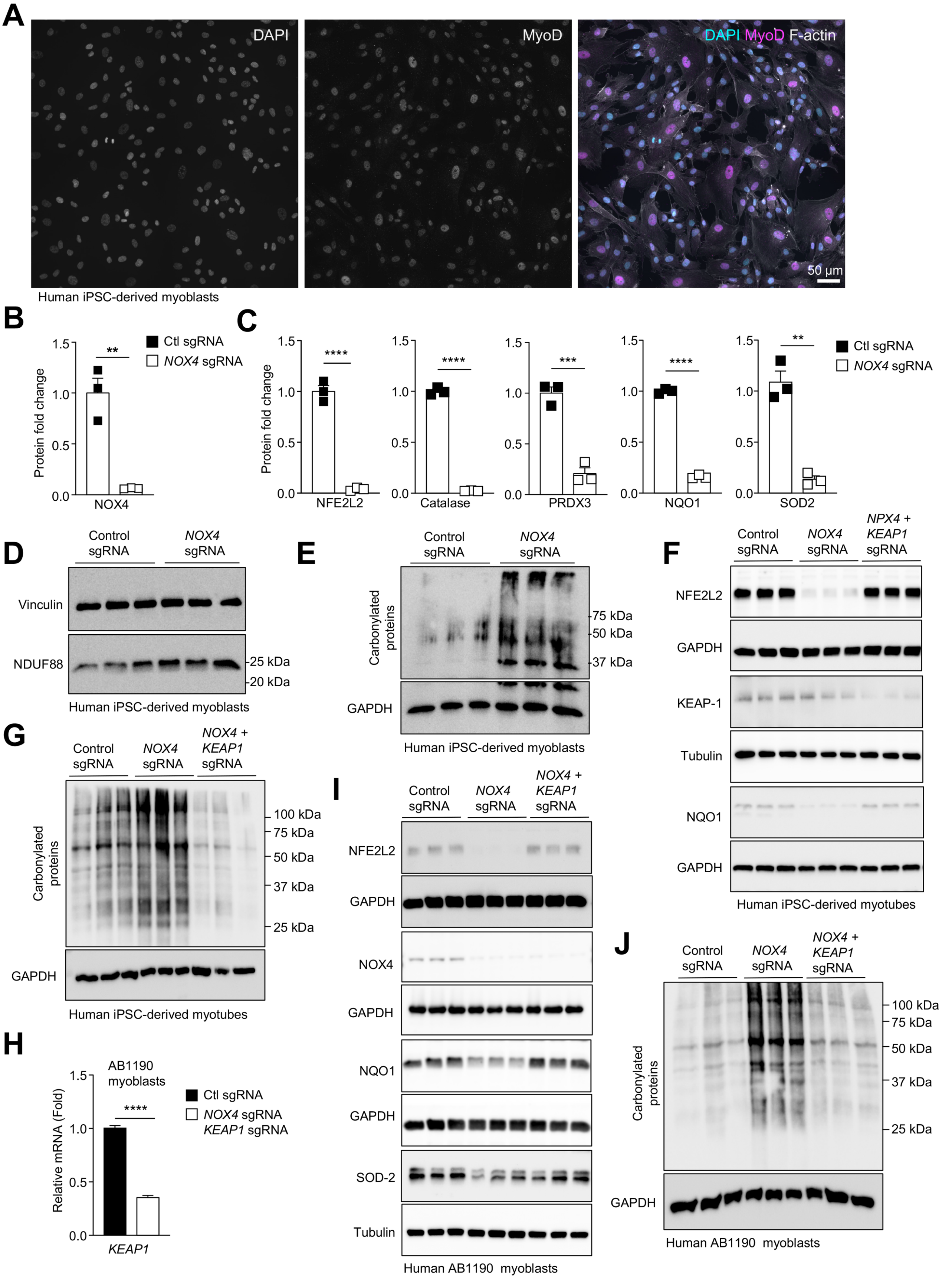

Figure S4. NOX4 deletion in human muscle cells. - related to Fig. 1i. Human induced pluripotent stem cells (iPSCs) were differentiated into myoblasts. **A**) Differentiation into myoblasts was validated by the expression of MyoD assessed by immunofluorescence microscopy staining for MyoD, DAPI and F-actin. **B-D**) *NOX4* was deleted in iPSC-derived myoblasts by CRISPR RNP gene-editing using control or *NOX4* specific sgRNAs and the abundance of **B**) NOX4 protein and **C**) antioxidant defence

proteins assessed by immunoblotting; quantified results of b-c) Figure 1l are shown. Alternatively control and NOX4-deficient iPSC-derived human myoblasts were processed for immunoblotting monitoring for **D**) the abundance of the mitochondrial complex protein NDUF88, or **e**) protein carbonylation. **F-G**) *NOX4* or *NOX4* and *KEAP1* were deleted in iPSC-derived myoblasts by CRISPR RNP gene-editing using control, *NOX4* and *KEAP1* specific sgRNAs as indicated. Control, NOX4- or NOX4/KEAP-1-deficient myoblasts were differentiated into myotubes and processed and **F**) immunoblotted for NFE2L2, KEAP-1 and NQO1 and reprobed for tubulin or GAPDH as indicated, or **G**) resolved on separate gels to immunoblot for protein carbonylation (Oxyblot) and GAPDH. **H-J**) *NOX4* or *NOX4* and *KEAP1* were deleted in human immortalised myoblast cells (AB1190) by CRISPR RNP gene-editing using control, *NOX4* and *KEAP1* specific sgRNAs as indicated. Control, NOX4-or NOX4/KEAP-1-deficient myoblasts were processed for **H**) qPCR, or **I**) immunoblotted for NFE2L2, KEAP-1, NQO1 or SOD-2 and reprobed for tubulin or GAPDH as indicated, or **J**) resolved on separate gels to immunoblot for protein carbonylation (Oxyblot) and GAPDH. Representative and quantified results (means ± SEM) are shown for the indicated number of experiments; significance determined by Student's t-test (b-c).

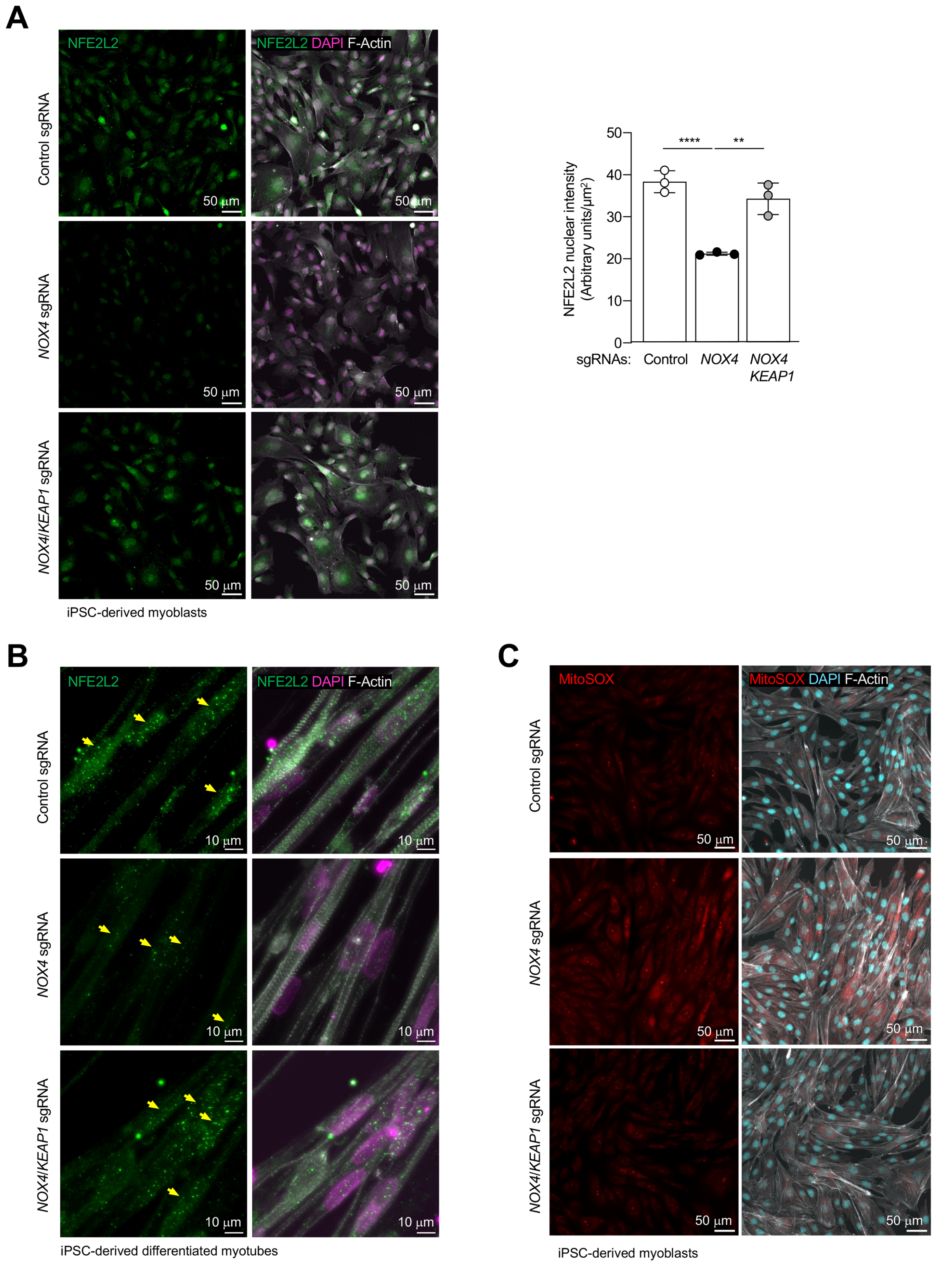

Figure S5. NFE2L2 nuclear localisation and mitochondrial oxidative stress in NOX4-deficient versus NOX4/KEAP-1-deficient human iPSC-derived muscle cells – Related to Fig. 1i. Human induced pluripotent stem cells (iPSCs) were differentiated into myoblasts. NOX4 or NOX4/KEAP1 deletion was achieved by CRISPR RNP gene editing using control, NOX4-specific, or NOX4/KEAP1-specific sgRNAs. **A**) Human iPSC-derived myoblasts were assessed for NFE2L2,

DAPI, and F-actin by immunofluorescence microscopy staining. **B**) Human iPSC-derived myoblasts were differentiated for 8 days into multinucleated myotubes and stained for NFE2L2, DAPI, and α-actinin. **C**) Mitochondrial oxidative distress was assessed in human iPSC-derived myoblasts by live-cell imaging using the mitochondrial superoxide probe MitoSOX Red, followed by fixation and staining for F-actin and DAPI. For panel (a) representative images and the average NFE2L2 nuclear intensity is shown from three randomly selected regions acquired as 5x5 tile scans. Quantified results are means ± SEM for three replicates per condition; significance determined by Student's t-test.

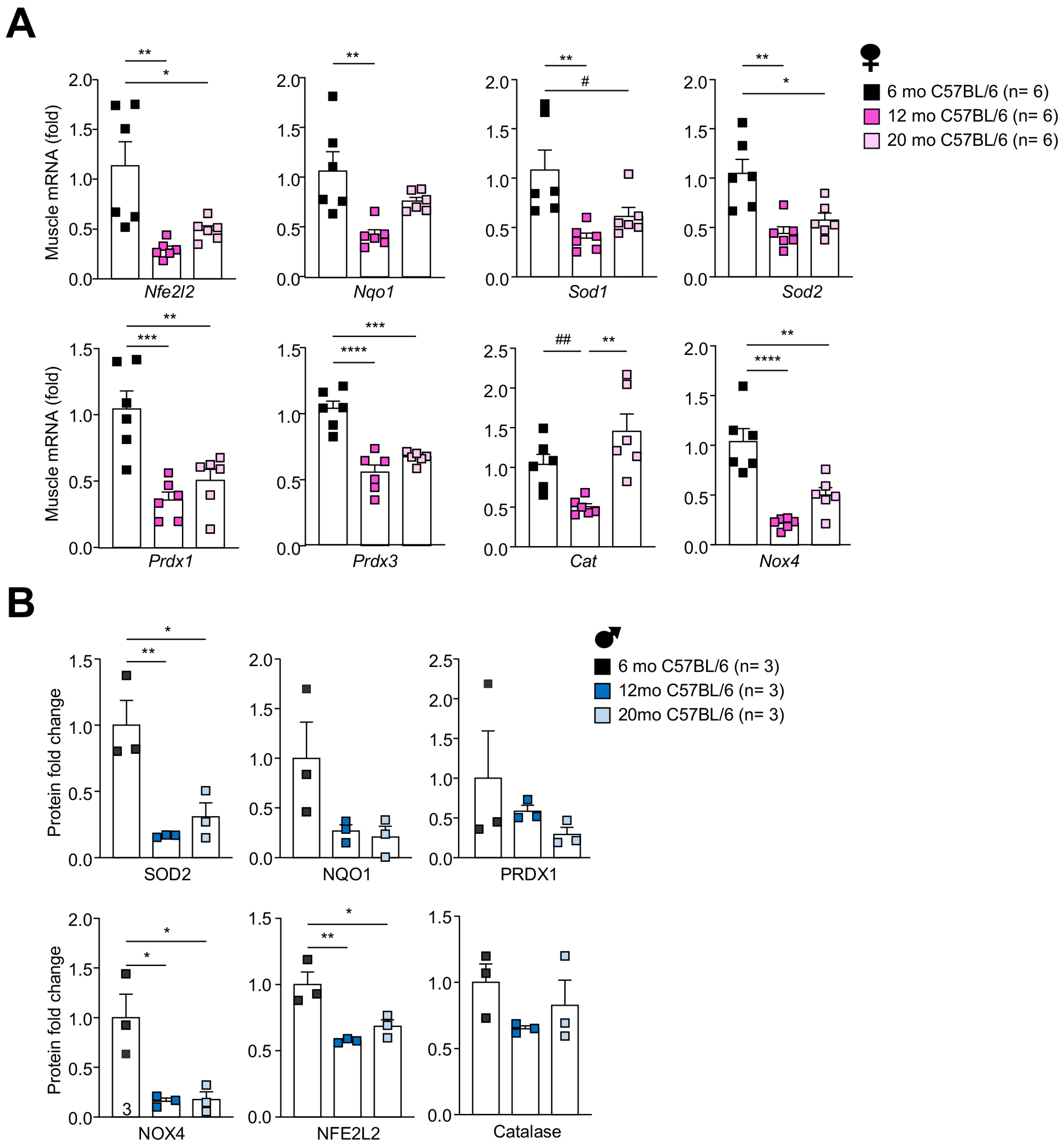

Figure S6. Skeletal muscle antioxidant defence declines during ageing in C57BL/6 mice - Related to Fig 2a-b. **A**) Female C57BL/6 mice were fed a standard chow-diet (4.8% fat) for 6, 12 or 20 months and *gastrocnemius* muscles extracted and analysed by qPCR. **B**) Male C57BL/6 mice were fed a standard chow diet (4.8% fat) for 6, 12 or 20 months and *gastrocnemius* muscles extracted and processed immunoblotting; quantified results of Figure 2c are shown. Results shown are means ± SEM for the indicated number of mice; significance determined using one-way ANOVA or where indicated (#) Student's t-test.

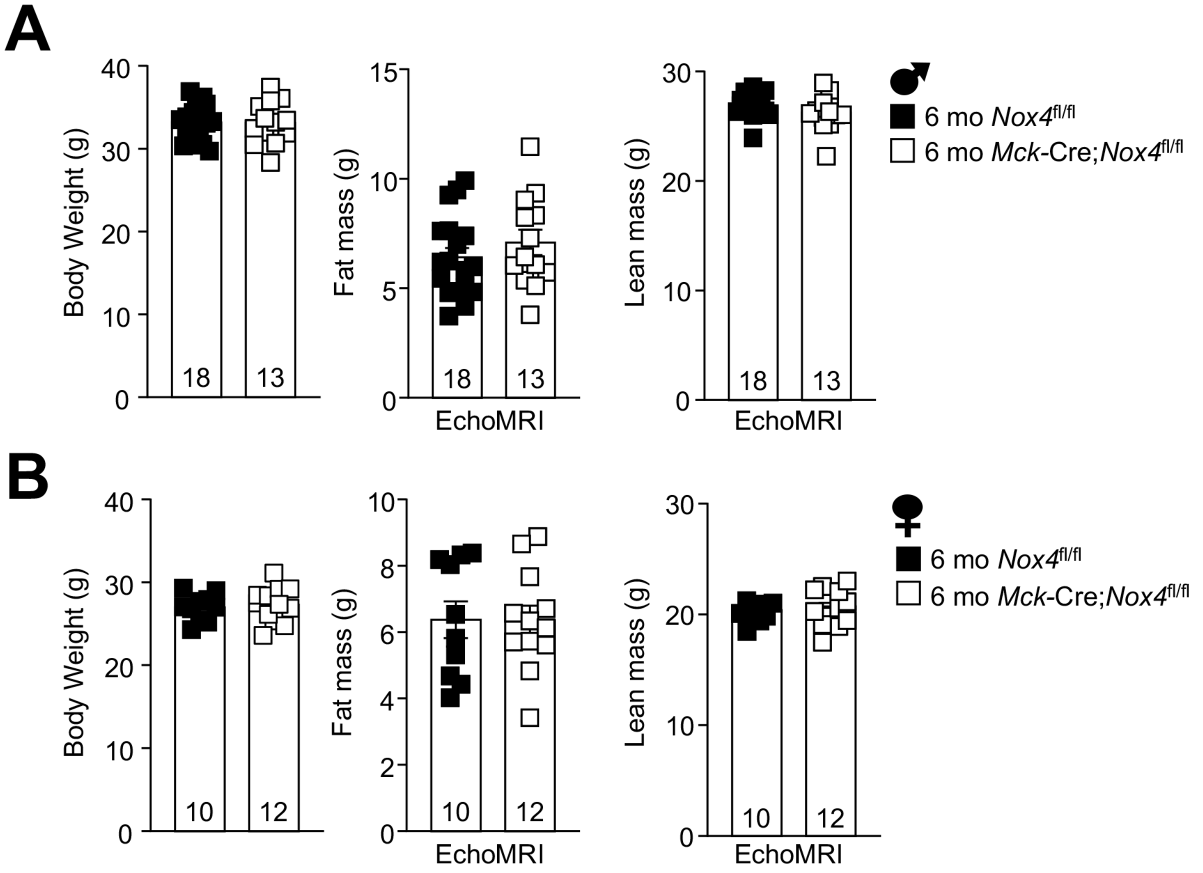

Figure S7. Muscle NOX4-deficiency does not alter body weight or body composition at 6 months of age - Related to Fig 3a-d. Body weights and body composition (EchoMRI) in 6-month-old *Nox4*$^{fl/fl}$ and *Mck*-Cre;*Nox4*$^{fl/fl}$ **A**) male and **B**) female mice fed a standard chow diet (4.8% fat). Results shown are means ± SEM for the indicated number of mice.

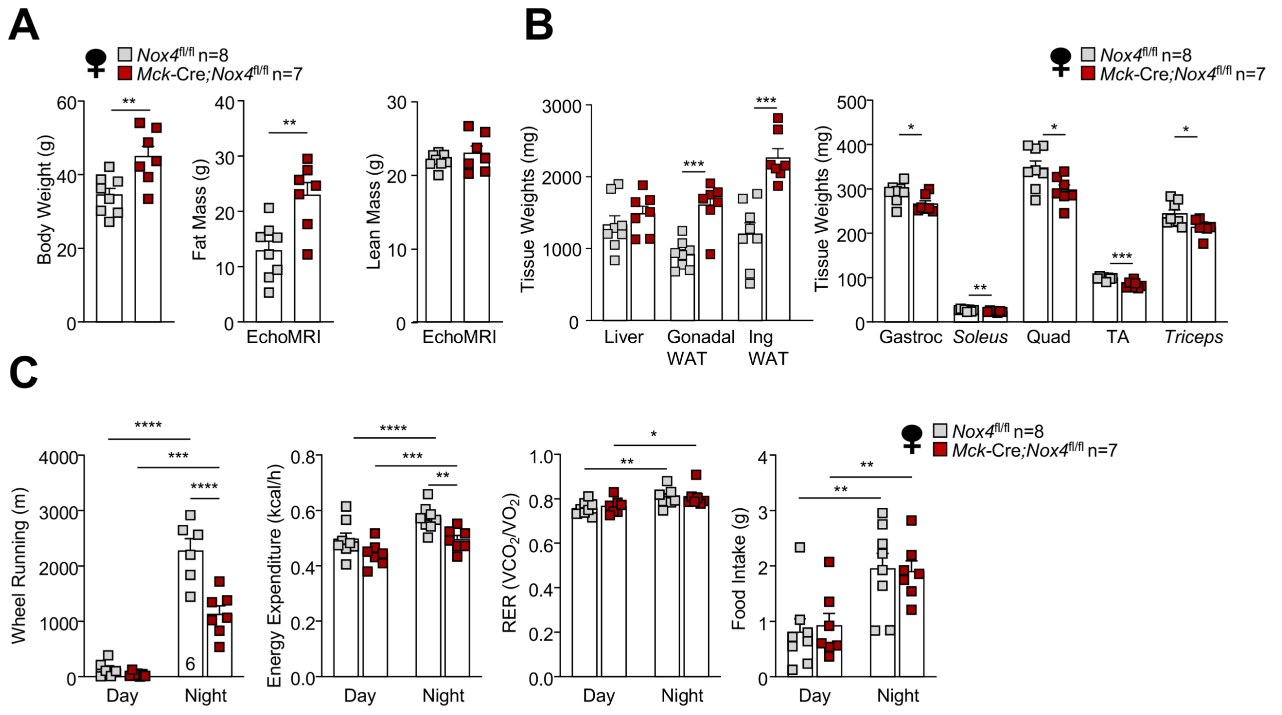

Figure S8. Muscle NOX4-deficiency increases adiposity and decreases muscle mass and voluntary wheel running in aged female mice – Related to Fig. 3a-d. *Nox4*$^{fl/fl}$ and *Mck*-Cre;*Nox4*$^{fl/fl}$ female mice were fed a standard chow diet (4.8% fat) for 20-months. **A**) Body weights and body composition (EchoMRI). **B**) Tissue weights. **C**) Ambulatory activity (wheel running), energy expenditure, RERs and food intake were analysed in metabolic cages (Promethion). Representative and quantified results are shown (means ± SEM) for the indicated number of mice; significance determined by Student's t-test (a, b) or two-way ANOVA (c).

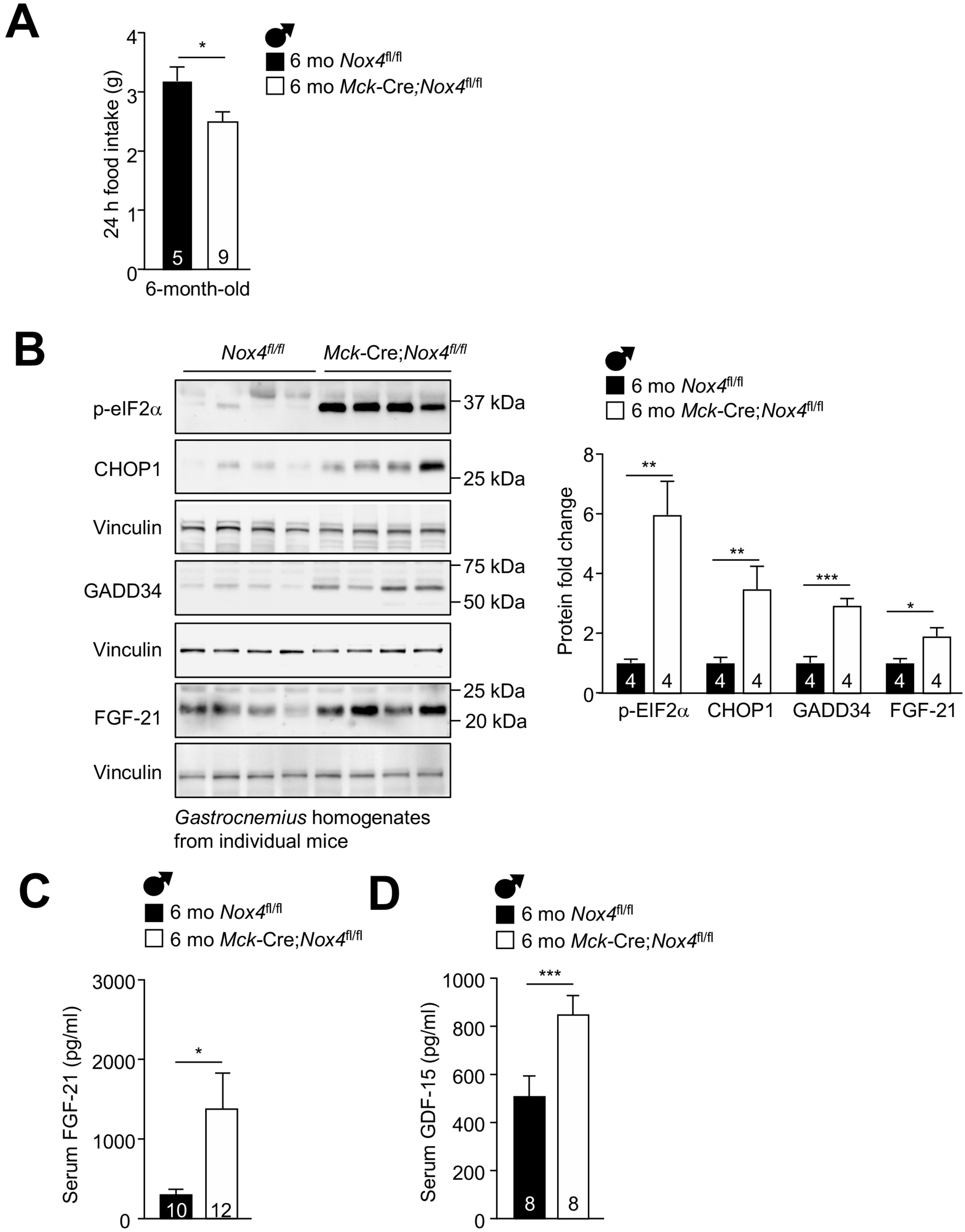

Figure S9. Muscle NOX4-deficiency drives a robust integrated stress response in 6-month-old mice - Related to Fig. 3a-d. *Nox4*$^{fl/fl}$ and *Mck*-Cre;*Nox4*$^{fl/fl}$ male mice were fed a standard chow diet (4.8% fat) for 6 months. **A**) 24 h food intake. **B**) *Gastrocnemius* muscles were processed for immunoblotting to assess eIF2α Ser-51 phosphorylation (p-eIF2α) and CHOP-1, GADD34 and FGF21 protein levels. **C**) Serum FGF-21 and **D**) GDF-15 levels from ad libitum chow fed mice. Representative and quantified results are shown (means ± SEM) for the indicated number of mice; significance determined by Student's t-test.

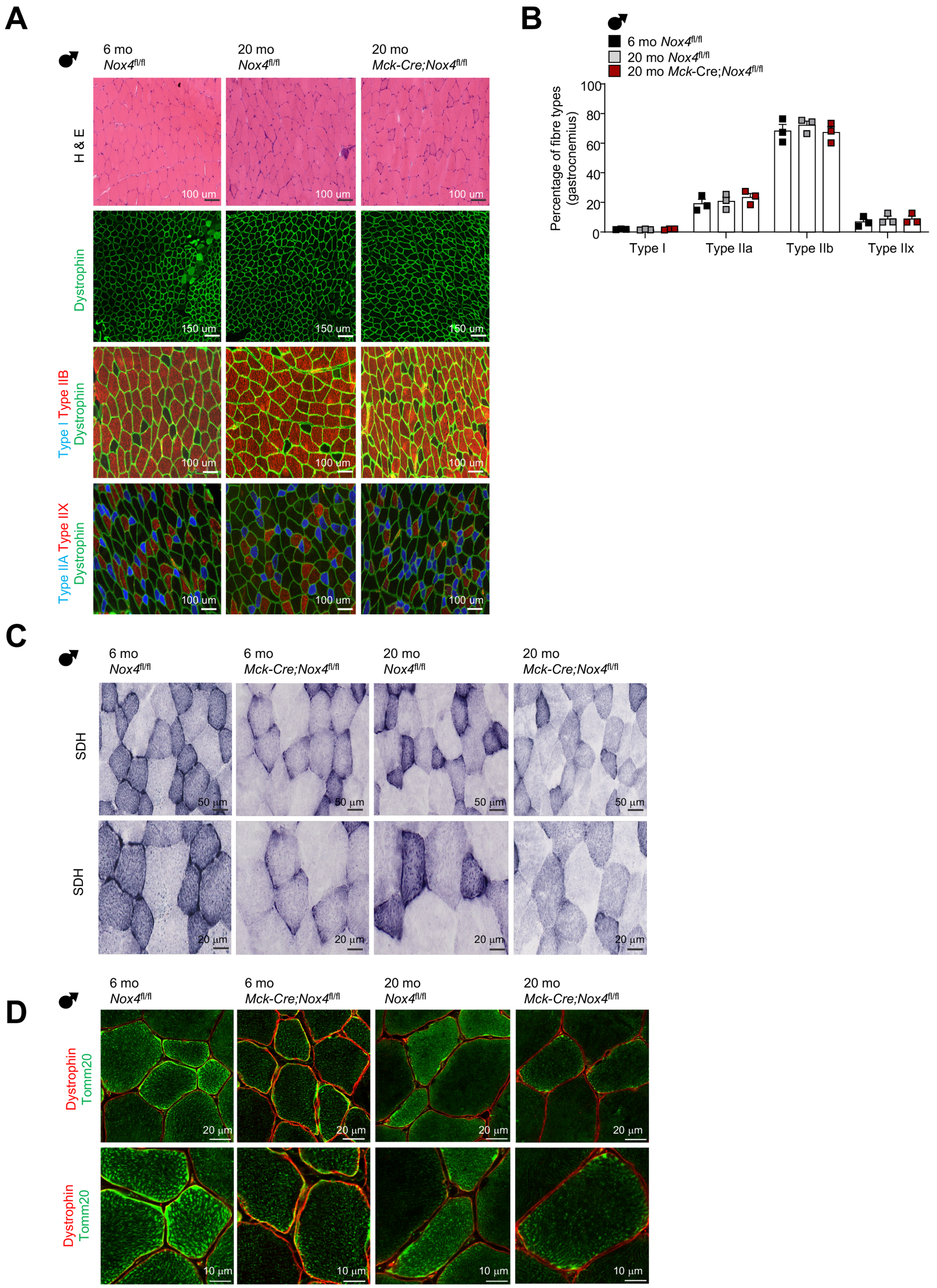

Figure S10. Muscle NOX4-deficiency does not alter fibre type but decreases mitochondrial content in aged mice – Related to Fig 3f-g. *Nox4*$^{fl/fl}$ control and *Mck*-Cre;*Nox4*$^{fl/fl}$ male mice were fed a standard chow diet (4.8% fat) for 6 or 20 months as indicated. **A-B**) *Gastrocnemius* muscles were dissected and processed for immunostaining. Transverse muscle cryosections (10 µm) were processed for a) haematoxylin and eosin (H&E) staining and immunostained for dystrophin, or fibre

types I, or IIa, IIb, IIx and b) fibre type composition determined. **C**) Alternatively, transverse sections were processed for succinate dehydrogenase (SDH) staining or **D**) Tomm20 and dystrophin immunohistochemistry to monitor for mitochondrial content. In d) results shown for 6- and 20-month-old *Nox4*$^{fl/fl}$ controls and 20-month-old *Mck*-Cre;*Nox4*$^{fl/fl}$ mice are the same as those in Fig. 3g. Representative and quantified results are shown (means ± SEM) for the indicated number of mice.

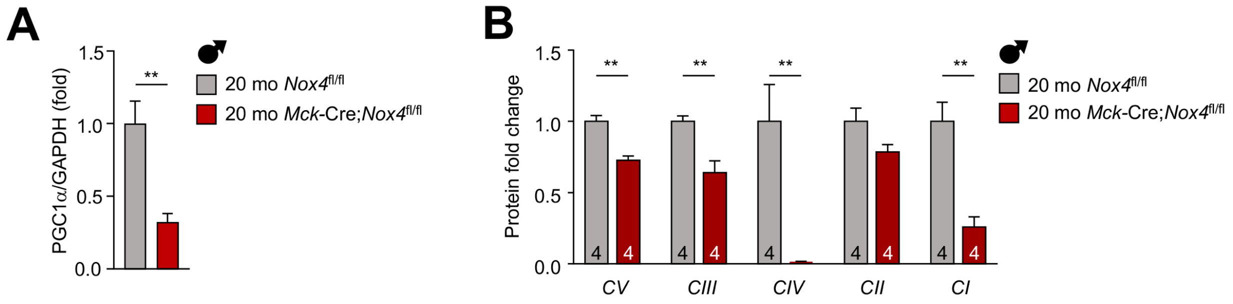

Figure S11 – Muscle NOX4-deficiency decreases mitochondrial content in aged mice Related to Fig. 3f. *Nox4*$^{fl/fl}$ control and *Mck*-Cre;*Nox4*$^{fl/fl}$ muscle-specific NOX4-deficient male mice were fed a standard chow diet (4.8% fat) for 20-months. *Gastrocnemius* muscles were dissected and processed for immunoblotting to monitor for **A**) PGC1α protein levels and **B**) OXPHOS protein complexes; quantified results from Fig. 3f are shown. Results shown are means ± SEM for the indicated number of mice.

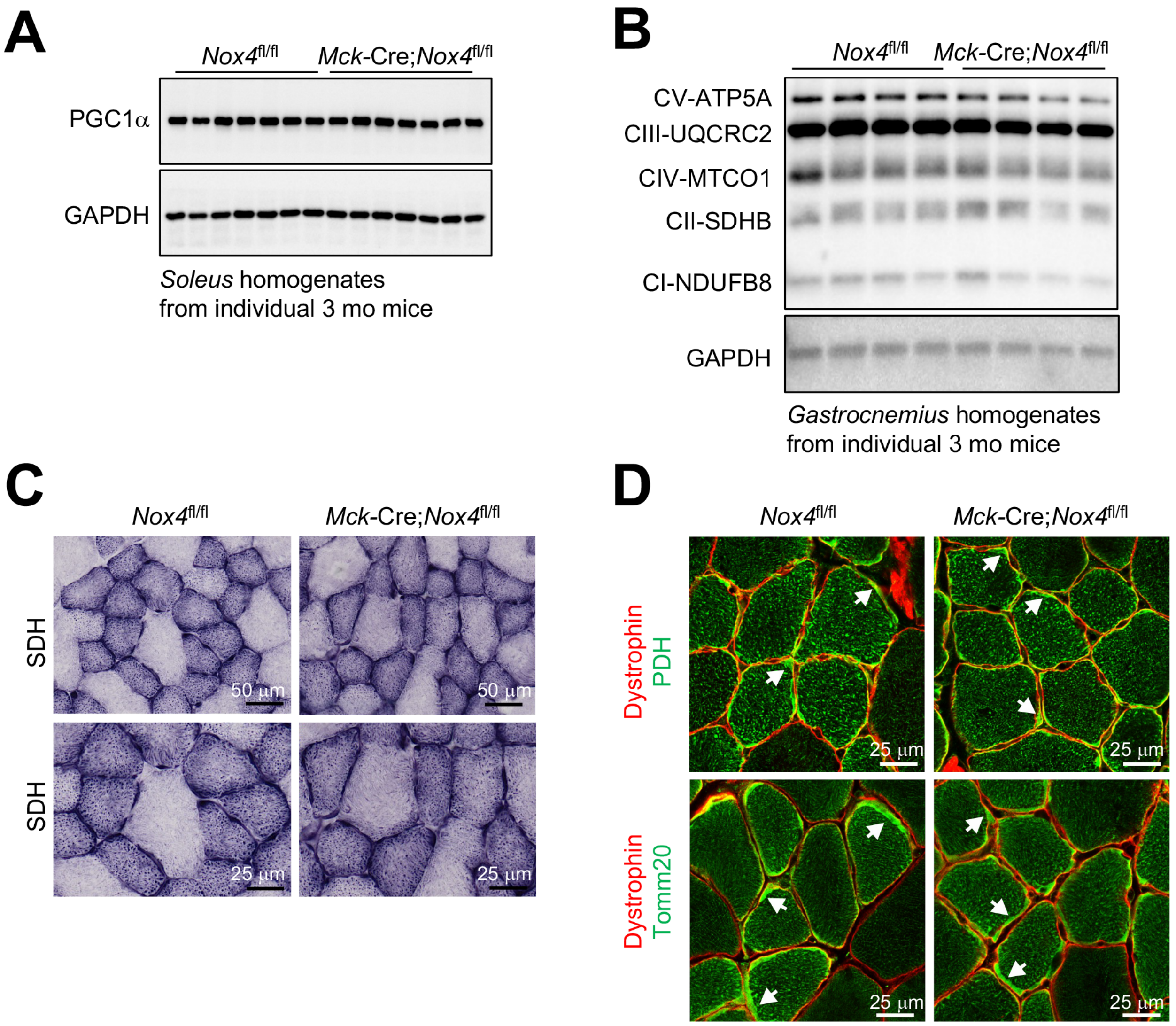

Figure S12 – Muscle NOX4-deficiency does not alter mitochondrial content in 3-month-old mice – Related to 3g. *Nox4*$^{fl/fl}$ control and *Mck*-Cre;*Nox4*$^{fl/fl}$ muscle-specific NOX4-deficient male mice were fed a standard chow diet (4.8% fat) for 3 months. **A**) *Soleus* muscles were dissected and processed for immunoblotting to monitor for PGC1α protein levels. **B-D**) *Gastrocnemius* muscles were dissected and processed for immunoblotting to monitor for **B**) OXPHOS protein complexes, or **C**) SDH staining and **D**) Tomm20/dystrophin or pyruvate dehydrogenase (PDH)/Dystrophin immunohistochemistry to monitor for mitochondrial content.

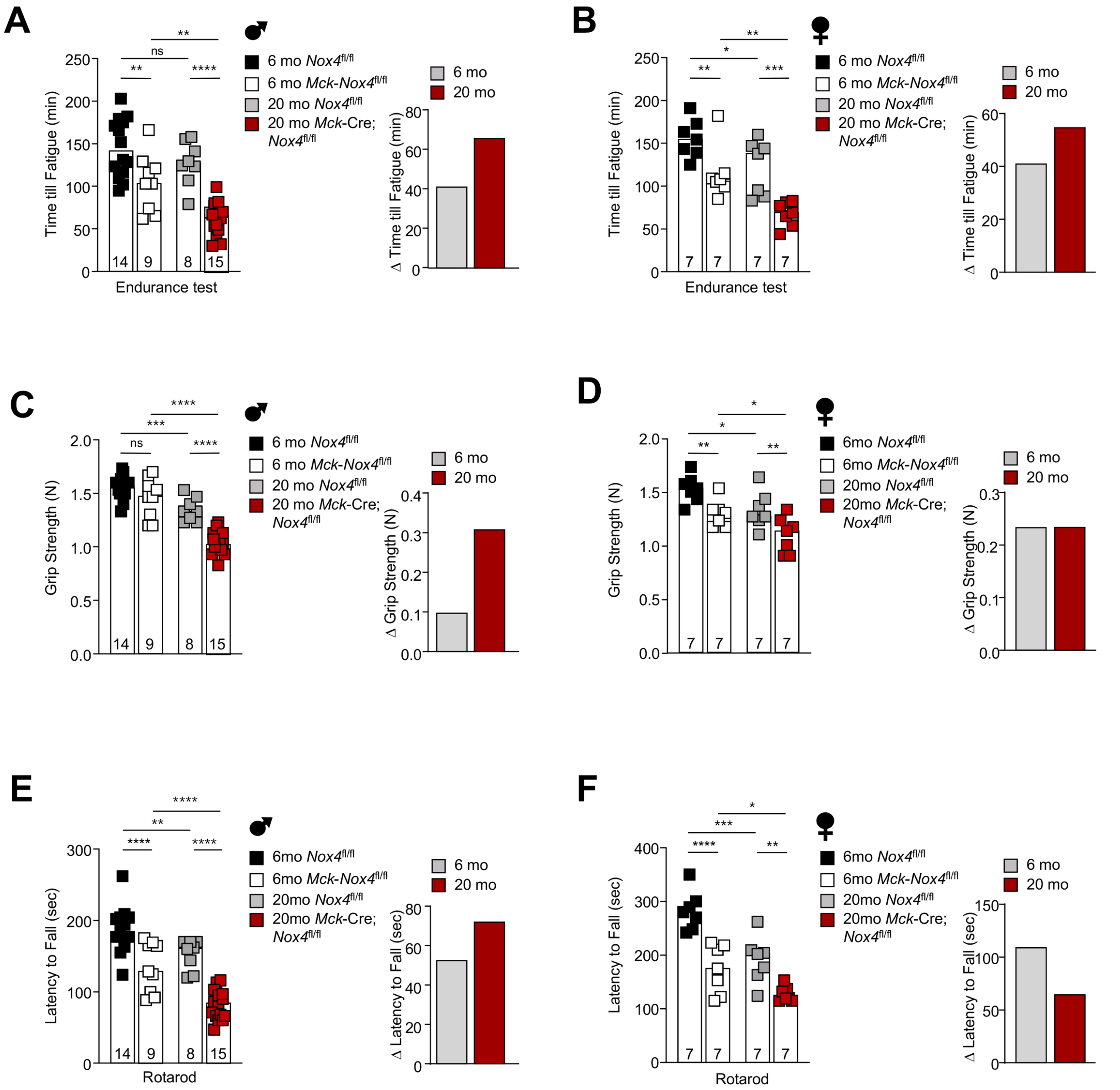

Figure S13. Muscle NOX4-deficiency exacerbates the decline in muscle function in aged mice – Related to Fig. 3h. 6- versus 20-month-old *Nox4*$^{fl/fl}$ and *Mck*-Cre;*Nox4*$^{fl/fl}$ **A, C, E**) male or **B, D, F**) female mice were fed a standard chow diet (4.8% fat) and subjected to **A-B**) endurance tests, **C-D**) grip strength, and **E-F**) motor coordination (rotarod). Calculated differences (Δ values) between *Mck*-Cre;*Nox4*$^{fl/fl}$ and *Nox4*$^{fl/fl}$ mice (*Nox4*$^{fl/fl}$ minus *Mck*-Cre;*Nox4*$^{fl/fl}$) in endurance, grip strength and motor coordination are shown. In a, c, e) results shown for 6- and 20-month-old *Nox4*$^{fl/fl}$ controls and 20-month-old *Mck*-Cre;*Nox4*$^{fl/fl}$ mice are the same as those in Fig. 3h. Results shown are means ± SEM for the indicated number of mice; significance determined using two-way ANOVA.

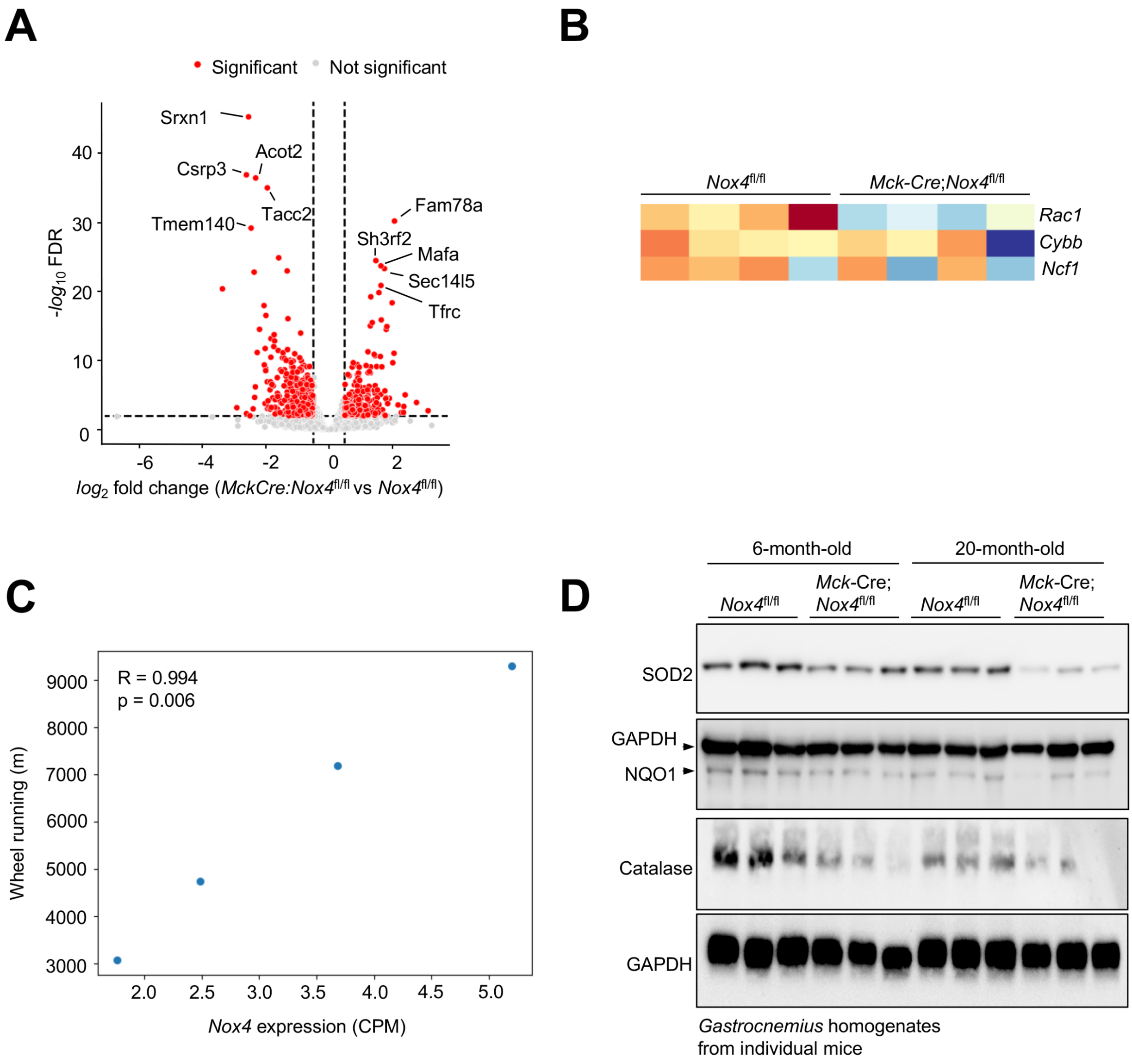

Figure S14. Transcriptomic analysis and antioxidant defence in muscle of NOX4-deficient aged mice - Related to Fig. 4a-c. *Gastrocnemius* muscle from 21-month-old *Nox4*$^{fl/fl}$ and *Mck*-Cre;*Nox4*$^{fl/fl}$ male chow (4.8% fat) fed mice (n=4 per genotype) was processed for bulk RNAseq. **A**) Volcano plot of 443 genes significantly upregulated and 580 significantly downregulated (FDR <0.05, absolute log fold change > 0.5). The top 5 most significant up/downregulated genes are indicated by name. **B**) Heatmap of genes encoding the catalytic (*Cybb*) and regulatory subunits (*Rac1*, *Ncf1*) of NOX2. **C**) Correlation between wheel running distance (m in a 2 day/night period) and *Nox4* expression (counts per million) in 21-month-old *Nox4*$^{fl/fl}$ control mice. Near perfect correlation was observed as assessed by Pearson test (R=0.994, p=0.006). **D**) *Gastrocnemius* muscles from 21-month-old *Nox4*$^{fl/fl}$ and *Mck*-Cre;*Nox4*$^{fl/fl}$ male chow (4.8% fat) fed mice were processed for immunoblotting monitoring for catalase, NQO1 and SOD-2 protein levels.

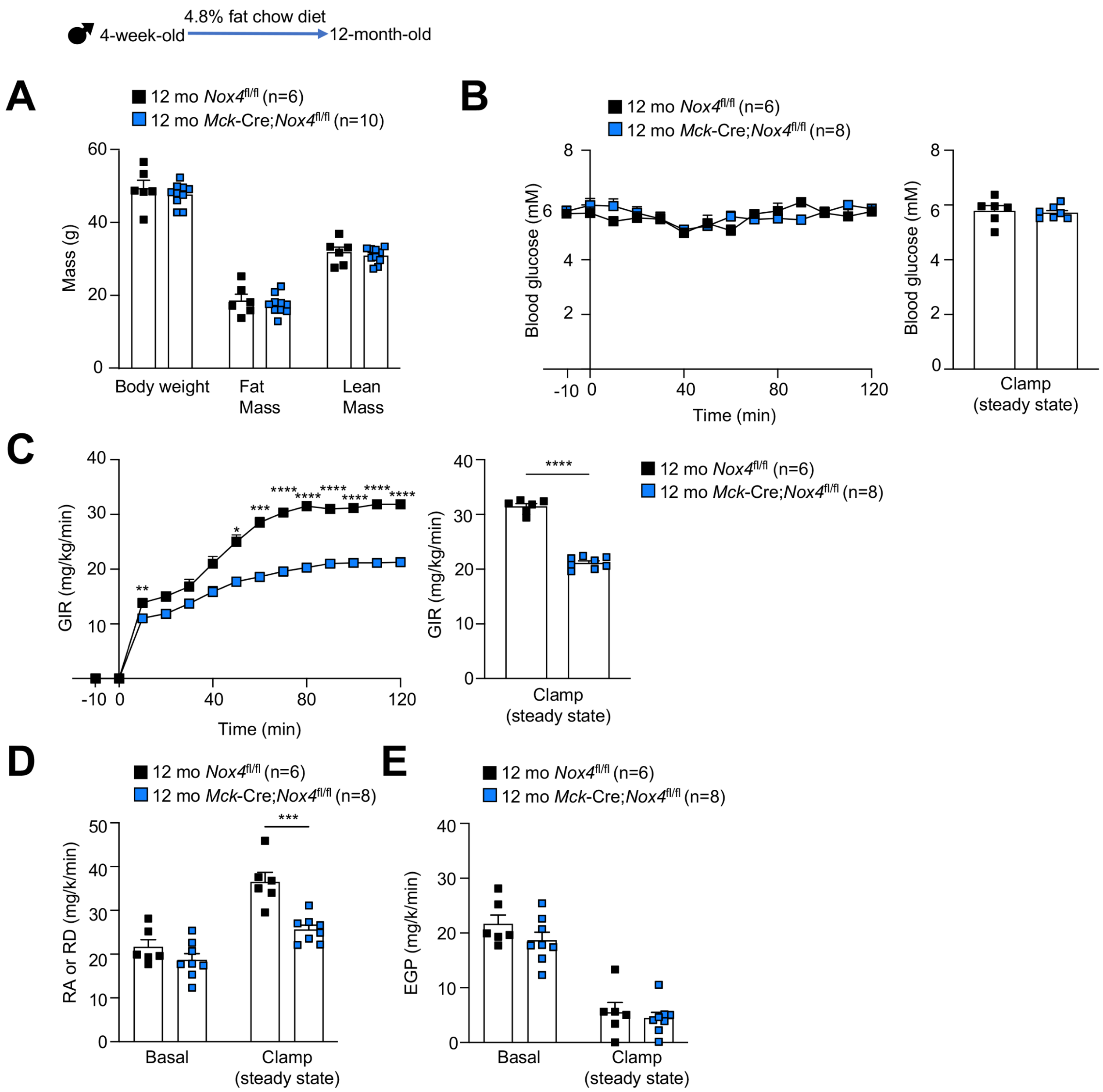

Figure S15. Systemic insulin resistance in 12-month-old muscle NOX4-deficient mice prior to body weight changes. Related to Fig. 5c-e. *Nox4*$^{fl/fl}$ and *Mck*-Cre;*Nox4*$^{fl/fl}$ male mice were fed a standard chow diet (4.8% fat) for 12 months. **A**) Body weights and body composition were assessed. **B-E**) Hyperinsulinaemic-euglycemic clamps in conscious and free-moving mice. **B**) Blood glucose was measured during 120 min (line graph) as well as during the last 30 min of the clamp (80-120 min) where steady state hyperinsulinaemic-euglycaemic conditions were achieved. **C**) The glucose infusion rate (GIR), **D**) rate of glucose disappearance (RD) and **E**) endogenous glucose production (EGP) were assessed. Representative and quantified results are shown (means ± SEM) for the indicated number of mice; significance determined using a two-way ANOVA (d-e) or Student's t-test (c).

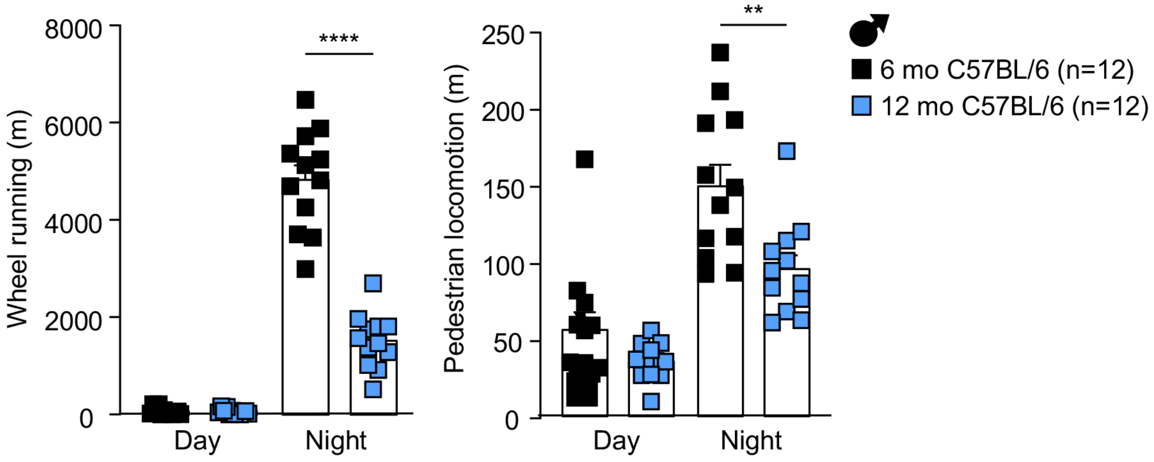

Figure S16. Pedestrian locomotion and wheel running decline in 12-month-old mice -Related to Fig. 6. C57BL/6 male mice were fed a standard chow diet (4.8% fat) for 6 or 12 months and wheel running and pedestrian locomotion assessed in metabolic cages (Promethion). Results shown (means ± SEM) for the indicated number of mice; significance determined using a two-way ANOVA.

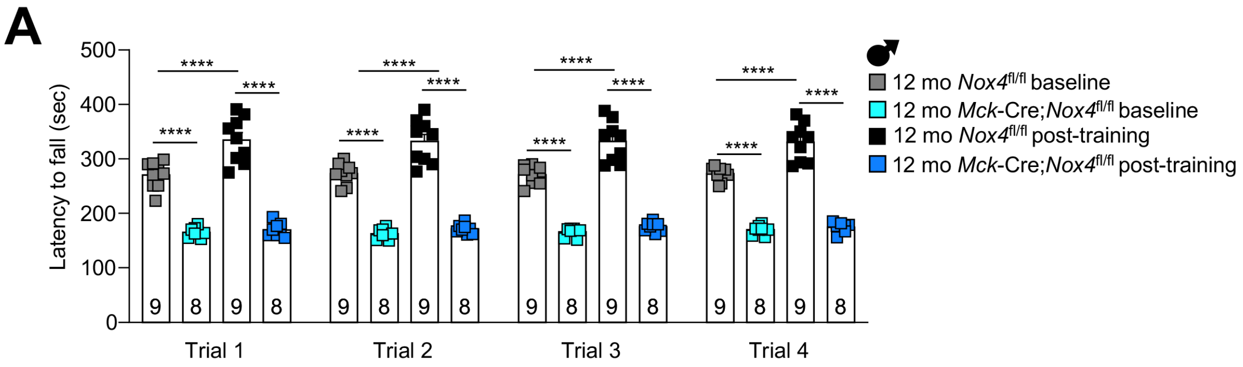

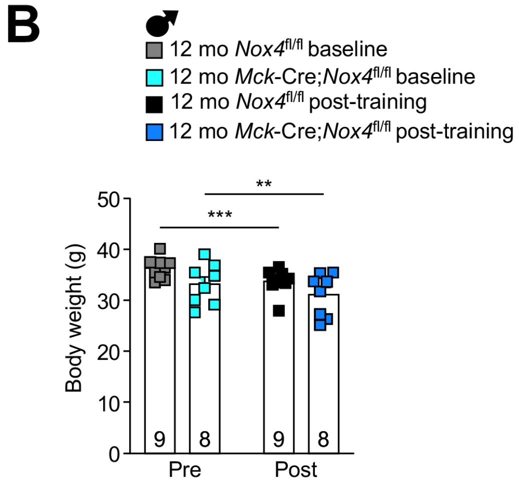

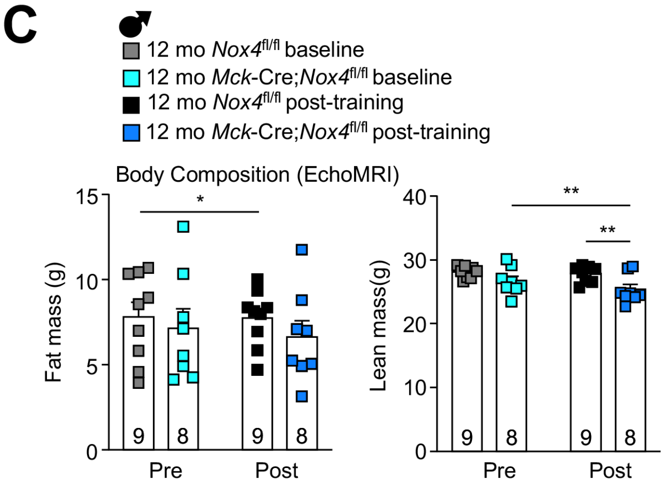

Figure S17. Exercise training does not alter body weight or body composition in muscle from 12-month-old NOX4-deficient male mice - Related to Fig 6e-f. *Nox4*$^{fl/fl}$ and *Mck*-Cre;*Nox4*$^{fl/fl}$ male mice were fed a standard chow diet (4.8% fat) for 12 months and underwent pre and post exercise training (5 weeks with progressively increasing running speeds each week). **A**) Mice subjected to rotarod tests to assess motor coordination pre and post exercise training (trials 1-4 or results shown in Fig. 6e). **B**) Body weights, **C**) body composition (EchoMRI) pre and post exercise training. Results shown means ± SEM for the indicated number of mice; significance determined using two-way ANOVA.

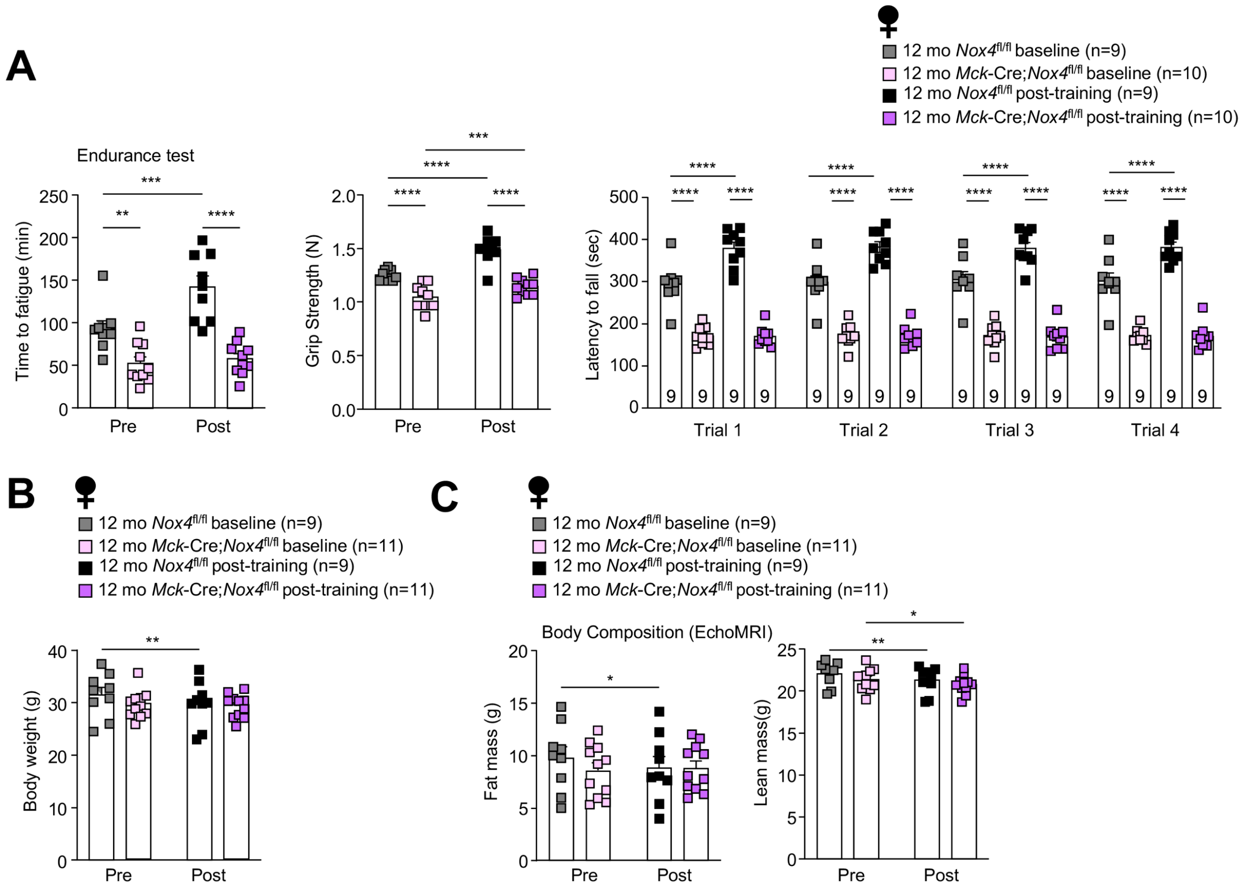

Figure S18. NOX4 is required for the enhanced muscle function in responses to exercise in female mice – Related to Fig. 6e. *Nox4*$^{fl/fl}$ and *Mck*-Cre;*Nox4*$^{fl/fl}$ female male mice were fed a standard chow diet (4.8% fat) for 12 months and underwent basal and post exercise training (5 weeks with progressively increasing running speeds each week) measurements of **A**) endurance, grip strength and motor coordination (rotarod). **B**) Body weights, **C**) body composition (EchoMRI) pre and post exercise training. Results shown means ± SEM for the indicated number of mice; significance determined two-way ANOVA.

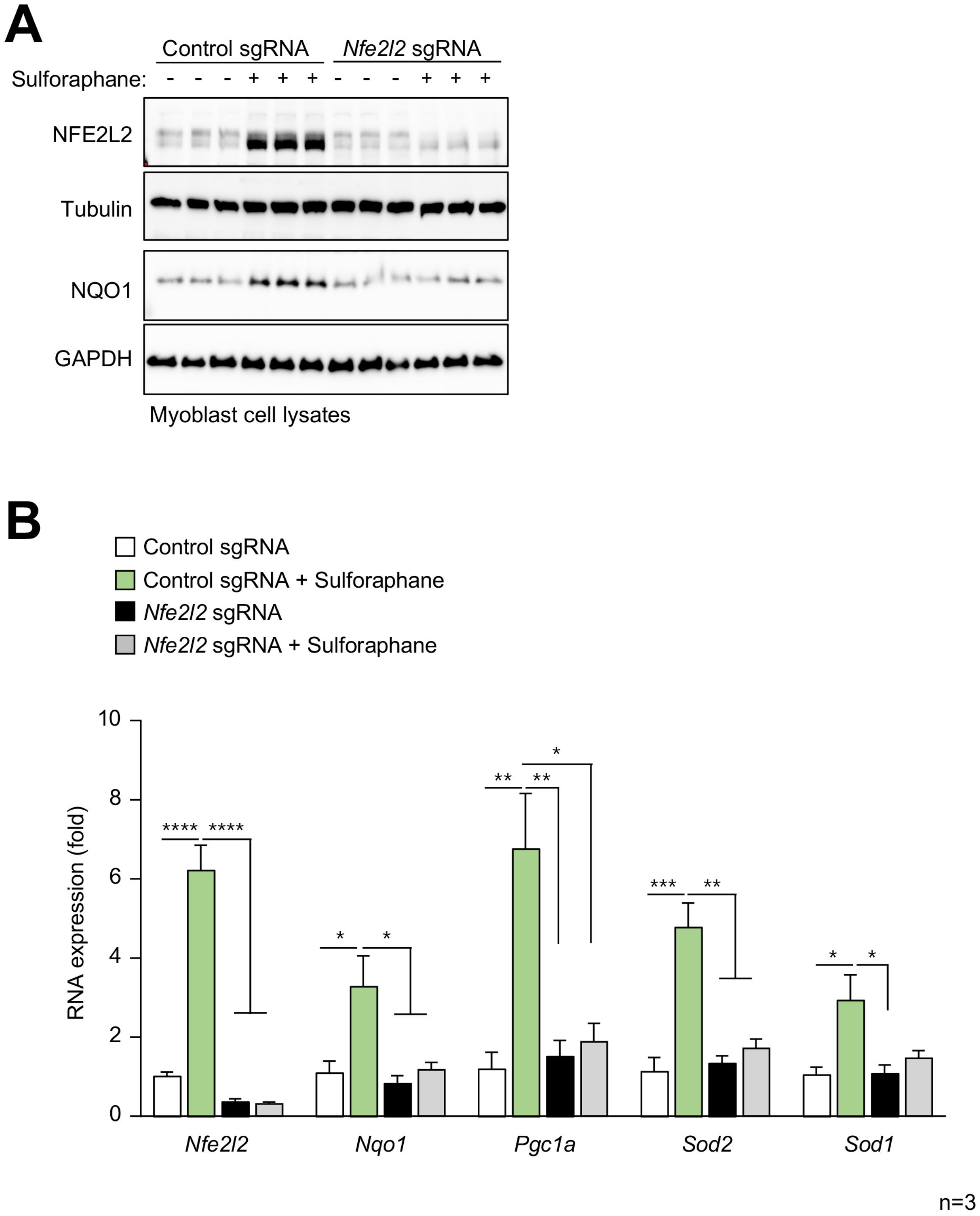

***Fig. S19. NFE2L2-dependent induction of antioxidant defence in murine myoblasts – Related to Fig.** 7*. *Nfe2l2* was deleted in skeletal muscle myoblasts that were FACS-purified from *Nox4*$^{fl/fl}$ mice as described previously [29] by CRISPR RNP gene-editing using control and *Nfe2l2*-specific sgRNAs. Myoblasts were left untreated or stimulated with sulforaphane (5 µM) for 72 h and processed for **A**) immunoblotting or **B**) qPCR. Representative and quantified (means ± SEM) results shown for the indicated number of experiments; significance determined using two-way ANOVA.

Table S1. NFE2L2 gene set used for enrichment analyses

**Mouse**	**Human**
Cat	*CAT*
G6pdx	*G6PD*
Gclc	*GCLC*
Gclm	*GCLM*
Gpx1	*GPX1*
Gpx3	*GPX3*
Gpx4	*GPX4*
Gpx7	*GPX7*
Gsr	*GSR*
Idh1	*IDH1*
Me1	*ME1*
Nfe2l2	*NFE2L2*
Nqo1	*NQO1*
Nqo2	*NQO2*
Phgdh	*PHGDH*
Prdx1	*PRDX1*
Prdx2	*PRDX2*
Prdx3	*PRDX3*
Sod1	*SOD1*
Sod2	*SOD2*
Txn	*TXN*
Txnrd1	*TXNRD1*
